# Effects of a *Saccharomyces uvarum* Cell-Based Preparation on Gut Microbial Ecology and Metabolic Activity in Adults Without Diagnosed Gastrointestinal Disease: A Randomized Placebo-Controlled Crossover Pilot Study

**DOI:** 10.3390/nu18203380

**Published:** 2026-10-15

**Authors:** Mirco Vacca, Mohamad Khalil, Agostino Di Ciaula, Pasquale Filannino, Domenica Maria Di Palo, Francesco Maria Calabrese, Matteo Bernardi, Giuseppe Celano, Ilaria Iacobellis, Gabriella Garruti, Valery Ripari, Enrico Teardo, Maurizio Polo, Maria De Angelis, Piero Portincasa

**Affiliations:** 1 Department of Soil, Plant and Food Science, University of Bari ‘Aldo Moro’, 70121 Bari, Italy; mirco.vacca@uniba.it (M.V.); francesco.calabrese@uniba.it (F.M.C.); matteo.bernardi@uniba.it (M.B.); giuseppe.celano@uniba.it (G.C.); ilaria.iacobellis@uniba.it (I.I.); maria.deangelis@uniba.it (M.D.A.); 2 Internal Medicine Clinica Medica “A. Murri”, Department of Precision and Regenerative Medicine and Ionian Area (DiMePRe-J), University of Bari ‘Aldo Moro’, 70121 Bari, Italy; mohamad.khalil@uniba.it (M.K.); agostinodiciaula@tiscali.it (A.D.C.); domenicamariadipalo@gmail.com (D.M.D.P.); piero.portincasa@uniba.it (P.P.); 3 Section of Internal Medicine, Endocrinology, Andrology and Metabolic Diseases, Department of Precision and Regenerative Medicine and Ionian Area (DiMePRe-J), University of Bari ‘Aldo Moro’, 70121 Bari, Italy; gabriella.garruti@uniba.it; 4 Bioenologia 2.0 S.r.l., 31046 Oderzo, Italy; molecolare@pololab.com (V.R.); lieviti@bioenologia.com (E.T.); info@pololab.com (M.P.)

**Keywords:** *Saccharomyces uvarum*, probiotic yeast, gut microbiota, short-chain fatty acids, metabolomics, crossover clinical trial

## Abstract

**Background/Objectives**: Yeast-based probiotic candidates are increasingly investigated as potential modulators of gut ecology, although evidence in healthy individuals remains limited. This randomized, single-blind, crossover study primarily evaluated the tolerability of ATECREM Serius containing *Saccharomyces uvarum* SERIUS strain (>10^10^ CFU/g) and explored its effect on human gut microbiota. **Methods**: Forty-four volunteers completed the crossover intervention consisting of 2 months of ATECREM Serius (TRT1) and 2 months of placebo (PLT1), separated by 2 weeks of wash-out. Fecal microbiota was profiled by 16S rRNA gene sequencing, while fecal and urinary volatile organic compounds (VOCs) were profiled by GC–MS. Fecal free amino acids (FAAs) were also measured. **Results**: ATECREM Serius was well tolerated, with no treatment-related adverse events. Differences were observed for three out of five α-diversity indices, while β-diversity differed between TRT1 and PLT1. The relative abundance and prevalence of *Bifidobacteriaceae* and *Bacteroides* were increased in TRT1, whereas *Enterobacteriaceae* and clostridia *sensu stricto* were limited compared to placebo. Fecal metabolomics indicated an enrichment of organic acids, particularly SCFAs, in TRT1, whereas propanoic acid was reduced in PLT1. Fecal tryptophan and histidine decreased in TRT1, and urinary metabolomics showed a clearer clustering between TRT0 and TRT1, including a significant decrease of 2,5-dimethylfuran. **Conclusions**: In adults, ATECREM Serius was well tolerated within the investigated population and intervention period. Secondary exploratory analyses identified treatment-associated differences in selected gut microbial taxa and metabolomic features, including the increase in *Bifidobacterium* abundance compared to placebo. These findings are hypothesis-generating and require confirmation in larger, adequately powered studies.

## 1. Introduction

The human gut microbiota is a complex and dynamic ecosystem composed of bacteria, archaea, fungi, and viruses that plays a pivotal role in host physiology, including nutrient metabolism, immune system modulation, and maintenance of intestinal barrier integrity [1]. Alterations in gut microbial composition and functionality have been associated with a wide range of gastrointestinal and systemic disorders, such as inflammatory bowel diseases (IBD), metabolic syndrome, obesity, and functional gastrointestinal disorders [2,3,4,5]. Consequently, dietary strategies aimed at achieving a positive microbiota composition and activity modulation have gained increasing scientific and clinical interest [6,7,8,9].

Among these strategies, the use of probiotics and probiotic-derived food ingredients has emerged as a promising approach to support gastrointestinal health [10,11,12,13,14]. Nowadays, the definition proposed by the Scientific Association of Probiotics and Prebiotics (ISAPP) in 2014 [15], identifying probiotics as viable cells that, when administered in adequate amounts, confer a health benefit to the host, has received broad consensus. However, the use of the term probiotic requires more than the demonstration of microbial viability or potentially beneficial in vitro properties. Binda et al. identified four minimum criteria that a candidate microorganism should fulfill to identify it as a probiotic: it should be sufficiently characterized at the strain level, demonstrated to be safe for its intended use, supported by at least one positive human clinical trial conducted according to generally accepted scientific standards, and present alive in the final product at an efficacious dose throughout its shelf life [16]. These criteria are particularly relevant when investigating novel microbial strains or food ingredients for which clinical evidence is still emerging.

While most commercially available probiotics belong to bacterial groups such as lactic acid bacteria (basonym *Lactobacillus*) and bifidobacteria, increasing attention has been devoted to yeast-based probiotics and specific strains belonging to the genus *Saccharomyces*. *Saccharomyces boulardii* is the most extensively studied probiotic yeast and has demonstrated efficacy in the prevention and management of several gastrointestinal conditions, including antibiotic-associated diarrhea, IBD, and functional bowel disorders [17]. These beneficial effects have been attributed to multiple mechanisms, such as modulation of gut microbiota composition, enhancement of mucosal barrier function, anti-inflammatory activity, and antagonism against pathogenic microorganisms [18,19,20].

ATECREM Serius is a food ingredient based on the *Saccharomyces* (*S.*) *uvarum* SERIUS strain. Recent preclinical evidence has provided further support for the functional potential of *Saccharomyces* (*S.*) *uvarum* SERIUS. Whole-genome sequencing and phylogenetic analysis showed a close relationship with *S. cerevisiae* var. *boulardii*, while genomic screening did not identify high-similarity matches with known antimicrobial-resistance genes [21]. The strain also exhibited limited growth at 37 °C and retained appreciable viability during simulated gastrointestinal transit. In human cell models, *S. uvarum* SERIUS showed strain-specific immunomodulatory and protective effects, including attenuation of pro-inflammatory cytokine release in stimulated Caco-2 cells and PBMCs, partial preservation of intestinal epithelial barrier integrity, and reduction in inflammation- and oxidative-stress-related responses in human keratinocytes. In addition, this strain contributed to phytate degradation under simulated gastrointestinal conditions, suggesting a possible role in improving nutrient uptake for humans. Overall, these findings indicate that *S. uvarum* SERIUS possesses promising safety-related and functional characteristics [21].

However, compared with bacterial probiotics, evidence about the incidence of adverse events of non-*Saccharomyces boulardii* yeasts, in particular of *S. uvarum* SERIUS, and preliminary data concerning the potential effects on gut microbiota composition and host metabolic responses remain limited [22]. In fact, live potential probiotic yeasts are generally well tolerated in individuals, but their administration may require caution in vulnerable populations [23]. Yeast strains naturally tolerate antibacterial antibiotics, possess enzymes such as β-galactosidase and various glycosidases, and may indirectly stimulate bifidobacterial growth, thereby contributing to greater intestinal SCFA production than either microbial group alone [24]. However, the safety assessment should be population-specific, and evidence obtained in volunteers should not be directly extrapolated to high-risk clinical settings since rare cases of fungemia have been reported in immunocompromised patients and in subjects with central venous catheters receiving live yeast-based probiotic preparations [25].

In this context and according to Binda and co-workers [16], before proceeding with additional investigations in specific subsets of human diseases, the present pilot study was designed to evaluate the gastrointestinal tolerability of ATECREM Serius supplementation and its effects on gut microbiota composition and related metabolic parameters in adults without a previous diagnosis of gastrointestinal disease. By integrating microbiota profiling with biochemical and functional readouts, this research aims to provide novel and preliminary insights about the safety and tolerability of oral ATECREM Serius supplementation in hosts and the potential role of *S. uvarum* SERIUS as a functional food ingredient capable of beneficially modulating the intestinal ecosystem.

## 2. Materials and Methods

### 2.1. Study Design

This pilot study employed a randomized, two-period crossover design and was conducted at the Division of Internal Medicine “Augusto Murri,” Department of Precision and Regenerative Medicine and Ionian Area. The intervention phase and sample collection took place between December 2021 and December 2022.

### 2.2. Participants and Eligibility Criteria

A total of 62 adults were screened for eligibility voluntarily. Exclusion criteria included a previous diagnosis of acute or chronic gastrointestinal disease or other conditions potentially affecting the study outcomes, including cancer, unstable cardiovascular or respiratory disease, liver dysfunction, chronic renal failure, and psychiatric disorders. Pregnancy and the use of antibiotics, probiotics, or other medications known to affect the gastrointestinal tract or gut microbiota within the 3 months preceding enrolment were also exclusion criteria. A previous diagnosis of lactose intolerance was not considered an exclusion criterion if the condition was clinically stable and gastrointestinal symptoms had been absent for at least 6 months before enrolment.

Of the 62 individuals screened, 57 met the eligibility criteria, and 50 finally agreed to participate. Non-participation was mainly attributed to concerns about attending hospital facilities following the COVID-19 pandemic. All enrolled participants underwent a comprehensive medical history assessment, physical examination, and anthropometric measurements. Fecal and urine samples were also collected.

Following enrolment, six participants discontinued participation or were excluded because of protocol deviations or circumstances potentially affecting study outcomes. These included COVID-19 infection in participants or household members, the use of antibiotics or other medications affecting the gastrointestinal tract, and withdrawal for personal reasons.

### 2.3. Interventions

Following a 15-day run-in period, participants were randomly assigned to one of two intervention sequences using Randomizer.org (https://randomizer.org, accessed on 4 October 2026): yeast-based treatment followed by placebo (TR–PL), or placebo followed by yeast-based treatment (PL–TR). Each intervention period lasted 2 months, with a 2-week wash-out period between interventions. In accordance with the CONSORT 2025 reporting guidelines [26], a flow diagram depicting participant progress through the trial is provided in Supplementary Figure S1.

The active intervention consisted of ATECREM Serius, supplied by Bioenologia 2.0 S.r.l. (Oderzo, Italy), containing *S. uvarum* SERIUS (>10^10^ CFU/g), water, and xanthan gum at a ratio of 75:24.8:0.2. The placebo formulation contained maltodextrins, xanthan gum, and erythritol mixed with water. The active treatment and placebo are referred to as TR and PL, respectively.

Participants were instructed to incorporate one teaspoon (approximately 5 g) of the assigned formulation into their usual diet during each intervention period. After completing the first 2-month intervention and the subsequent 2-week wash-out period, participants crossed over to the alternative formulation for a further 2 months.

### 2.4. Study Outcomes and Tolerability Assessment

The primary outcome was tolerability, which was evaluated by recording the onset of any adverse event or symptom during the intervention, with particular attention to gastrointestinal symptoms (i.e., abdominal pain and bloating) and altered bowel habits. The absence of treatment-related adverse events was considered indicative of good tolerability within the study population and intervention period, rather than definitive evidence of safety. Exploratory outcomes included changes in gut microbiota composition and in metabolic, biochemical, and functional indices.

### 2.5. Ethical Approval and Informed Consent

The study was conducted in accordance with the Declaration of Helsinki and approved by the Local Ethics Committee of the Azienda Ospedaliero-Universitaria Consorziale–Policlinico di Bari (study No. 7085; approval date: 24 November 2021). All participants provided written informed consent before enrolment.

### 2.6. Dietary and Physical Activity Instructions

Specialized medical personnel instructed participants to maintain their usual dietary and physical activity habits throughout the study, except for the assigned formulation. Participants completed 3-day food diaries covering two weekdays and one weekend day. Diaries were completed at baseline (T0) and at the end of both intervention periods (T1). Trained nutritionists reviewed the diaries for completeness and analyzed them using the Winfood^®^ nutritional analysis software (v.3; Medimatica S.r.l., Colonnella, Italy) to estimate total energy intake and macro- and micronutrient composition. These records were used to assess adherence to the dietary instructions and evaluate whether total energy and macronutrient intake remained comparable across assessment endpoints.

### 2.7. Anthropometric Measurements and Gastrointestinal Symptoms

Physical anthropometric measurements included height (to the nearest 0.5 cm), weight (to the nearest 0.1 kg), and body mass index (BMI), which is reliable, easy to calculate, and correlates with percentage of body fat and body fat mass [27]. BMI is calculated using the following formula: BMI = Weight (kg)/Height (m^2^) [28].

Gastrointestinal symptoms were assessed using a structured questionnaire collecting information on dichotomous variables (symptom present/absent) and prevalence of symptoms in the study group. Severity of each symptom was scored on a 0–100 mm visual analog scale (VAS), with 0 and 100 meaning “total absence of symptoms” and “maximal intensity of the symptom”, respectively [29]. GI symptoms were gastric fullness, epigastric pain, nausea and/or vomiting, heartburn, abdominal bloating, and abdominal pain. Participants were asked to report whether they had experienced each symptom within a defined reference period before assessment. Bowel habits were evaluated based on stool frequency and stool consistency. Stool frequency was recorded as the number of bowel movements per week, reported by participants using recall over the same reference period. Stool consistency was assessed using the validated Bristol Stool Form Scale, a seven-point ordinal scale categorizing stool form from type 1 (separate hard lumps) to type 7 (entirely liquid stool). Participants were instructed to identify the stool type that most closely represented their usual bowel movements [30]. Because the Bristol Stool Scale is not a linear scale of clinical improvement, a higher numerical score was not necessarily considered favorable. Type 4 was used as the target stool form. Improvement was defined bidirectionally as movement toward type 4: an increase from a baseline type below 4 toward type 4 or a decrease from a baseline type above 4 toward type 4. Movement away from type 4 in either direction was not classified as improvement. Participants with type 4 stools at baseline were not classified as improved if they remained at type 4, because their stool form was already at the target category.

### 2.8. Fecal Microbiota Characterization

Following the manufacturer’s protocol, the QIAamp Fast DNA Stool Mini Kit (Qiagen, Hilden, Germany) was used to obtain total genomic DNA from fecal samples. The resulting DNA concentrations were quantified using a Qubit 1.0 Fluorometer (Invitrogen, Carlsbad, CA, USA).

The amplification of the 16S rRNA V3-V4 target gene was carried out using a universal primer pair (Illumina, San Diego, CA, USA). The target gene was amplified using primers targeting the V3-V4 hypervariable regions (forward primer: 5′ TCGTCGGCAGCGTCAGATGTGTATAAGAGACAGCCTACGGGNGGCWGCAG; reverse primer: 5′ GTCTCGTGGGCTCGGAGATGTGTATAAGAGACAGGACTACHVGGGTATCTAATCC) at a final concentration of 1 μM. In addition to the primers, Kapa HiFi HotStart ReadyMix Taq 2× (Kapa Biosystems, Wilmington, MA, USA), a complete reaction mixture, was used. The amplification was performed in a T100 Thermal Cycler (Bio-Rad Laboratories, Inc., Hercules, CA, USA) based on a program consisting of an initial denaturation phase, followed by 25 cycles of denaturation, pairing, extension and a final elongation phase.

The PCR products were purified by magnetic beads Agencourt AMPure XP (Beckman Coulter, Milan, Italy) that selectively bind the desired DNA fragments, allowing for the removal of impurities. This was achieved by washing with 80% ethanol. Purified DNA was eluted in 10 mM Tris-HCl at pH 8.5. The amplicons were then indexed by a second PCR using the Nextera XT indexing kit (Illumina Inc., San Diego, CA, USA) to label the samples and add the adapters necessary to bind to the flow cell. The indexed products were purified, quantified using the Qubit Flex 3.0 fluorimeter (Thermo Fisher Scientific, Milan, Italy), and qualitatively checked with the TapeStation 4200 (Agilent Technologies, Santa Clara, CA, USA). Finally, samples were normalized and aggregated into a pool having a final concentration of 4 nM. Before cartridge loading, the pool was denatured and combined with 30% Phix Control v3 (Illumina). Sequencing was performed on the Illumina MiSeq2 platform in paired-end mode (2 × 300 cycles).

The sequencing was performed at the Dept. of Soil, Plant and Food Sciences (Di.S.S.P.A.) of the University of Bari Aldo Moro. Potential contamination has been avoided, including a negative control (bi-distilled water) in the workflow. Pooled amplicons were subjected to cluster generation and sequenced using the Illumina MiSeq2 platform (San Diego, CA, USA), generating 2 × 250 bp paired-end reads.

Post-sequencing, raw read QC was conducted using FastQC (version 0.12.1; https://www.bioinformatics.babraham.ac.uk/projects/fastqc/ accessed on 10 December 2025). Data processing, including denoising, filtering, and taxonomic classification, was performed using QIIME2 (version 2020.8) with the appropriate plugin suite. Taxonomic assignments were obtained by means of the SILVA database (release 138; https://www.arb-silva.de/documentation/release-138/ accessed on 10 December 2025), enabling identification and quantification of microbial taxa.

FASTQ files were pre-processed using the QIIME2 tool (v. 2025.10) within the Conda environment. Specifically, demultiplexed paired-end reads were first imported, and Illumina adapters and PCR primers were removed using the cutadapt plugin. Quality score plots were inspected to determine appropriate trimming parameters for low-quality bases at the 3′ ends, resulting in 264 bp and 183 bp for forward and reverse reads, respectively. The dada2 denoise-paired plugin included read filtering, denoising, and merging into Amplicon Sequence Variants (ASVs), as well as chimera removal. ASVs were filtered to retain only those present in two or more samples via the filter-features plugin. Finally, taxonomic assignment was performed using a classifier built on the customized 16S V3-V4 SILVA database (release 138.2). Feature tables were rarefied before downstream diversity analyses.

### 2.9. Fecal Metabolome Profiling

Volatile organic compound (VOC) profiling was carried out on both fecal and urine samples. Used as the internal standard (IS), 10 µL of 4-methyl-2-pentanol (final concentration of 9.9 µg/g) was added to samples (1 g) in glass vials (10 mL) sealed with a polytetrafluoroethylene (PTFE)-lined silicone septum before an incubation step (10 min at 40 °C), ensuring headspace equilibration. VOCs were extracted following the conditions and instrumentation specifications previously described [31]. A conditioned 50/30 μm DVB/CAR/PDMS fiber (Supelco, Bellefonte, PA, USA) was used to obtain VOC adsorption. The Clarus 680 (Perkien Elmer, Shelton, CT, USA) gas chromatograph was equipped with a capillary column Rtx-WAX (30 m × 0.25 mm i.d., 0.25 μm film thickness) (Restek, Bellfonte, PA, USA), and a single quadrupole mass spectrometer Clarus SQ 8C (Perkien Elmer) was coupled with the gas chromatography system. The temperature program was 50 °C for 1 min, followed by an increase at a rate of 4.5 °C/min to 65 °C, an increase at a rate of 10 °C/min to 230 °C, and then holding at 230 °C for 25 min. Injection was carried out in splitless mode, and helium (flow rate = 1 mL/min) was used as the carrier gas. VOCs were selected based on a minimum peak area of 1,000,000 and a spectral match score of at least 85%. Manual confirmation of fragmentation patterns was performed when required. Individual VOC concentrations were expressed as equivalents (µg/g) of the IS.

### 2.10. Statistics

Data were expressed as means with standard deviations, medians with ranges, or percentages, and unless explicitly stated, experiments were conducted based on biological triplicates. According to normality/non-normality distributions, verified by Shapiro–Wilk’s test, the comparison of continuous variables accounted for two-tailed *t*-test, Tukey’s test, Wilcoxon signed-rank test, or Mann–Whitney U test corrected for multiple comparisons according to Dunn’s or Benjamini–Hochberg’s statistics (False Discovery Rate, FDR). Volcano plot comparison accounted for pairwise Wilcoxon rank-sum tests. The analysis of variance was carried out by a two-way ANOVA test or by Permutational Multivariate Analysis of Variance (PERMANOVA). Categorical variables were compared by Pearson’s χ^2^ test. For all comparisons, differences were considered significant at *p* < 0.05, with the Volcano plot comparison also accounting for a fold change (FC) > 2. To discover and rank genomic biomarkers (bacterial taxa), a Linear Discriminant Analysis Effect Size (LEfSe) was run in MicrobiomeAnalyst 2.0 [32]. Subsequently to normalization (Z-score), multivariate analyses were carried out by running a Principal Component Analysis (PCA), Partial Least Squares Discriminant Analysis (PLS-DA), Orthogonal Partial Least Squares Discriminant Analysis (OPLS-DA), and a heatmap with hierarchical clustering (based on Euclidean distance coupled with Ward’s method) in an R environment, by running the “FactoMineR” package (Multivariate Exploratory Data Analysis and Data Mining) version 2.12 available in the CRAN repository (https://cran.r-project.org/web/packages/FactoMineR/index.html accessed on 30 May 2026), MicrobiomeAnalyst 2.0, and MetaboAnalyst 6.0 [33]. As the primary inferential analysis of treatment-associated changes in gut microbiota accounting for the crossover design, differential abundance and prevalence analyses were performed using MaAsLin3 in the 13 participants for whom complete paired samples were available at all four study time points [34]. Count data (reads) were normalized by Total Sum Scaling (TSS) and log-transformed before analysis. Treatment, time, period, treatment-by-time interaction, period-by-time interaction, sequence, and sequencing depth were included as fixed effects, while participant ID was included as a random effect. In particular, the Treatment × Time interaction was used to estimate whether the pre-to-post change differed between yeast and placebo. MaAsLin3 separately evaluated taxon abundance and prevalence, with the latter based on presence/absence across samples. *p*-values were adjusted for multiple testing using the Benjamini–Hochberg False Discovery Rate (FDR), and associations were considered significant at q < 0.05.

## 3. Results

### 3.1. Baseline Characteristics of the Study Population

After screening, a final number of 44 subjects who completed the study and were included in the final analysis were enrolled. The two initial groups before the crossover phase consisted of 19 individuals allocated to the placebo sequence and 25 to the treatment sequence. Baseline characteristics of the cohort are summarized in Table 1.

The groups were young and of normal weight, with no differences with respect to gender, age, or BMI (all *p* > 0.05). Minimal comorbidities were infrequent and evenly distributed between groups, including isolated cases of allergy, hypertension, thyroid nodules, lactose intolerance, thyroiditis, and anemia. Similarly, baseline gastrointestinal symptoms were generally uncommon and had a comparable prevalence in the two subgroups. Sporadic abdominal bloating was the most frequently reported symptom, affecting 27.3% of participants, followed by very mild abdominal pain (6.8%), while gastric fullness, epigastric pain, and heartburn were reported only sporadically. No participants reported nausea or vomiting at baseline.

The analysis of the 3-day food diaries collected at baseline (T0) and at the end of both interventions (T1) showed that total energy intake remained stable in all groups throughout the study. Likewise, no significant changes were observed in carbohydrate, protein, total fat, or fiber intake within all groups, indicating that the dietary counseling successfully maintained the habitual dietary pattern of participants (Table 2). These findings support compliance with the dietary intervention and the intended inclusion of the study products.

### 3.2. Changes in Gastrointestinal Symptoms and Bowel Habits

Body weight and BMI remained stable throughout the study. Statistical analysis did not reveal significant differences among the parameters recorded at the different time points. Changes in gastrointestinal symptoms and bowel habits between baseline (T0) and follow-up (T1) are reported in Table 3.

No significant differences existed within either group for abdominal bloating, abdominal pain, or bowel habit frequency. In the placebo group, bloating scores tended to decrease from 14.86 ± 4.80 mm to 8.81 ± 3.07 mm (*p* = 0.317). Similarly, abdominal pain decreased slightly from 1.07 ± 2.86 mm to 0 mm (*p* = 0.8). In the treatment group, bloating and abdominal pain scores showed small, non-significant changes between T0 and T1 (*p* = 0.32 and *p* = 0.93, respectively). The number of bowel movements per week showed a modest increase in both groups, from 7.38 ± 0.50 to 8.00 ± 0.58 in the placebo group and from 7.58 ± 0.53 to 9.02 ± 0.35 in the treatment group, but these differences did not meet statistical significance (*p* = 0.610 and *p* = 0.134, respectively). The distribution of Bristol Stool Scale types at baseline and after each intervention is presented in Table 4.

Type 4 was the most frequent stool form at all assessment points. During the treatment intervention, the proportion of participants reporting type 4 stools increased from 59.1% at T0 to 75.0% at T1. During the placebo intervention, the corresponding proportion changed from 56.8% to 52.3%. These distributions are presented descriptively.

**Figure 1:**
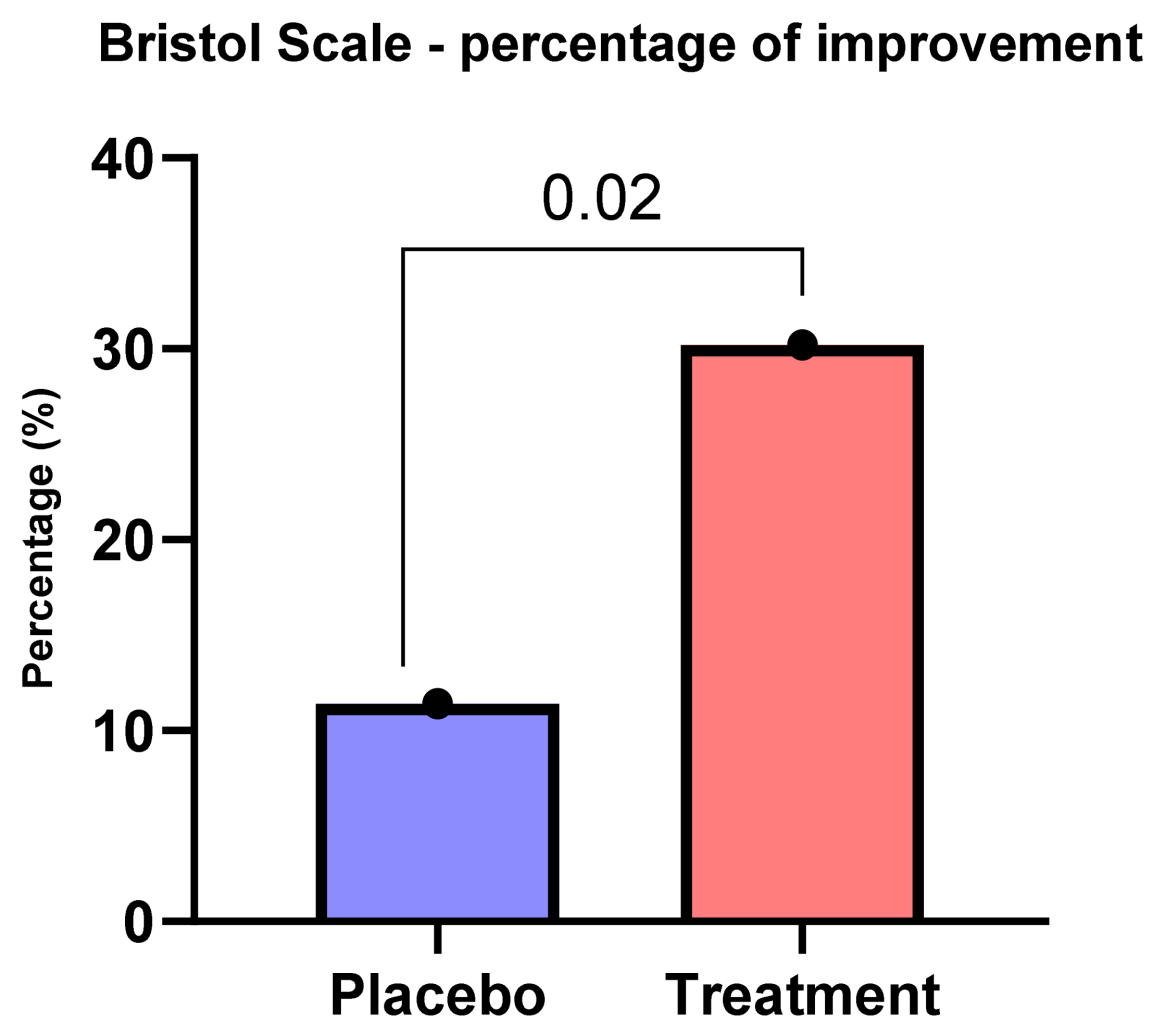
Percentage of participants showing movement toward type 4 on the Bristol Stool Scale during the placebo and treatment interventions. Improvement was defined bidirectionally as a change toward type 4 from a harder stool type below 4 or from a looser stool type above 4; it was not defined as a simple numerical increase in Bristol score. The treatment and placebo outcomes were compared using the χ^2^ test.

Using the post hoc bidirectional definition of improvement as movement toward type 4, Bristol score improvement occurred in 14/44 participants (31.8%) during treatment and in 5/44 (11.4%) during placebo (Figure 1).

### 3.3. Effect of ATECREM Serius on Gut Microbiota

A total of 315 ASVs were found among all samples. Specifically, TRT0 reported 297 ASVs, TRT1 287, PLT0 212, and PLT1 186. The number of ASVs shared by all four groups was 164, whereas the number of ASVs shared between two or three different groups is reported in Supplementary Figure S2.

The effect of the yeast-based treatment (TR) on gut microbiota was initially evaluated in terms of α-diversity. Chao, Shannon, ACE, Simpson, and Fisher indexes were calculated by comparing paired samples of baseline (PLT0 and TRT0) and follow-ups (PLT1 and TRT1). No differences were observed for any of the five indexes, except for the comparison of TRT1 vs. PLT1, which, based on Chao, ACE, and Fisher indexes, showed a significant increase in α-diversity as a result of yeast-based treatment (FDR = 0.011, 0.012, and 0.049, respectively; Supplementary Figure S3). Thus, the effect of the 2-week wash-out (WO) was additionally verified by comparing those samples collected after the run-in (RI) against those collected after the WO, and, as reported in Supplementary Material (Figures S4 and S5), both the Volcano plot and the Principal Component Analysis (PCA) showed an optimal efficiency of the 2-week WO according to the absence of differences in gut microbiota profiles. In the absence of significant differences between PLT0 and TRT0, all these samples, identifying the baseline gut microbiota fingerprint before proceeding with the interventions, were encompassed into a unique group and named as T0.

**Figure 2:**
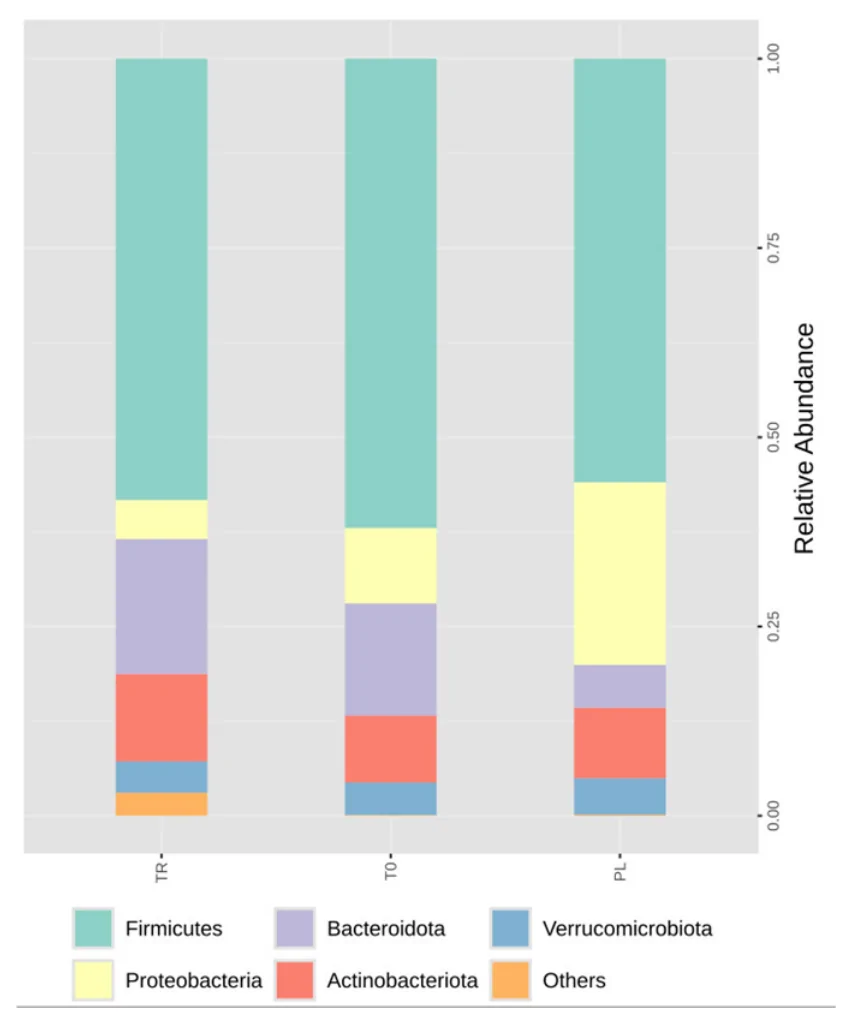
Mean values of the gut microbiota relative abundance (%) at the phylum level in volunteers at baseline (T0) and after 2 months of ATECREM Serius (TR) or placebo (PL) treatment.

The gut microbiota was also studied in volunteers after 2 months of the ATECREM Serius treatment (TR) or placebo (PL), as well as compared against their baseline fingerprint (T0). The β-diversity was studied according to PERMANOVA (corrected for multiple comparisons by the Benjamini–Hochberg test) between T0, TR, and PL samples, and, once again, the significance threshold was reached only for the pair TRT1 vs. PLT1 (FDR = 0.039). At the phylum level, Firmicutes was found to be the most representative taxon of the gut polymicrobial community, reporting values of relative abundance higher than 50% in all three groups (Figure 2). Compared to T0 (62%), Firmicutes showed a slight but not significant decrease in both the treated arms (TR: 58.3%; PL: 56%). Moreover, no differences involved Actinobacteriota, despite the higher relative abundance observed in both TR (11.6%) and PL (9.3%) compared to T0 (8.8%), or Verrucomicrobiota (T0: 4.3%, TR: 4.1%, PL: 4.8%). Compared with T0 (14.8%), Bacteroidota significantly decreased in PL (5.7%), but not in TR (17.8%). Conversely, compared to T0 (9.9%), Proteobacteria increased significantly in PL (24.1%), but not in TR (5.1%). Notably, an increased relative abundance of the group flagged as “Others”, encompassing phyla outside the top five, was observed in TR (3%) compared to T0 (<0.01%) and PL (<0.01%).

Hence, differences were then searched at deeper taxonomic levels. Within the top 10 families (Supplementary Figure S6), the scenario aligned with the taxonomic phylum level. *Lachnospiraceae* and *Ruminococcaceae* were the most abundant taxa, and together with *Akkermansiaceae,* no significant differences emerged. *Bacteroidaceae* were 7.9% at T0, slightly increased in TR (10.4%), and significantly decreased in PL (1.96%). PL led to more than a 2-fold increase in *Enterobacteriaceae* (from 8.1% at baseline to 21.5%), whereas this family decreased in TR (3.8%). Similarly, *Peptostreptococcaceae* increased in PL from 2.9% at T0 to 5.5% at the follow-up. Searching for differences in TR, a significant increase in Bifidobacteriaceae from 5.1% at T0 to 8.0% was recorded. By contrast, *Bifidobacteriaceae* decreased in PL to 3.8%. Minor and not significant changes involved the remaining families included in the top 10 (i.e., *Clostridia UCG014*, *Coriobacteriaceae*, and *Rikenellaceae*).

**Figure 3:**
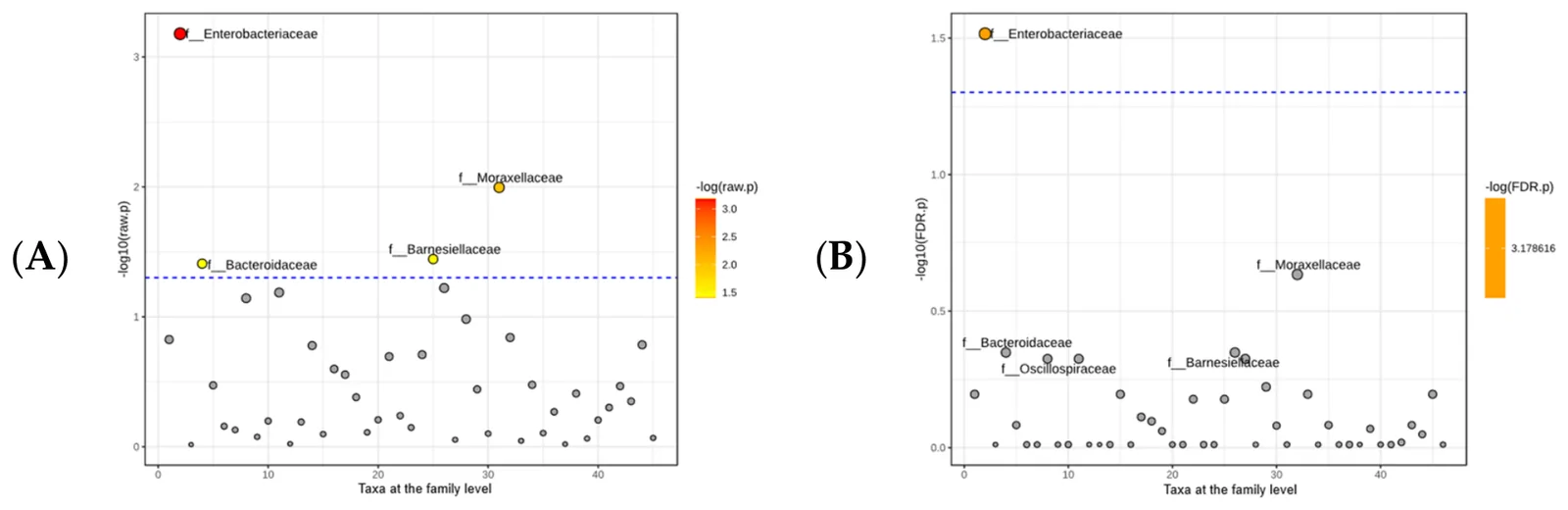
Significant differences at the family level, (**A**) as raw *p*-values and (**B**) after correction, between PLT1 and TRT1.

According to the crossover nature of the trial and the absence of differences in both baseline groups (PLT0 and TRT0), differences between both follow-ups (PLT1 and TRT1) were examined. These were found for *Enterobacteriaceae*, *Bacteroidaceae*, *Barnesiellaceae*, and *Moraxellaceae*, which were all increased in TRT1, except for *Enterobacteriaceae*, which, instead, were higher in PL than TR (Figure 3A). However, after statistical correction, significance was confirmed for *Enterobacteriaceae* only (Figure 3B).

**Figure 4:**
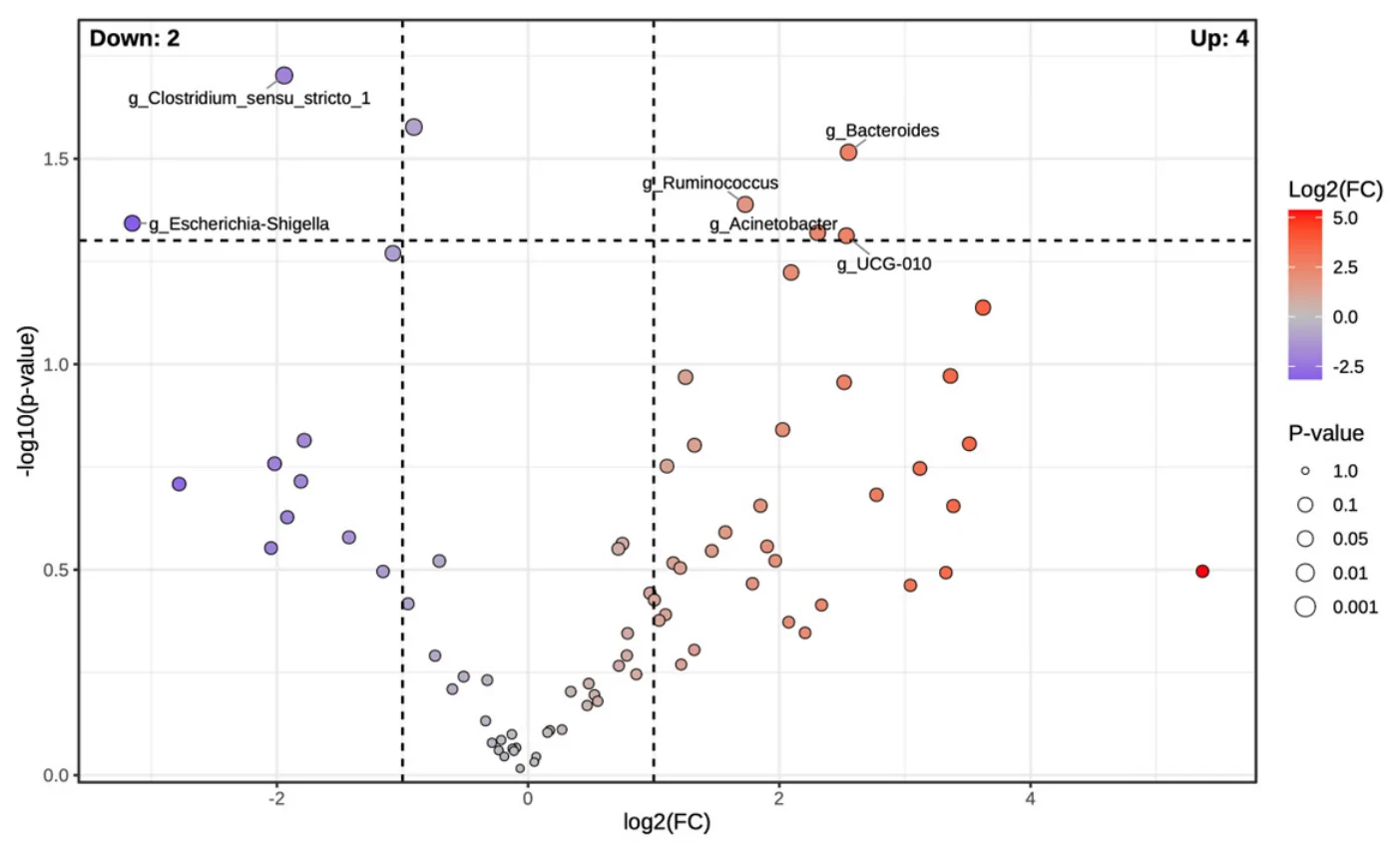
According to fold-change (FC) > |2| and *p*-value < 0.05, significant differences at the genus level between PLT1 (in blue, as “down”) and TRT1 (in red, as “up”).

At the genus level (Supplementary Figure S7), *Blautia* was found to be the genus most representative of *Lachnospiraceae*. *Akkermansia*, *Bacteroides*, *Escherichia*-*Shigella* and *Bifidobacterium* showed the same trends described at the family level. Compared to T0 (1.5%), *Clostridium sensu stricto* (clostridial cluster I) was significantly increased in PL (2.8%), whereas it did not change in TR (1.4%). Minor but not significant changes involved other genera included in the top 15. Thus, differences were searched at the genus level in PLT1 vs. TRT1. Six different genera reached the significance threshold according to a concomitant fold-change (FC) > |2| and *p*-value < 0.05. Specifically, these were *Clostridium sensu stricto* (clostridial cluster I), *Escherichia-Shigella*, *Ruminococcus*, *Acinetobacter*, *UCG-010*, and *Bacteroides* (Figure 4). These differences emerged due to a significantly higher abundance of *Clostridium sensu stricto* and *Escherichia-Shigella* in PLT1, while the remaining four genera mentioned above were higher in TRT1.

**Figure 5:**
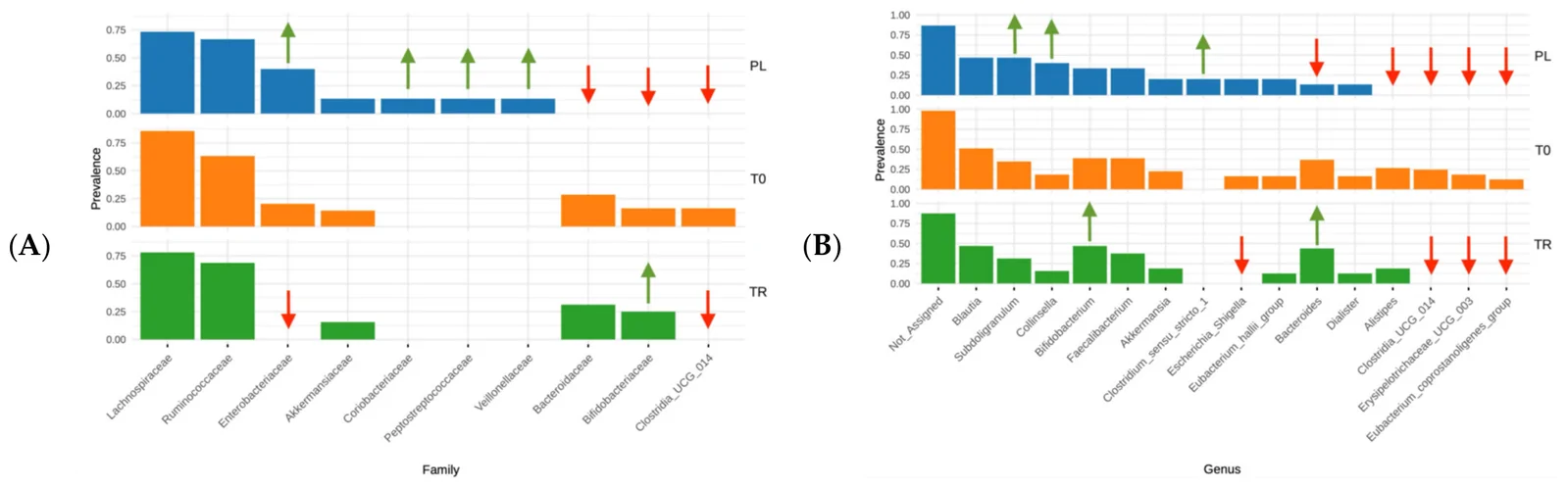
Core microbiota composition at the family (**A**) and genus (**B**) levels in volunteers at baseline (T0) and after 2 months of ATECREM Serius (TR) or placebo (PL) treatment. Core taxa were defined based on their prevalence in volunteers using a threshold of relative abundance ≥10% at the family level and ≥5% at the genus level. On stacked bars, red and green arrows indicate taxa showing decreased or increased prevalence compared with T0, respectively.

The volunteer core gut microbiota was inspected at the family and genus levels according to placebo/yeast treatment. By studying microbiota families filtered based on a relative abundance ≥10% and comparing results to T0, PL led to an increased fecal prevalence of *Enterobacteriaceae*, *Coriobacteriaceae*, *Peptostreptococcaceae*, and *Veillonellaceae*, decreasing the prevalence of *Bacteroidaceae* and *Bifidobacteriaceae*. Conversely, compared to T0, TR led to a decreased prevalence of *Enterobacteriaceae*, increasing that of *Bifidobacteriaceae* (Figure 5A). Both treatments (PL and TR) decreased the prevalence of *Clostridia UCG014*. A similar approach was carried out at the genus level while lowering the threshold of the relative abundance to ≥5% (Figure 5B). Both treatments (PL and TR) decreased the prevalence of *Clostridia UCG014*, *Erysipelotrichaceae UCG003*, and *Eubacterium coprostanoligenes group*. The TR confirmed its contribution to increasing the prevalence of *Bacteroides* and *Bifidobacterium* in volunteers while decreasing *Escherichia-Shigella*. By contrast, PL led to an increased prevalence of *Subdoligranulum*, *Collinsella*, and *Clostridium sensu stricto* (clostridial cluster I) while decreasing *Bacteroides* and *Alistipes*.

### 3.4. Effect of ATECREM Serius on Gut Microbiota According to Multivariate Statistics

**Figure 6:**
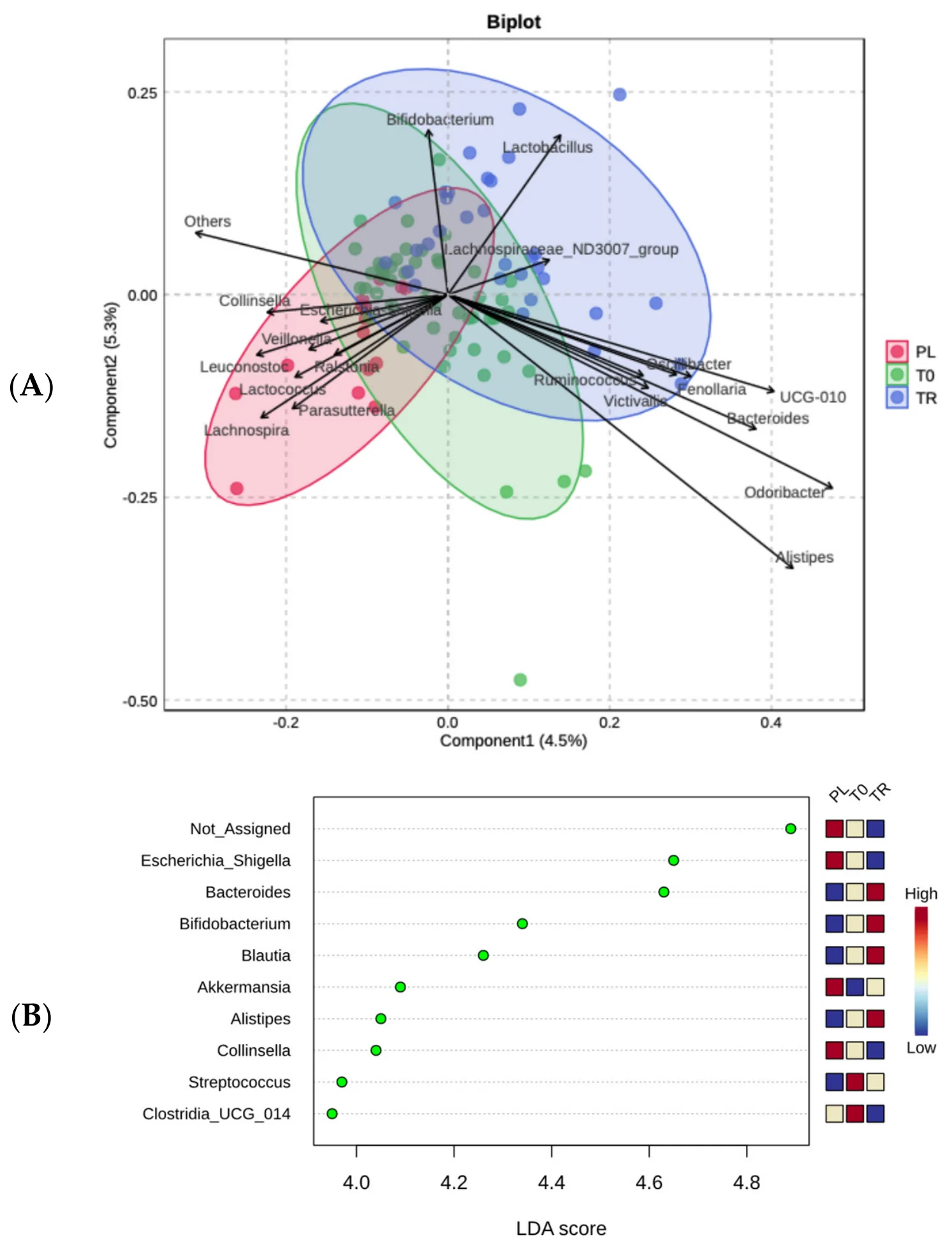
(**A**) Partial Least Squares Discriminant Analysis (PLS-DA) of the top 20 genera in volunteers at baseline (T0) and after 2 months of ATECREM Serius (TR) or placebo (PL) treatment. (**B**) Linear Discriminant Analysis Effect Size of the top 10 genera in volunteers at baseline (T0) and after 2 months of ATECREM Serius (TR) or placebo (PL) treatment.

The effect of ATECREM Serius on the gut microbiota was further investigated using Partial Least Squares Discriminant Analysis (PLS-DA). The 20 most abundant genera were selected and, after relative abundance normalization, projected onto a two-dimensional PLS-DA model, including both score (samples) and loading plots (Z-scores of genus relative abundances). As shown in the resulting output (Figure 6), the cluster of T0 samples was allocated in the central region of the PLS-DA space, whereas the TR and PL clusters exhibited opposite distribution patterns. Specifically, TR samples showed an increasing trend along both the first and the second Components, whereas PL samples were characterized by decreasing values along the same Components.

According to a critical evaluation of the sample rearrangement, although supported by discrete Component percentages, it was worth noting that samples included in the TR cloud can be split into two distinct subgroups. One TR subgroup mainly displayed increased Z-scores of *Bifidobacterium* and *Lactobacillus*, while the other showed elevated Z-scores of a cluster encompassing eight different genera, showing a marked association with positive scores of the first Component, with *Alistipes*, *Odoribacter*, and *Bacteroides* being the most prominent contributors. By contrast, projected according to negative values of the first Component, PL samples exhibited high Z-scores of *Lachnospira*, *Parasutterella*, *Lactococcus*, *Leuconostoc*, *Ralstonia*, *Veillonella*, *Collinsella*, and *Escherichia–Shigella*.

LEfSe analysis identified the top 10 bacterial taxa that discriminated among baseline (T0), placebo (PL), and yeast-treatment (TR) follow-ups (Figure 6B). Baseline samples were characterized by comparatively higher abundances of *Streptococcus* and *Clostridia_UCG-014*. The TR group was characterized by higher relative abundances of *Bifidobacterium*, *Blautia*, *Alistipes*, and *Bacteroides*. In contrast, *Escherichia–Shigella* and *Collinsella* were more abundant in the PL group and showed lower levels following the yeast-based treatment. *Akkermansia* was also associated mainly with the PL group, although its abundance in TR appeared intermediate between PL and T0. The same LEfSe analysis was also carried out comparing specifically TRT1 vs. PLT1 at the family and genus levels (Supplementary Figure S8A and S8B, respectively) and showed the discriminatory involvement between groups of the above-mentioned taxa.

**Figure 7:**
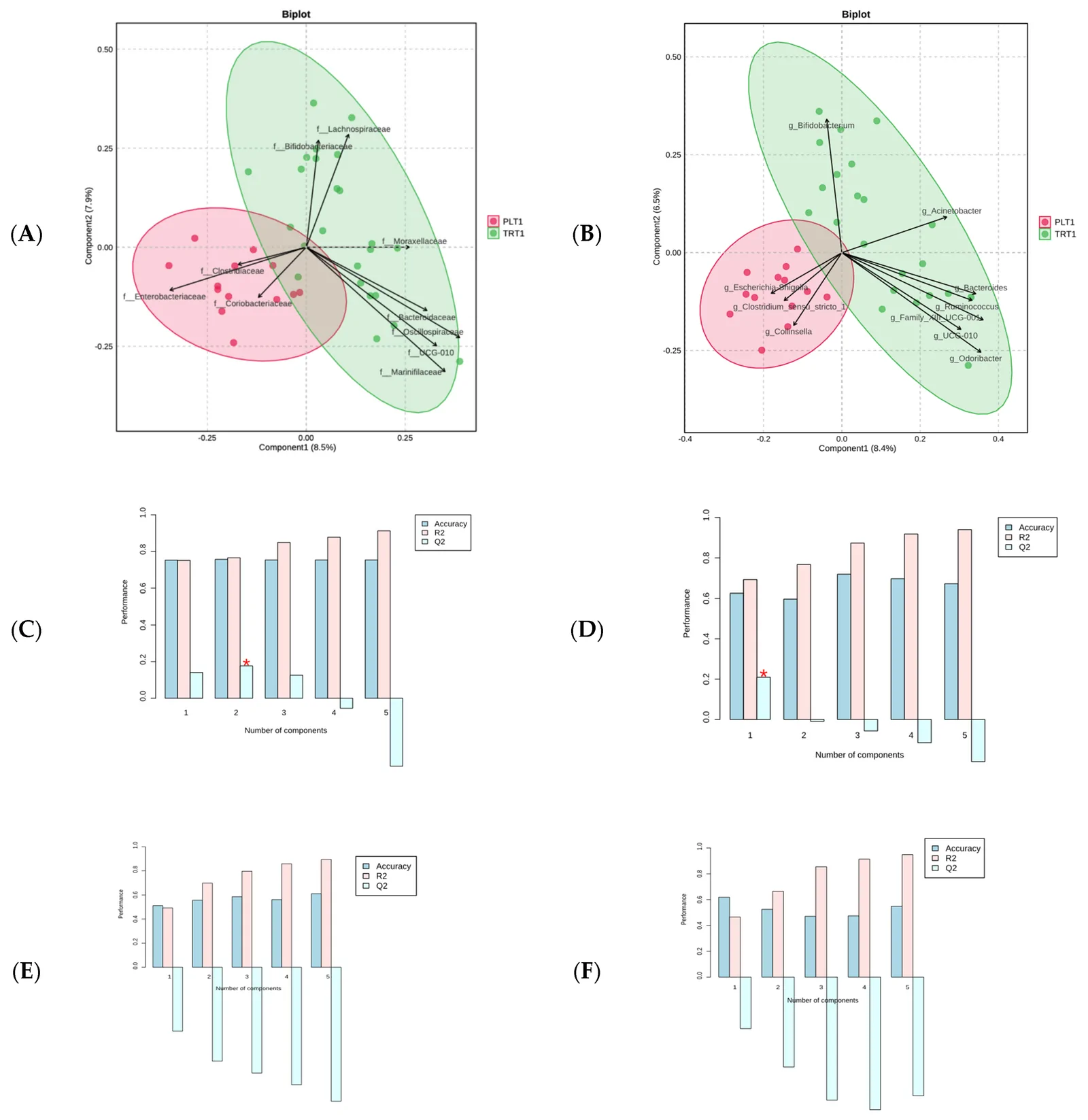
Partial Least Squares Discriminant Analysis (PLS-DA) score plot (**A**) at the family and (**B**) genus levels in volunteers after 2 months (T1) of placebo (PL) and ATECREM Serius (TR) treatment. PLS-DA performance during the analysis of T1 samples at the family and genus levels ((**C**) and (**D**), respectively) and related baseline samples (T0) at the family and genus levels ((**E**) and (**F**), respectively). In panels (**C**,**D**), the asterisk showed the highest Q^2^ value between 1–5 components.

To validate the results with a focus on the performance of the model, the same analytical approach was carried out on baseline (T0) and follow-up (T1) samples, which were studied separately to avoid bias derived from overfitting. The biplots at the family and genus levels reported a similar sample rearrangement onto the two-dimensional factor-plane (Figure 7A and 7B, respectively). *Enterobacteriaceae* (i.e., *Escherichia-Shigella*), *Clostridiaceae* (i.e., *Clostridium sensu stricto*), and *Coriobacteriaceae* (i.e., *Collinsella*) were the taxa most representative of the PLT1 samples. *Bifidobacteriaceae* (i.e., *Bifidobacterium*) and *Lachnospiraceae* (mainly supported by *Blautia* abundances, which was out of top 10 genera) were the taxa mostly representative of a subset of the TRT1 samples, whereas *Moraxellaceae* (i.e., *Acinetobacter*), *Bacteroidaceae* (i.e., *Bacteroides*), *UCG-010*, *Oscillospiraceae*, *Ruminococcus*, *Odoribacter*, and *Family XIII-UCG-001* were the taxa mostly representative of the other subset of TRT1 samples.

Concerning the performance of the PLS-DA model, this was evaluated by considering an increasing number of latent components (1–5) through cross-validation (Figure 7C–E). In T1 samples (Figure 7C,D), the classification accuracy was highest with 1–2 components, with values between 60 and 80%. Similarly, the coefficient of determination (R^2^) increased slightly from 70 to 75% with the first two components and stabilized at approximately 90–95% with 3–5 components. In contrast, the predictive parameter Q^2^ reached its highest value with 1–2 components (approximately 20%), which was therefore identified as the optimal model, and markedly decreased following the inclusion of additional components. Overall, these results suggest that adding further components does not improve the predictive ability of the model and may lead to overfitting.

By contrast, according to negative Q^2^ values, the performance of the PLS-DA was notably low in T0 samples at both the family (Figure 7E) and genus (Figure 7F) levels, suggesting an almost complete overlap of these groups due to overfitting phenomena. A permutation test was run to verify these tendencies, and, according to 100 permutations, it clearly emerged that the tendency to differentiate TRT1 vs. PLT1 (*p* = 0.33; Supplementary Figure S9A) was greater than for TRT0 vs. PLT0 (*p* = 0.93; Supplementary Figure S9B). This was further confirmed by running an Orthogonal Partial Least Squares Discriminant Analysis (OPLS-DA) that showed a lower T-score during baseline comparison (TRT0 vs. PLT0) than the score observed during TRT1 vs. PLT1 comparison (Supplementary Figures S10 and S11, respectively).

### 3.5. Cross-over-Adjusted Analysis of Gut Microbiota Abundance and Prevalence

**Figure 8:**
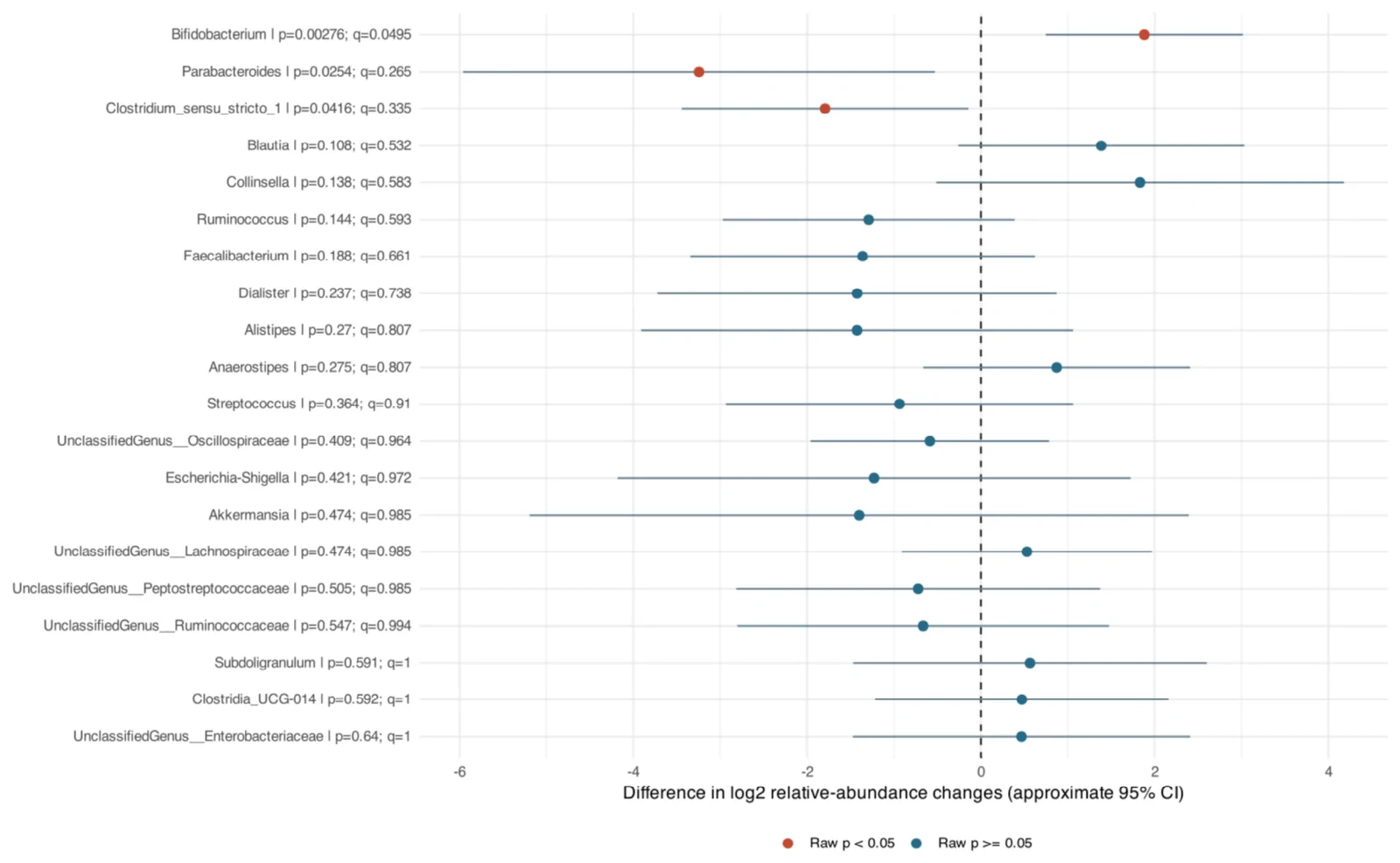
Forest plot showing the Treatment × Time interaction, representing the difference in pre-to-post changes in Log2-transformed relative abundance between the ATECREM Serius yeast treatment and placebo. Points indicate the estimated β-coefficients, and horizontal lines indicate the approximate 95% confidence intervals. Positive coefficients indicate a greater pre-to-post change (T0 to T1) during yeast treatment, whereas negative coefficients indicate a greater change during placebo. Taxon labels report the raw *p*-value and the corresponding Benjamini–Hochberg false discovery rate (FDR)-adjusted q-value.

Treatment-associated changes in gut microbiota abundance and prevalence were primarily assessed using the cross-over-adjusted MaAsLin3 mixed-effects analysis (Supplementary Table S1 and Figure S12). At the abundance level, *Bifidobacterium* was the only genus showing a significant Treatment × Time interaction after FDR correction (β = 1.88, *p* = 0.0028, q = 0.0495), indicating a greater pre-to-post increase during yeast treatment compared with placebo (Figure 8). This tendency was also evident from the paired relative abundances, although a marked inter-individual variability was observed (Supplementary Figure S13). A nominal treatment-related effect was also found for *Parabacteroides* and *Clostridium sensu stricto* 1 (*p* < 0.05), although significance was lost after correction for multiple comparisons. No significant abundance changes were found for the remaining genera. Overall, the paired profiles confirmed a marked inter-individual variability, particularly for *Blautia*, *Collinsella*, *Parabacteroides*, and *Ruminococcus* (Supplementary Figure S13).

Different results emerged for prevalence. Significant Treatment × Time interactions were found for *Collinsella*, *Subdoligranulum*, *Ruminococcus*, and *Erysipelotrichaceae UCG-003* (q < 0.05). The first three showed a lower pre-to-post change during yeast treatment compared with placebo, whereas the opposite pattern was found for *Erysipelotrichaceae UCGr-003* (Supplementary Figure S14). These findings mainly reflect differences in taxon occurrence rather than changes in their abundance among positive samples.

Notably, the effect observed for *Bifidobacterium* was specifically related to abundance rather than prevalence. Overall, these results support a yeast-associated increase in *Bifidobacterium*, together with additional treatment-related changes mainly involving the occurrence of selected bacterial genera.

### 3.6. Effect of ATECREM Serius on Gut Volatile Organic Compounds (VOCs)

The untargeted analysis of fecal metabolites (GC–MS) allowed for the identification of a total of 134 volatile organic compounds (VOCs), grouped into the following chemical classes: alcohols (16), aldehydes (10), esters and methyl esters (24), hydrocarbons (7), indoles (3), ketones (10), organic acids (18), phenols (4), sulfur-containing compounds (4), terpenes (24), and others (14).

The percentage distribution of each chemical class was examined by comparing the two treatment groups at the beginning and at the end of both administrations. Any statistically significant differences were observed in terms of chemical class. Phenols, indoles, and organic acids were the most represented classes across all the examined groups. In particular, the highest percentage of organic acids (29.73%) was detected in samples after treatment with *S. uvarum* SERIUS.

**Figure 9:**
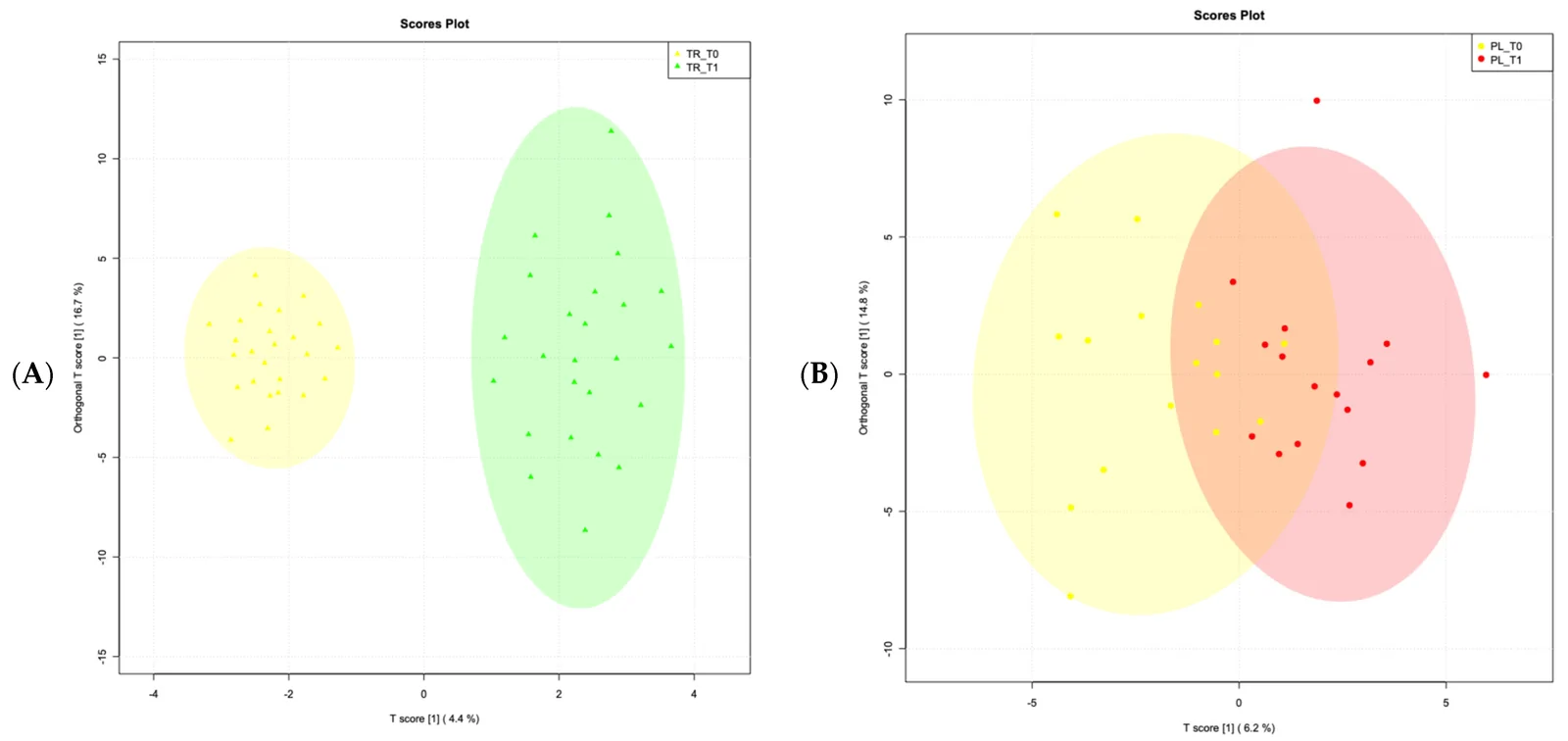

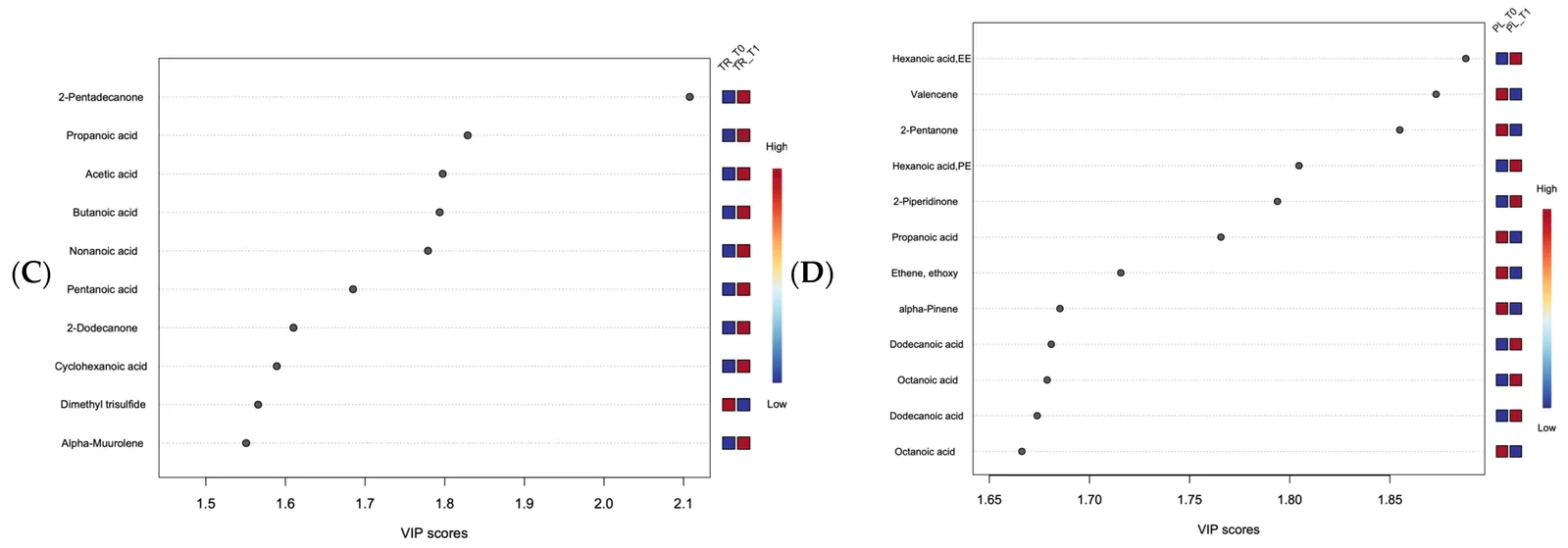
Orthogonal Partial Least Squares Discriminant Analysis (OPLS-DA) of fecal volatile organic compounds (VOCs) in volunteers before (T0) and after a 2-month treatment (T1). (**A**) Score plot of yeast-treated (ATECREM Serius containing *S. uvarum* SERIUS) and (**B**) placebo-treated subjects. Loading plot showing the top 10 metabolites ranked by variable importance in projection (VIP score) in the (**C**) yeast and (**D**) placebo arm.

Single metabolite concentrations were examined by running an Orthogonal Partial Least Squares Discriminant Analysis (OPLS-DA) according to normalized values before and after the treatment with yeast or placebo. The resulting score plots revealed a clear sample segregation following the yeast treatment (Figure 9A). In contrast, this separation was less pronounced after the placebo treatment, as indicated by the partial overlap of the T0 and T1 clusters (Figure 9B). This trend was further confirmed by loading plots, which reported, among the top 10 metabolites, nine VOCs enriched at T1, with only a decrease in the concentration of dimethyl trisulfide (Figure 9C). A deeper analysis of these results revealed that the yeast treatment was mainly characterized by an enrichment of organic acids, particularly short-chain fatty acids (SCFAs), as indicated by their high Z-scores. Instead, the top 10 VOCs discriminating T0 vs. T1 in PL showed a more heterogeneous metabolite profile across different chemical classes, with six and four compounds enriched and reduced at T1, respectively (Figure 9D). Moreover, the unique SCFA included in the top 10 (i.e., propanoic acid) was reduced at T1.

### 3.7. Effect of ATECREM Serius on Fecal Free Amino Acids (FAAs)

An essential AA fold change resulted from fecal free amino acid (FAA) concentration: phenylalanine (Phe), isoleucine (Ile), histidine (His), leucine (Leu), lysine (Lys), methionine (Met), threonine (Thr), tryptophan (Trp), and valine (Val). In addition, other AAs and AA derivatives (γ-aminobutyric acid—GABA) were also assessed for their concentrations.

**Figure 10:**
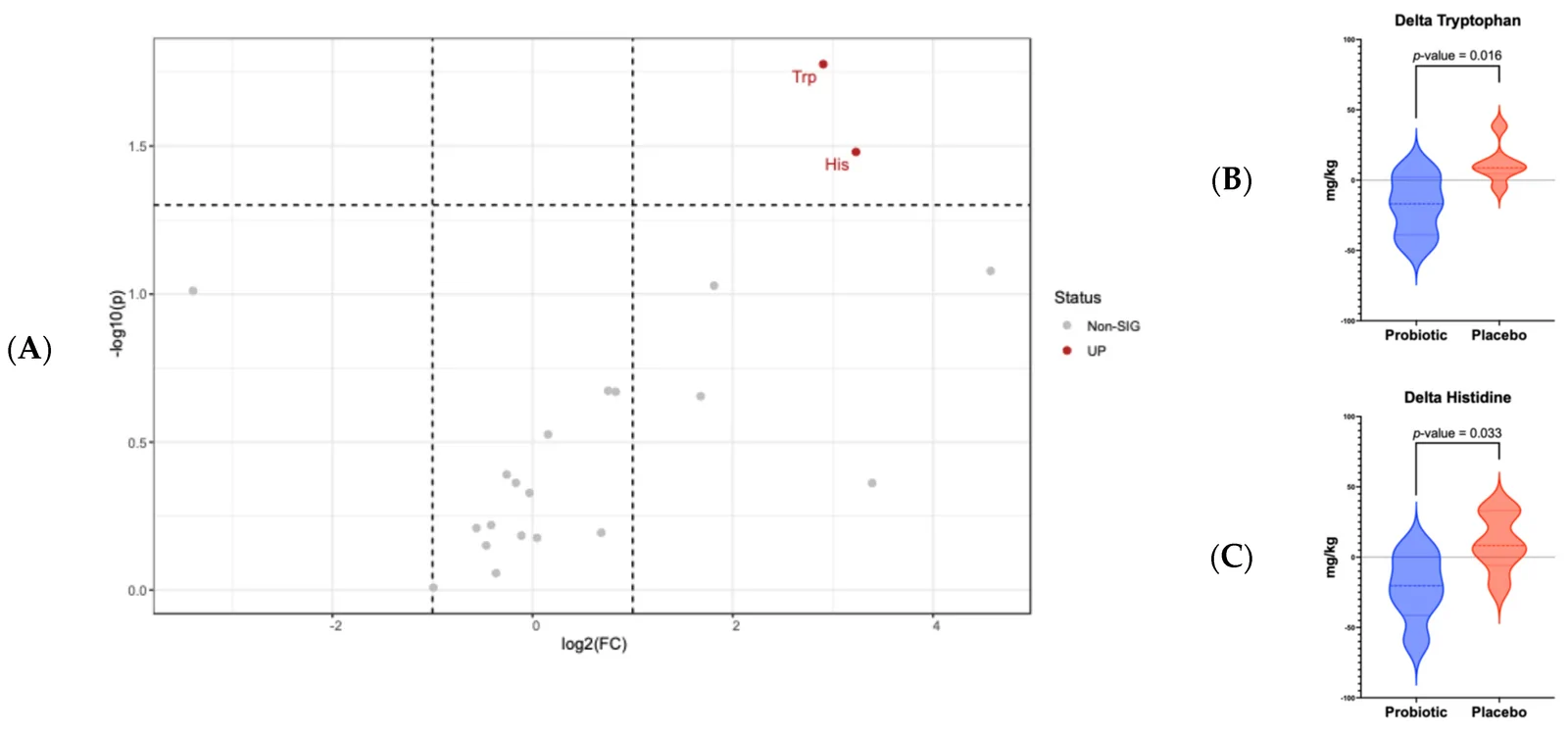
(**A**) Volcano plot of the delta values (Δ: T0–T1) of fecal free amino acids (FAAs) in volunteers treated with yeast or placebo. FAAs that significantly increased in PL are highlighted in red. Violin plots with *p*-values resulting from the comparison of delta values, expressed as median (centered dotted line) with IQR (25th –75th percentile, bottom and top dotten lines, respectively) of (**B**) Trp and (**C**) His found in feces after the yeast (blue) and placebo (red) treatment.

To highlight differences between yeast and placebo treatment, a comparative analysis was performed for each fecal compound detected by evaluating the difference between T0 and T1 concentrations, expressed as delta (Δ: T0–T1). For this purpose, a Volcano plot was used to report significant FAAs based on both biological and/or statistical significance (Figure 10A). This comparison revealed that Trp (Figure 10B) and His (Figure 10C) significantly decreased in samples from subjects treated with the yeast compared to the placebo.

### 3.8. Effect of ATECREM Serius on Urinary Metabolome

Untargeted GC–MS analysis of urine samples allowed the identification of a total of 79 VOCs, which were grouped into the following chemical classes: alcohols (2), aldehydes (7), organic acids (7), furans (6), hydrocarbons (13), ketones (11), phenols (5), sulfur-containing compounds (3), terpenes (10), and others (18). An initial comparative analysis of the relative percentage of each chemical class was performed by comparing the two treatment groups at baseline (T0) and after intervention (T1). No significant differences were observed. However, this analysis enabled the identification of the most abundant chemical classes in terms of percentage, resulting in carboxylic acids, phenols, and terpenes as the most represented classes across all groups examined.

**Figure 11:**
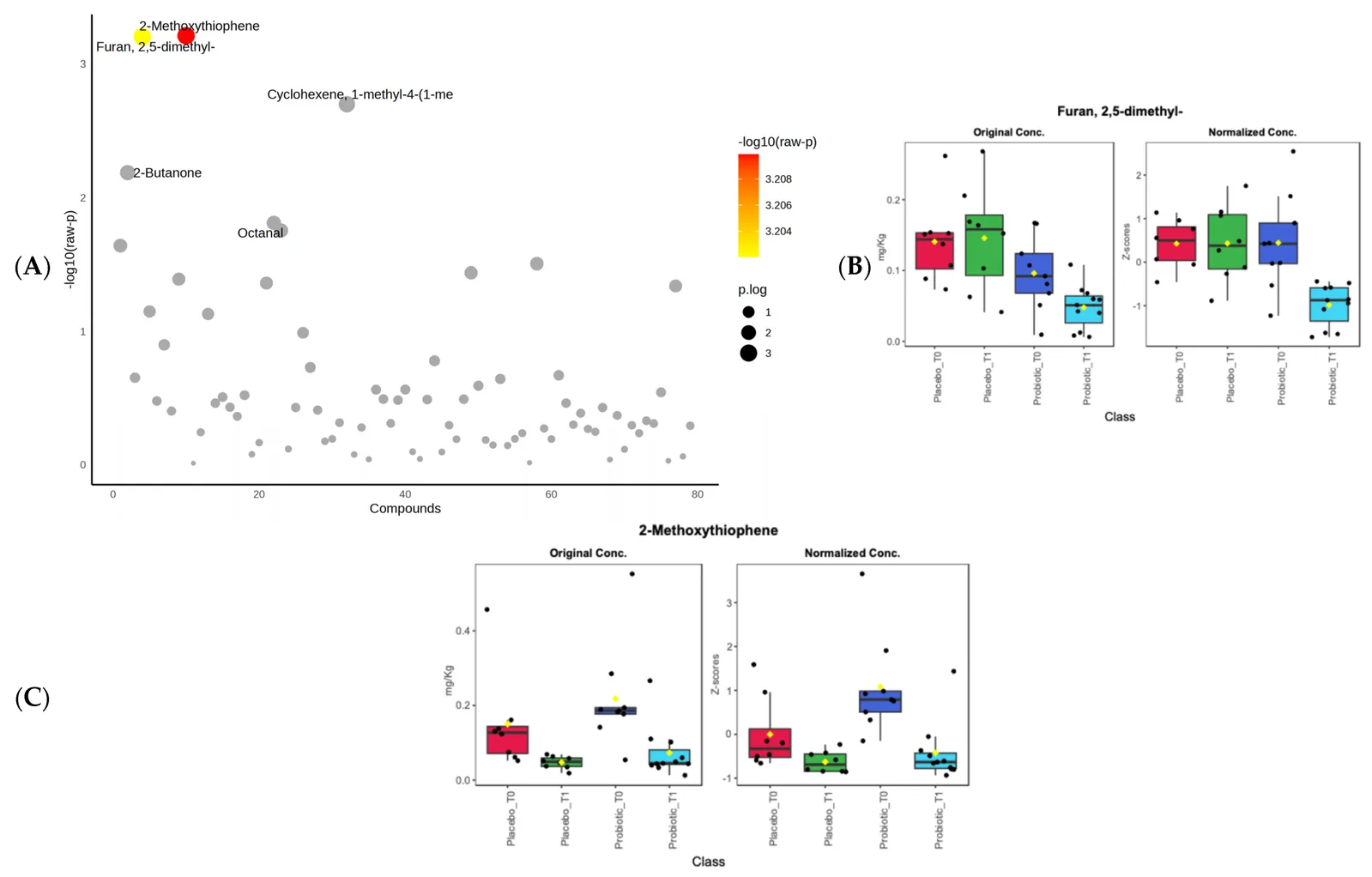
Urinary volatile organic compounds (VOCs). (**A**) ANOVA (followed by Tukey’s post hoc test) identifying metabolites that are significantly (*p*-value < 0.05) different between yeast and placebo groups. Box plots (mean, median, and IQR) of (**B**) 2,5-dimethylfuran and (**C**) 2-methoxythiophene concentrations at baseline (T0) and after (T1) the yeast and placebo treatment.

To assess statistically significant differences in single compounds, a comparative analysis (ANOVA with Welch’s *t*-test) between groups was performed. This revealed a significant difference in both 2,5-dimethylfuran and 2-methoxythiphene among arms (Figure 11A). However, upon deeper inspection, because of its statistically significant reduction in the yeast arm at T1, only the 2,5-dimethylfuran should be considered (Figure 11B). By contrast, the significance reached by 2-methoxythiophene, showing the lowest concentration in both the yeast and placebo arms at T1, can be explained by the baseline (T0) values of the yeast arm, where it was found at the highest concentration (Figure 11C).

**Figure 12:**
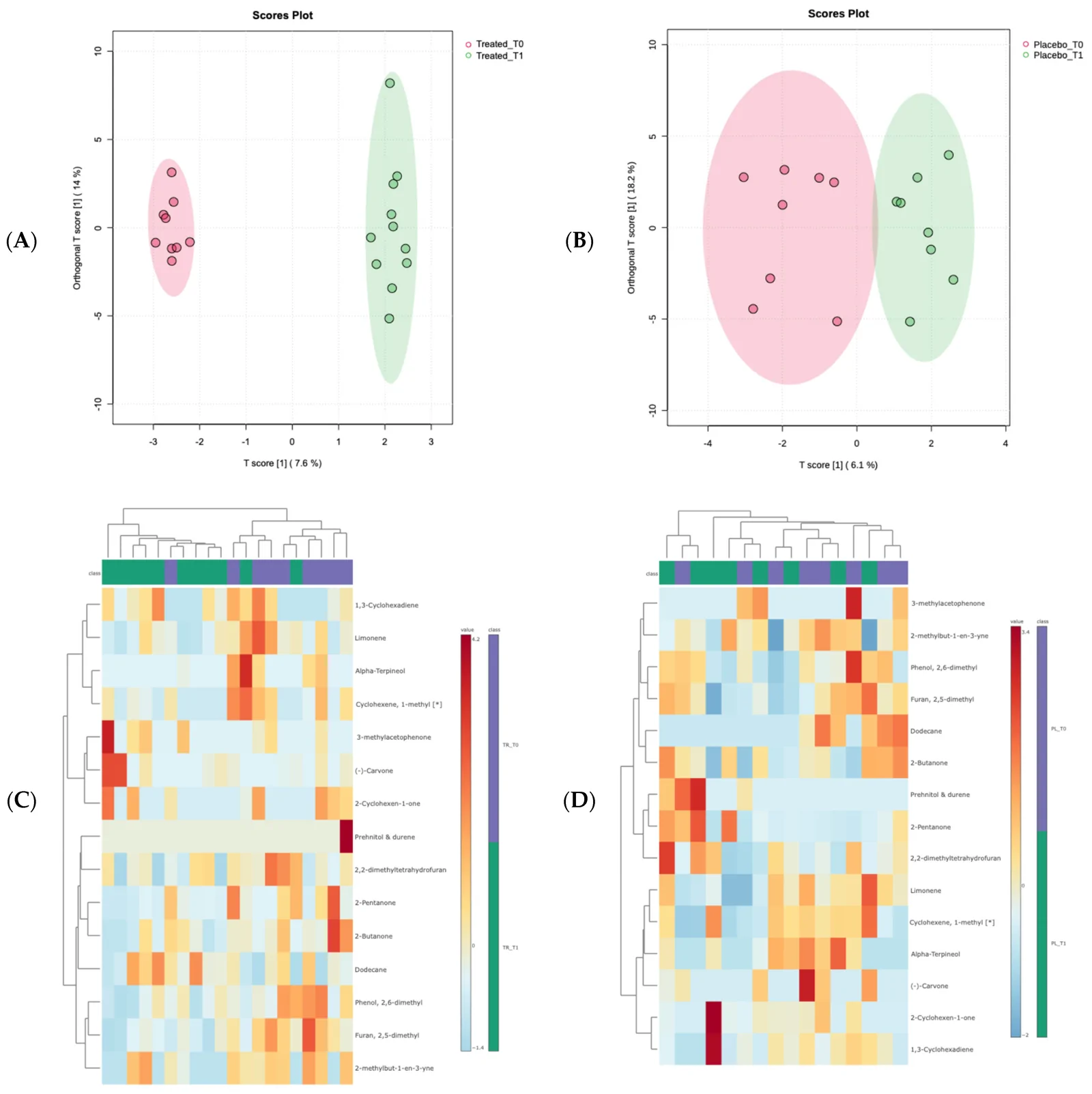
Multivariate analysis of urinary metabolome profiles in volunteers before (T0) and after 2 months (T1) of intervention. OPLS-DA score plot of (**A**) yeast-treated and (**B**) placebo-treated subjects. Heatmap with clustering based on the top 20 metabolites ranked by VIP score in the (**C**) yeast and (**D**) placebo arm. [*] Abbreviation of Cyclohexene, 1-methyl-4-(1-methylethylidene)-.

As a complementary approach, a multivariate statistical comparison was run. The OPLS-DA was able to distinguish T0 vs. T1 samples of the yeast arm better than those collected from volunteers who underwent the placebo intake (Figure 12A and 12B, respectively). Even T-score—describing the weighted metric distance between T0 and T1 samples—was higher in the yeast than the placebo arm (7.6% and 6.1%, respectively). Hence, according to VIP scores, using the top 20 ranked metabolites, a discrete clustering was recorded only in subjects who underwent the yeast treatment (Figure 12C). The Random Forest machine learning (RF-ML) analysis further confirmed this optimal clustering for yeast-treated samples, reporting a class error equal to 15% during T0 vs. T1 comparison (Supplementary Figure S15). Instead, in line with the heatmap of placebo samples, where no sample differentiation between T0 and T1 was found (Figure 12D), the RF-ML analysis showed a class error of 93.8% during T0 vs. T1 comparison (Supplementary Figure S16).

## 4. Discussion

In this randomized, single-blind, crossover pilot study, supplementation with the yeast *Saccharomyces uvarum* SERIUS (ATECREM Serius) did not induce changes in overall gastrointestinal symptom scores or in bowel habit parameters. Nevertheless, the intervention demonstrated a more subtle functional effect on intestinal physiology, as reflected by a significantly higher proportion of participants showing improvement in stool consistency (Bristol Stool Scale) in the yeast arm compared with placebo. This finding suggests a greater overall improvement in stool consistency with the yeast treatment, and further evidence points to an overall beneficial effect, e.g., in subjects with chronic constipation. The stability of baseline gastrointestinal status, together with the low prevalence of symptoms in this cohort, likely limited the magnitude of detectable clinical changes. These results are consistent with the concept that yeast-based products may act primarily as modulators of gut homeostasis rather than agents producing overt symptomatic effects.

Modern dietary habits characterized by high consumption of high-fat, low-fiber foods have been associated with progressive alterations of the gut microbial ecology and metabolic functions [35,36], contributing to the rising incidence of inflammatory and metabolic disorders [37,38]. In this context, the identification of novel probiotics and probiotic-based solutions capable of supporting intestinal homeostasis represents a crucial nutritional strategy [39,40,41,42]. The most common probiotics are strains of lactobacilli and bifidobacteria [43,44,45], although additional studies have assessed specific probiotic properties in additional lactic acid bacteria [46,47,48,49,50]. These probiotic strains directly compete with pathogens through the release of antibacterial organic acids [51,52], hydrogen peroxide [53], and bacteriocins [54,55,56,57]. Moreover, these microbes support the host’s well-being by synthesizing bioactive molecules supporting the optimal physiological functionality of different organs apart from the gut, including the brain [58], heart [59], liver [60] and kidney [61]. Certain yeasts like *Saccharomyces* species, in particular *S. cerevisiae* var. *boulardii*, were claimed to be probiotics [17]. Among yeasts, we previously carried out a genomic and functional characterization of *S. uvarum* SERIUS, which revealed the presence of gene repertoires associated with cell wall architecture, glycan biosynthesis, and signaling pathways potentially linked to immunomodulatory activity [21]. In the same study, *S. uvarum* SERIUS demonstrated the ability to mitigate inflammatory and oxidative stimuli in human cellular models while showing no high-similarity antimicrobial resistance determinants and limited growth at 37 °C, all features reinforcing its favorable safety profile. Based on the previous promising outcomes, the present randomized crossover intervention study investigated the effects of supplementation with ATECREM Serius, containing the yeast strain *S. uvarum* SERIUS.

Downstream of the analyses, although the well-documented resilience of the gut microbiota, slight but significant differences were detected after the yeast-based treatment for three out of five of the indices used to study the gut microbiota α-diversity, a difference that was also supported by PERMANOVA analysis of β-diversity between PL and TR follow-ups [62,63,64]. However, deeper taxonomic inspection revealed targeted compositional shifts that suggest a slight but positive modulatory effect of *S. uvarum* SERIUS. The intake of ATECREM Serius for 2 months led to an increase in bifidobacteria, while preventing the overgrowth of *Enterobacteriaceae* within the core microbiota observed, instead, during the placebo phase. According to LEfSe analysis, the yeast-based treatment was associated with a distinct microbial profile, including enrichment of genera commonly linked to carbohydrate fermentation and short-chain fatty acid production, particularly *Bifidobacterium* and *Blautia*, together with a reduction in the *Escherichia–Shigella* abundance compared with PL. The increase in *Bifidobacterium* is of particular interest, given its recognized role in saccharolytic fermentation, short-chain fatty acid metabolism, and maintenance—via trophic mechanisms onto colonocytes—of an optimal epithelial barrier function [65]. Conversely, the expansion of *Enterobacteriaceae*, the family to which *Escherichia–Shigella* belongs, is often considered a feature of ecological imbalance when sustained over time [66]. Indeed, *Enterobacteriaceae* are considered a symbiotic family of the colonic environment, but, encompassing different strains possessing pathobiont traits, the maintenance of their density within limited ranges of abundance represents a strategy to mitigate the risk of disease onset [67].

The observation that *Enterobacteriaceae* expansion occurred during placebo administration but not during yeast supplementation suggests that *S. uvarum* SERIUS may contribute to preserving the resilience of beneficial taxa rather than inducing large-scale reassembling of the microbiota structure associated with the physiological fluctuations usually occurring in the gut of healthy subjects. Indeed, core microbiota inspection during the placebo phase revealed the presence of additional taxa *de novo*, such as *Peptostreptococcaceae* and *Clostridium sensu stricto*, for which the scientific consensus in gut health remains controversial [68,69,70]. This does not imply that placebo administration exerted detrimental clinical effects, as no adverse outcomes were recorded, but rather highlights the potential regulatory role of *S. uvarum* SERIUS in preventing—or at least limiting—the proliferation of less desirable microorganisms. In fact, although yeasts are not permanent colonizers of the intestine and the limited growth of *S. uvarum* SERIUS at 37 °C supports this notion [21], they can influence bacterial communities through cross-kingdom interactions. These may include competition for nutrients, modulation of luminal redox conditions, and indirect metabolic interactions favoring beneficial anaerobes and disadvantaging pathobionts (or pathogens).

The metabolomic data provided functional evidence for these taxonomic findings. While the overall reassembly of VOCs in chemical classes did not significantly differ between groups, multivariate analysis showed a notable separation between baseline and post-intervention samples in the yeast arm, while the same was not observed after the placebo phase. Notably, organic acids, particularly SCFAs, emerged among those metabolites that most significantly contributed to this separation. SCFAs, particularly acetate, butyrate and propionate, are pivotal metabolites of host–microbiota interaction, acting as energy substrates for colonocytes, regulators of the epithelial barrier integrity, and modulators of the immune and metabolic pathways [71]. The observed enrichment following the yeast supplementation suggested that *S. uvarum* SERIUS may indirectly enhance the saccharolytic fermentative activity through ecological support of SCFA-producing bacterial taxa. In contrast, the placebo was characterized by a more heterogeneous metabolic pattern, including a reduction in propanoic acid among discriminant metabolites, further highlighting a different metabolic trajectory driven by the treatment that subjects underwent.

The reduction in fecal Trp observed after yeast supplementation offers additional insight into substrate utilization by gut microbiota. Trp is a key substrate for microbial metabolism and is converted into multiple indole derivatives that participate in host–microbiota signaling [72]. These metabolites act as ligands of the aryl hydrocarbon receptor (AhR), a transcription factor involved in mucosal immune regulation and epithelial homeostasis. However, indole derivatives were not directly quantified in the present study. Therefore, the decrease in fecal Trp can only be interpreted as being compatible with increased microbial utilization of this amino acid and cannot be taken as direct evidence of enhanced production of bioactive indole metabolites or AhR activation. Previous studies have associated AhR signaling with reinforcement of mucosal defenses, IL-22 production, and maintenance of epithelial integrity [73], but these mechanisms cannot be directly attributed to *S. uvarum* SERIUS based on the present data. Targeted metabolomic assessment of Trp-derived metabolites, together with inflammatory and immune-related biomarkers, will therefore be required in future studies to substantiate this mechanistic hypothesis. Similarly, the decrease in fecal histidine may reflect altered microbial amino acid metabolism, although histidine-derived metabolites were not measured and the biological significance of this change remains uncertain.

Urinary metabolomic profiling further supports the presence of systemic metabolic modulation associated with the *S. uvarum* SERIUS intake. Multivariate analysis demonstrated clearer discrimination between baseline and post-treatment samples in the yeast arm, supported by machine-learning analysis, compared with placebo. Notably, 2,5-dimethylfuran significantly decreased exclusively after yeast supplementation. While the biological relevance of this compound requires further clarification, previous studies quantifying this metabolite in urine samples usually noted it as a potential biomarker of various diseases. Specifically, 2,5-dimethylfuran was widely recognized as a biomarker of smoking individuals [74,75]. Still, it was also identified at high concentrations in the urine of subjects suffering from alopecia areata [76] and as a metabolite showing genotoxic effects in the kidney and colon of mice [77], with a positive correlation also being reported with patients affected by kidney stones [78]. However, the interpretation of the significant decrease in urinary 2,5-dimethylfuran observed following *S. uvarum* SERIUS supplementation requires caution. Smoking status, environmental exposures, hydration status, and renal function, including glomerular filtration rate, were not systematically assessed in the present study, and all these factors may potentially influence urinary VOC profiles. Therefore, the observed change in 2,5-dimethylfuran cannot be unequivocally attributed to the yeast intervention and should be considered an exploratory finding requiring confirmation in specifically controlled studies. Nevertheless, the overall multivariate discrimination observed between baseline and post-intervention urinary metabolomic profiles in the yeast arm suggests that supplementation may be associated with changes in systemic metabolic signatures, although the biological and clinical significance of these changes remains to be established. Additional limitations should also be acknowledged. The study population consisted of adults without a previous diagnosis of gastrointestinal or chronic diseases associated with significant gut dysbiosis. This selection inherently limits the magnitude of detectable microbial shifts due to baseline ecological resilience. The sample size was not calculated a priori due to the exploratory nature of this study, which mainly aimed at the identification of good-to-high tolerability of the yeast supplementation. Thus, while appropriate for exploratory metabolomic and microbiota profiling, which were included as secondary outcomes, it may not allow detection of smaller effect sizes. Furthermore, although metabolomic data suggested enhanced microbial utilization of Trp, indole derivatives were not directly quantified, and inflammatory biomarkers were not assessed in parallel. Another limitation that should be addressed concerns the placebo that, containing maltodextrins and xanthan gum, matched the intervention matrix but cannot be assumed to be biologically inert. Xanthan gum can be utilized by specific members of the human gut microbiota and may promote the expansion of xanthan-degrading taxa and related cross-feeding networks [79]. Maltodextrin has also shown biological activity in experimental models, where it increased epithelial endoplasmic reticulum stress, reduced Mucin-2 expression, and enhanced susceptibility to intestinal inflammation [80].

Future investigations should include targeted quantification of Trp-derived metabolites and systemic inflammatory markers to better delineate mechanistic pathways. Studies conducted in populations characterized by metabolic dysfunction or low-grade inflammation may further clarify whether the stabilizing and metabolic effects observed here translate into clinically meaningful benefits allowing the potential use of the probiotic claimed for this yeast-based preparation. Additionally, longer intervention periods and dose–response evaluations, as well as the hypothetical use of different placebos such as cellulose, could help define the optimal conditions under which *S. uvarum* SERIUS exerts its functional potential.

## 5. Conclusions

The present study indicates that oral ATECREM Serius was well tolerated within the investigated population and intervention period.

Exploratory analyses identified treatment-associated differences in selected gut microbial and metabolic features. In particular, the cross-over-adjusted analysis showed a greater pre-to-post increase in *Bifidobacterium* abundance during *S. uvarum* SERIUS supplementation compared with placebo, together with treatment-associated differences in the prevalence of selected bacterial genera. Metabolomic analyses also indicated differences in fecal organic acids, free amino acids, and urinary volatile compounds between study conditions. Given that the study was conducted in subjects without chronic conditions previously associated with gut dysbiosis, the exploratory nature of the study, the limited sample size, and the absence of a priori power calculation for these secondary outcomes, such outcomes identify the most physiologically appropriate beneficial action aimed at preserving ecological balance rather than correcting overt dysbiosis. Therefore, larger and adequately powered trials with prespecified microbiota and metabolic endpoints are required to confirm these observations and to determine their biological and clinical relevance.

## Figures and Tables

**Table 1:** Baseline characteristics of the enrolled participants.

	Total (*n* = 44)	Placebo (*n* = 19)	Treatment (*n* = 25)	*p*-Value
Females, N (%)	23 (52.3%)	9 (47.4%)	14 (56.0%)	0.57
Age (years)	29.4 ± 1.11	28.5 ± 1.88	30.1 ± 1.36	0.40
BMI (kg/m^2^)	23.5 ± 0.49	22.5 ± 0.63	24.2 ± 0.69	0.09
**Comorbidities**				
Allergy, N (%)	1 (2.3%)	0 (0%)	1 (4.0%)	0.265
Hypertension, N (%)	1 (2.3%)	0 (0%)	1 (4.0%)	0.265
Thyroid nodules, N (%)	1 (2.3%)	0 (0%)	1 (4.0%)	0.265
Lactose intolerance, N (%)	1 (2.3%)	0 (0%)	1 (4.0%)	0.265
Thyroiditis, N (%)	2 (4.5%)	2 (10.5%)	0 (0%)	0.265
Anemia, N (%)	1 (2.3%)	1 (5.3%)	0 (0%)	0.265
**Gastrointestinal symptoms**				
Gastric fullness, N (%)	1 (2.3%)	0 (0%)	1 (4.0%)	0.378
Epigastric pain, N (%)	1 (2.3%)	0 (0%)	1 (4.0%)	0.378
Nausea/Vomiting, N (%)	0 (0%)	0 (0%)	0 (0%)	—
Heartburn, N (%)	1 (2.3%)	0 (0%)	1 (4.0%)	0.378
Abdominal bloating, N (%)	12 (27.3%)	4 (21.1%)	8 (32.0%)	0.419
Abdominal pain, N (%)	3 (6.8%)	0 (0%)	3 (12.0%)	0.118

Values are mean ± SEM for continuous variables and *n* (%) for categorical variables. *p*-values were calculated using Student’s *t*-test for continuous variables and Pearson’s χ^2^ test for categorical variables.

**Table 2:** Dietary intake assessed by 3-day food diaries at baseline (TRT0 and PLT0) and follow-ups (PLT1 and TRT1).

	Yeast-Based Treatment (TR; N = 44)			Placebo (PL; N = 44)		
	T0	T1	*p*	T0	T1	*p*
Energy (kcal/day)	1731.6 ± 103.9	1763.1 ± 93.5	0.88	1794.5 ± 86.7	1859.1 ± 92.4	0.67
CHOs (g/day)	176.7 ± 41.2	180.2 ± 25.3	0.90	185.2 ± 14.2	190.9 ± 20.1	0.64
Proteins (g/day)	78.8 ± 12.6	75.3 ± 13.8	0.60	76.3 ± 13.8	78.9 ± 13.1	0.78
Total fats (g/day)	60.1 ± 4.5	58.7 ± 6.0	0.44	57.9 ± 5.5	63.0 ± 5.9	0.31
Fibers (g/day)	14.7 ± 1.7	16.0 ± 1.6	0.62	14.9 ± 1.7	15.5 ± 2.0	0.78

Values are expressed as mean ± SEM. *p*-values refer to within-group comparisons between baseline and the end of the intervention (paired *t*-test). Abbreviations: CHOs, carbohydrates.

**Table 3:** Changes in gastrointestinal symptoms and bowel habits between baseline (T0) and follow-up (T1) in the placebo and treatment groups.

	Group	T0	T1	*p*-Value
Bloating VAS (mm)	Placebo	14.86 ± 4.80	8.81 ± 3.07	0.317
	Treatment	12.56 ± 3.41	16.51 ± 4.55	0.320
Abdominal pain VAS (mm)	Placebo	1.07 ± 2.86	0 ± 0	0.800
	Treatment	1.86 ± 1.64	1.53 ± 3.15	0.930
Bowel movements/week	Placebo	7.38 ± 0.50	8.00 ± 0.58	0.610
	Treatment	7.58 ± 0.53	9.02 ± 0.35	0.134

Values are expressed as mean ± SEM. *p*-values were calculated using the Wilcoxon signed-rank test for paired comparisons (T0 vs. T1) within each group.

**Table 4:** Distribution of Bristol Stool Scale scores at baseline (T0) and after intervention (T1) in placebo and treatment groups.

Bristol Stool Scale	Placebo T0 (*n* = 44)	Placebo T1 (*n* = 44)	Treatment T0 (*n* = 44)	Treatment T1 (*n* = 44)
2	2 (4.5%)	3 (6.8%)	2 (4.5%)	0 (0.0%)
3	12 (27.3%)	10 (22.7%)	7 (15.9%)	10 (25.0%)
4	25 (56.8%)	23 (52.3%)	26 (59.1%)	33 (75.0%)
5	4 (9.1%)	7 (15.9%)	7 (15.9%)	1 (2.3%)
6	1 (2.3%)	1 (2.3%)	2 (4.5%)	0 (0%)

Values are expressed as *n* (%). Differences in Bristol Stool Scale distribution between T0 and T1 within each group were assessed using the χ^2^ test.

## Data Availability

Data will be made available on request to the corresponding author.

## Supplementary Materials

The following supporting information can be downloaded at https://www.mdpi.com/article/10.3390/nu18203380/s1. Figure S1: CONSORT flow diagram of study participants showing participant recruitment, eligibility assessment, randomization, allocation to the two intervention sequences, follow-up throughout the crossover trial, withdrawals/exclusions, and participants included in the final analysis. Figure S2: Venn diagram showing the distribution of ASVs detected at baseline and after the yeast and placebo interventions (TRT0, TRT1, PLT0, and PLT1, respectively). Numbers indicate ASVs unique to each condition or shared among two, three, or all four groups. Figure S3: Gut microbiota α-diversity indices (Chao, Shannon, ACE, Simpson, and Fisher) across paired baseline (TRT0 and PLT0) and post-intervention samples (TRT1 and PLT1). Statistical significance was adjusted for multiple comparisons using the Benjamini–Hochberg false discovery rate (FDR). Figure S4: Based on ASV relative abundance, Volcano plot between run-in (RI) and wash-out (WO); Figure S5: Based on normalized values of ASV relative abundance, principal component analysis score-plot of run-in (RI) and wash-out (WO); Figure S6: Mean values of the gut microbiota relative abundance (%) at the family level in volunteers at baseline (T0) and after 2 months of ATECREM Serius (TR) or placebo (PL) treatment; Figure S7: Mean values of the gut microbiota relative abundance (%) at the genus level in volunteers at baseline (T0) and after 2 months of ATECREM Serius (TR) or placebo (PL) treatment. Figure S8: Linear discriminant analysis effect size (LEfSe) analysis comparing yeast and placebo follow-up samples (TRT1 and PLT1, respectively) at the (A) family and (B) genus levels. Taxa are ranked according to their linear discriminant analysis (LDA) effect size and their association with TRT1 or PLT1. Figure S9: Permutation analysis of PLS-DA model performance comparing (A) TRT1 versus PLT1 and (B) TRT0 versus PLT0. Model significance was assessed using 100 permutations. Figure S10: Orthogonal Partial Least Squares Discriminant Analysis (OPLS-DA) comparing microbiota profiles of baseline samples preceding the ATECREM Serius (TRT0) and placebo (PLT0) intervention. Figure S11: Orthogonal Partial Least Squares Discriminant Analysis (OPLS-DA) comparing microbiota profiles after 2 months of ATECREM Serius (TRT1) and placebo (PLT1). Figure S12: Forest plots show β coefficients and approximate 95% confidence intervals for the sequence effect (Yeast–Placebo) and the Treatment × Time interaction (Yeast:TimePost). Circles represent abundance models and triangles represent prevalence models; symbol shading reflects the corresponding FDR-adjusted *p*-values. The heatmaps summarize the direction and magnitude of β coefficients for the covariates included in the models, including period, Time × Period, treatment, sequencing depth, and time, separately for prevalence and abundance. Asterisks indicate covariate associations significant after FDR correction (q < 0.05; q < 0.005). Positive and negative β coefficients indicate associations in opposite directions relative to the corresponding reference category. Figure S13: Individual paired relative abundances (%) are shown for *Bifidobacterium*, *Blautia*, *Clostridium sensu stricto 1*, *Collinsella*, *Parabacteroides*, and *Ruminococcus*. Each line connects the pre- and post-intervention samples from the same participant within the placebo or yeast-treatment period. The plots provide a descriptive visualization of individual trajectories and highlight the marked inter-individual variability underlying the genus-level responses. Inferential treatment effects accounting for the crossover design were assessed separately using MaAsLin3. Figure S14: Forest plot showing the Treatment × Time interaction for prevalence, expressed as the difference in pre-to-post log-odds changes between the ATECREM Serius yeast treatment and placebo periods. Points represent estimated β coefficients and horizontal lines approximate 95% confidence intervals. Positive values indicate a greater pre-to-post change in prevalence during yeast treatment compared with placebo, whereas negative values indicate a greater change during placebo. Taxon labels report raw *p*-values and Benjamini–Hochberg FDR-adjusted q-values; red points denote raw *p* < 0.05 and blue points *p* ≥ 0.05. Figure S15: Random Forest machine learning of sample allocation prediction based on urinary volatile organic compound (VOC) relative concentrations assessed at baseline (T0) and after 2 months of ATECREM Serius treatment. Figure S16: Random Forest machine learning of sample allocation prediction based on urinary volatile organic compound (VOC) relative concentrations assessed at baseline (T0) and after 2 months of placebo treatment. Table S1: The table reports the bacterial feature, model covariate (metadata), contrast or covariate value, model-term name, estimated coefficient (coef), standard error (stderr), component-specific *p*-value and Benjamini–Hochberg FDR-adjusted q-value (pval_individual and qval_individual), MaAsLin3 joint-test *p*-value and FDR-adjusted q-value (pval_joint and qval_joint), model type (abundance or prevalence), total number of observations included (N), and number of non-zero observations (N_not_zero). Models accounted for treatment, time, period, sequence, Treatment × Time and Time × Period interactions, sequencing depth, and repeated observations from the same participant. The TreatmentYeast:TimePost coefficient represents the difference in pre-to-post change between the yeast-treatment and placebo periods. Associations were considered statistically significant at q < 0.05. The structure and statistical fields reported in the supplementary table are shown in the complete MaAsLin3 output.

## Abbreviations

The following abbreviations are used in this manuscript:

AhR	Aryl Hydrocarbon Receptor
ALT	Alanine Transaminase
ASV	Amplicon Sequence Variant
AST	Aspartate Transaminase
BCFAs	Branched-Chain Fatty Acids
BMI	Body Mass Index
DNA	Deoxyribonucleic Acid
FAAs	Free Amino Acids
GABA	γ-Aminobutyric Acid
GC-MS	Gas Chromatography Mass Spectrometry
GGT	Gamma-Glutamyl Transferase
GI	Gastrointestinal
HDL	High-Density Lipoprotein
IS	Internal Standard
LDL	Low-Density Lipoprotein
OPLS-DA	Orthogonal Partial Least Squares Discriminant Analysis
PCA	Principal Component Analysis
PERMANOVA	Permutational Multivariate Analysis of Variance
PL	Placebo
PLS-DA	Partial Least Squares Discriminant Analysis
QC	Quality Control
RI	Run-in
RF-ML	Random Forest Machine Learning
rRNA	Ribosomal Ribonucleic Acid
SCFAs	Short-Chain Fatty Acids
SIM	Selective Ion Monitoring
T0	Baseline Time-Point
T1	Post-Intervention Time-Point
TC	Total Cholesterol
TG	Triglycerides
TR	Treatment
UC	Ulcerative Colitis
VAS	Visual Analog Scale
VOCs	Volatile Organic Compounds
WO	Wash-out
