# Supplementary material for: Effects of a Saccharomyces uvarum Cell-Based Preparation on Gut Microbial Ecology and Metabolic Activity in Adults Without Diagnosed Gastrointestinal Disease: A Randomized Placebo-Controlled Crossover Pilot Study
Source: Nutrients. 2026 Oct 15;18(20):3380. doi: 10.3390/nu18203380 (PMC13708381; doi:10.3390/nu18203380)
Supplement: Supplementary file 1 [file nutrients-18-03380-s001.zip › Supplementary_Table_S1_Maaslin3results.pdf]

feature	metadata	value	name	coef	stderr	pval_individual	qval_individual	pval_joint	qval_joint	model	N	N_not_zero
Ruminococcus	Period	2	Period2	-8.99212775804032	0.774133863910048	3.42858872113009e-31	1.04571955994468e-28	6.8571774422602e-31	1.20000605239554e-28	prevalence	52	44
Ruminococcus	Treatment	Yeast	TreatmentYeast	7.14523702668518	0.756947044160498	3.74395302227278e-21	5.70952835896599e-19	7.4879060445456e-21	6.5519177889774e-19	prevalence	52	44
Subdoligranulum	Sequence	Yeast_Placebo	SequenceYeast_Placebo	10.9867529664634	1.175408218332	9.00151113392172e-21	9.15153631948708e-19	1.80030222678434e-20	1.05017629895753e-18	prevalence	52	47
Collinsella	Treatment	Yeast:TimePost	TreatmentYeast:TimePost	-9.28061484082578	1.0403782589651	4.64507812967824e-19	3.54187207387966e-17	9.29015625935647e-19	4.06444336346846e-17	prevalence	52	45
Subdoligranulum	Time	Post:Period2	TimePost:Period2	9.82001242733623	1.10636035203838	6.93315228634033e-19	4.2292228946676e-17	1.38663045726807e-18	4.85320660043824e-17	prevalence	52	47
Ruminococcus	Sequence	Yeast_Placebo	SequenceYeast_Placebo	8.12710693281997	0.931243481938098	2.61157251893994e-18	1.32754936379447e-16	5.22314503787988e-18	1.52341730271497e-16	prevalence	52	44
Ruminococcus	LogReadDepth	LogReadDepth	LogReadDepth	0.948049717860924	0.12057247542772	3.7533068889096e-15	1.63536943016776e-13	7.5066137778192e-15	1.8766534444548e-13	prevalence	52	44
Subdoligranulum	Treatment	Yeast:TimePost	TreatmentYeast:TimePost	-9.70948489835323	1.30821816362971	1.15438303449820e-13	4.4010853190244e-12	2.30876606899627e-13	5.05042577592934e-12	prevalence	52	47
Collinsella	Sequence	Yeast_Placebo	SequenceYeast_Placebo	9.82002910722446	1.46692190440307	2.16692498792971e-11	7.34346801465067e-10	4.33384997581245e-11	8.42693050852422e-10	prevalence	52	45
Collinsella	Time	Post:Period2	TimePost:Period2	6.58172287432016	1.07889798154807	1.05794997122704e-09	3.22674741224246e-08	2.11589994133481e-09	3.70282489733591e-08	prevalence	52	45
Unclassified__Enterobacteriaceae	LogReadDepth	LogReadDepth	LogReadDepth	-1.35577509371042	0.26731340880229	7.97972133993684e-06	0.000221255909880067	7.97972133993684e-06	0.000126950112226268	abundance	52	52
Ruminococcus	Time	Post:Period2	TimePost:Period2	3.37410705299443	0.883799589503851	0.000134685918744536	0.00342326710142363	0.000269353697192365	0.00392807475072199	prevalence	52	44
Erysipelotrichaceae_UCG-003	Period	2	Period2	8.01103929716171	2.25394111383305	0.000379078467903494	0.00889376405465889	0.00075801323532216	0.0102040243216445	prevalence	52	44
Erysipelotrichaceae_UCG-003	Time	Post:Period2	TimePost:Period2	-13.038650710606	3.94830061460001	0.000958800693856212	0.020888157973296	0.00191668208894188	0.0239585261117735	prevalence	52	44
Ruminococcus	Treatment	Yeast:TimePost	TreatmentYeast:TimePost	-3.28472710420038	1.05755081497893	0.00189652317520447	0.0385626378958242	0.00378944955025485	0.0414471044559124	prevalence	52	44
Erysipelotrichaceae_UCG-003	Treatment	Yeast:TimePost	TreatmentYeast:TimePost	12.6046690664139	4.13132457669227	0.00228080982365042	0.0434779372633362	0.00455641755384919	0.0469042983484475	prevalence	52	44
Bifidobacterium	Treatment	Yeast:TimePost	TreatmentYeast:TimePost	1.88099593669989	0.578708690935307	0.0027565488569115	0.0494557294916475	0.0027565488569115	0.0321597366639675	abundance	52	50
Erysipelotrichaceae_UCG-003	Sequence	Yeast_Placebo	SequenceYeast_Placebo	6.69963160996979	2.40228434624718	0.00528941361791285	0.0896261751924121	0.0105508493394043	0.102577701910875	prevalence	52	44
Parabacteroides	Treatment	Yeast	TreatmentYeast	2.83985859696205	1.00014813202852	0.0076789617820949	0.123267544396786	0.0152989571101389	0.133865874713715	abundance	52	41
Parabacteroides	Sequence	Yeast_Placebo	SequenceYeast_Placebo	-2.02489829459879	0.736014616896382	0.00956781789018388	0.133357541817038	0.019044092641188	0.145675595546286	abundance	52	41
Unclassified__Oscillospiraceae	LogReadDepth	LogReadDepth	LogReadDepth	-0.544151555922052	0.199434323034721	0.00961923252450764	0.133357541817038	0.0191459354146547	0.145675595546286	abundance	52	48
Christensenellaceae_R-7_group	LogReadDepth	LogReadDepth	LogReadDepth	1.20639571089931	0.464807679381116	0.00944608663207035	0.133357541817038	0.0188029447114801	0.145675595546286	prevalence	52	41
Subdoligranulum	LogReadDepth	LogReadDepth	LogReadDepth	0.321326858252205	0.126664796741691	0.0111861759149036	0.148338419741112	0.022247221298208	0.1622193219661	prevalence	52	47
Clostridium_sensu_stricto_1	Sequence	Yeast_Placebo	SequenceYeast_Placebo	2.04477964026859	0.70046603305809	0.014517447886601	0.177646166060846	0.014517447886601	0.133713335797641	abundance	52	47
Parabacteroides	Time	Post	TimePost	3.10418505574242	1.20803397071903	0.014885716228103	0.177646166060846	0.0295498479085824	0.194819633725096	abundance	52	41
Ruminococcus	Time	Post	TimePost	-2.02638487645889	0.834271843387444	0.0151436075986295	0.177646166060846	0.0300578863461577	0.194819633725096	prevalence	52	44
Anaerostipes	Period	2	Period2	-1.55545764246404	0.627916316128688	0.0191162824406341	0.215943190533089	0.0378671326269182	0.224377972265053	abundance	52	47
Clostridia_UCG-014	LogReadDepth	LogReadDepth	LogReadDepth	-0.647740721388729	0.263629318951267	0.024347206707284	0.265210644490058	0.0481016269401208	0.255084385288519	abundance	52	31
Parabacteroides	Treatment	Yeast:TimePost	TreatmentYeast:TimePost	-3.24433923197119	1.38537696715845	0.0253741787288322	0.265494423707851	0.0501045085115018	0.25789085263273	abundance	52	41
Unclassified__Lachnospiraceae	Period	2	Period2	-1.31028652058112	0.56914435667553	0.0261142056106083	0.265494423707851	0.0261142056106083	0.182799439274258	abundance	52	52
Unclassified__Enterobacteriaceae	Period	2	Period2	1.74888355422413	0.784473113984432	0.0323175246195099	0.31796274222421	0.0323175246195099	0.201984528871937	abundance	52	52
Bacteroides	Time	Post:Period2	TimePost:Period2	-2.617180025755	1.21224616322426	0.0384647952454377	0.334534453526474	0.0384647952454377	0.224377972265053	abundance	52	51
Christensenellaceae_R-7_group	LogReadDepth	LogReadDepth	LogReadDepth	-0.405451313131941	0.181585162706017	0.0354124377462681	0.334534453526474	0.0188029447114801	0.145675595546286	abundance	52	41
Clostridium_sensu_stricto_1	Treatment	Yeast:TimePost	TreatmentYeast:TimePost	-1.79346361809418	0.84168169194504	0.0416111283439729	0.334534453526474	0.0416111283439729	0.23490153097404	abundance	52	47
Faecalibacterium	Sequence	Yeast_Placebo	SequenceYeast_Placebo	-2.15215445802978	0.926965790716572	0.0392034765919835	0.334534453526474	0.0768700406070688	0.357097156589484	abundance	52	51
Faecalibacterium	Treatment	Yeast	TreatmentYeast	1.55497828790818	0.73077746202429	0.04090696556028	0.334534453526474	0.0801405512892101	0.357097156589484	abundance	52	51
Parabacteroides	Time	Post:Period2	TimePost:Period2	-2.98733899888728	1.40609522496683	0.0412018264313646	0.334534453526474	0.080706062361449	0.357097156589484	abundance	52	41
Erysipelotrichaceae_UCG-003	LogReadDepth	LogReadDepth	LogReadDepth	-0.890993077309698	0.437468099994989	0.0416797024065771	0.334534453526474	0.0816222072204534	0.357097156589484	prevalence	52	44

Clostridium_sensu_stricto_1	LogReadDepth	LogReadDepth	LogReadDepth	-0.476605925011206	0.230077041167323	0.0452285671541576	0.344867824550452	0.0452285671541576	0.2473437266243	abundance	52	47
Unclassified__Oscillospiraceae	Treatment	Yeast	TreatmentYeast	1.02466181531933	0.49039808787415	0.0451829778729801	0.344867824550452	0.08832445425649	0.376994621826482	abundance	52	48
Unclassified__Oscillospiraceae	LogReadDepth	LogReadDepth	LogReadDepth	2.62365492501211	1.34008684834851	0.0502506041708706	0.373815470051599	0.0191459354146547	0.145675595546286	prevalence	52	48
Bifidobacterium	Sequence	Yeast_Placebo	SequenceYeast_Placebo	-0.878872483833319	0.411763611050224	0.0554504699450719	0.375830962961043	0.0554504699450719	0.27725234972536	abundance	52	50
Dialister	Treatment	Yeast	TreatmentYeast	1.75382919045119	0.867179059820649	0.054317896356427	0.375830962961043	0.105685358848266	0.430114832522015	abundance	52	41
Subdoligranulum	LogReadDepth	LogReadDepth	LogReadDepth	-0.57525522412433	0.288531061953584	0.0548828642767193	0.375830962961043	0.022247221298208	0.1622193219661	abundance	52	47
Clostridia_UCG-014	LogReadDepth	LogReadDepth	LogReadDepth	-1.98443718847723	1.03573736540806	0.0553694713089392	0.375830962961043	0.0481016269401208	0.255084385288519	prevalence	52	31
Collinsella	Treatment	Yeast	TreatmentYeast	-1.68312066253692	0.85253482567498	0.0583159791940545	0.386660296830144	0.113231204958748	0.440343574839574	abundance	52	45
Alistipes	Treatment	Yeast	TreatmentYeast	1.81348503037928	0.931928472535627	0.0604386924851465	0.39220853633978	0.117224549420979	0.442630270891951	abundance	52	48
Erysipelotrichaceae_UCG-003	Treatment	Yeast	TreatmentYeast	-4.62436815222042	2.48415517957287	0.0626671669124558	0.398197623089563	0.121407160016078	0.442630270891951	prevalence	52	44
Bacteroides	Treatment	Yeast	TreatmentYeast	1.6263392691624	0.867161061903542	0.0697499965737643	0.432482674915629	0.0697499965737643	0.339062483344688	abundance	52	51
Streptococcus	LogReadDepth	LogReadDepth	LogReadDepth	1.74014092405724	0.963466435874185	0.0708987991664966	0.432482674915629	0.136770958609742	0.488467709320507	prevalence	52	48
Dialister	Period	2	Period2	-1.62705531818757	0.885291193546241	0.0779452366298131	0.466143081805745	0.149815013346349	0.52435254671222	abundance	52	41
[Eubacterium]_coprostanoligenes_group	Period	2	Period2	-7.35387333668775	4.25847278014151	0.0841891384592219	0.493801677501205	0.161290465883938	0.542804452494021	prevalence	52	43
[Eubacterium]_coprostanoligenes_group	Time	Post	TimePost	-1.75233078803312	0.985430853059496	0.0869568343934763	0.500411971509628	0.166352177739218	0.543216626198354	abundance	52	43
Unclassified__Peptostreptococcaceae	Sequence	Yeast_Placebo	SequenceYeast_Placebo	1.67360848739578	0.879219623175542	0.0896360575376681	0.506277732388681	0.17123749226444	0.543216626198354	abundance	52	49
Dialister	Sequence	Yeast_Placebo	SequenceYeast_Placebo	-1.70368579456053	0.89815174795463	0.0957226949563016	0.511624747350075	0.182282555582906	0.543216626198354	abundance	52	41
Streptococcus	LogReadDepth	LogReadDepth	LogReadDepth	0.43198401606548	0.252725864364606	0.0951443966939513	0.511624747350075	0.136770958609742	0.488467709320507	abundance	52	48
Unclassified__Lachnospiraceae	LogReadDepth	LogReadDepth	LogReadDepth	0.296501920857358	0.175911969256066	0.098970033093949	0.511624747350075	0.098970033093949	0.412375137891454	abundance	52	52
[Eubacterium]_coprostanoligenes_group	Sequence	Yeast_Placebo	SequenceYeast_Placebo	7.01204881301749	4.18229482242033	0.0936201038692112	0.511624747350075	0.178475483889941	0.543216626198354	prevalence	52	43
Collinsella	Period	2	Period2	1.60617597313578	0.970476503314333	0.097916689219263	0.511624747350075	0.186245700410864	0.543216626198354	prevalence	52	45
Akkermansia	Period	2	Period2	-2.3896555841928	1.44848568411855	0.10793946463036	0.531503985377962	0.204228001236032	0.566661363246377	abundance	52	43
Blautia	Treatment	Yeast:TimePost	TreatmentYeast:TimePost	1.38608300591997	0.839897807537336	0.108249131588748	0.531503985377962	0.108249131588748	0.430536318818883	abundance	52	52
Erysipelotrichaceae_UCG-003	LogReadDepth	LogReadDepth	LogReadDepth	0.332152173087169	0.202635229927698	0.112405155363628	0.531503985377962	0.0816222072204534	0.357097156589484	abundance	52	44
[Eubacterium]_coprostanoligenes_group	Treatment	Yeast	TreatmentYeast	6.58194659166619	4.11917923216741	0.11007009228587	0.531503985377962	0.20802475935592	0.566661363246377	prevalence	52	43
Christensenellaceae_R-7_group	Period	2	Period2	-9.46684376418476	5.84407497776353	0.105252778006226	0.531503985377962	0.199427408734425	0.566661363246377	prevalence	52	41
Collinsella	Time	Post	TimePost	1.33145091268016	0.840743441570227	0.113271341146123	0.531503985377962	0.213712285567205	0.566661363246377	prevalence	52	45
[Eubacterium]_coprostanoligenes_group	Period	2	Period2	-1.20859589654034	0.769775555881637	0.127915177750782	0.557943759275634	0.161290465883938	0.542804452494021	abundance	52	43
Parabacteroides	LogReadDepth	LogReadDepth	LogReadDepth	-0.511638930718564	0.327577342035222	0.127854659691089	0.557943759275634	0.239362505377454	0.607954684016045	abundance	52	41
Unclassified__Enterobacteriaceae	Treatment	Yeast	TreatmentYeast	-1.14422636086989	0.719184978541948	0.121148323879069	0.557943759275634	0.121148323879069	0.442630270891951	abundance	52	52
[Eubacterium]_coprostanoligenes_group	Time	Post:Period2	TimePost:Period2	12.3724793572785	8.13002192448375	0.12805266606326	0.557943759275634	0.239707846840612	0.607954684016045	prevalence	52	43
Christensenellaceae_R-7_group	Sequence	Yeast_Placebo	SequenceYeast_Placebo	8.60011800734116	5.63702650566554	0.127097560357563	0.557943759275634	0.238041330866282	0.607954684016045	prevalence	52	41
Unclassified__Ruminococcaceae	LogReadDepth	LogReadDepth	LogReadDepth	0.864012568423881	0.576288697440981	0.133803505927165	0.574789708560357	0.249703633655929	0.624259084139822	prevalence	52	48
Collinsella	Treatment	Yeast:TimePost	TreatmentYeast:TimePost	1.83207979854532	1.19669434132297	0.137673349334212	0.583199604818538	9.29015625935647e-19	4.06444336346846e-17	abundance	52	45
Christensenellaceae_R-7_group	Period	2	Period2	0.805993300672226	0.532124437244771	0.143569424446038	0.592633496287028	0.199427408734425	0.566661363246377	abundance	52	41
Ruminococcus	Treatment	Yeast:TimePost	TreatmentYeast:TimePost	-1.29141272119611	0.857003712932974	0.143830016419707	0.592633496287028	0.00378944955025485	0.0414471044559124	abundance	52	44
Unclassified__Peptostreptococcaceae	LogReadDepth	LogReadDepth	LogReadDepth	1.07349821939118	0.737909886521553	0.145729548267302	0.592633496287028	0.270221995296412	0.6655200361714	prevalence	52	49
Dialister	LogReadDepth	LogReadDepth	LogReadDepth	0.982800221038619	0.679093873739811	0.147834498315668	0.593283184029983	0.27381395773909	0.6655200361714	prevalence	52	41
Bacteroides	Period	2	Period2	1.37932311691247	0.954639608388064	0.157495383545068	0.617643786759304	0.157495383545068	0.540425335693862	abundance	52	51

Ruminococcus	Treatment	Yeast	TreatmentYeast	0.89261059321128	0.616427791610881	0.159081941399334	0.617643786759304	7.4879060445456e-21	6.5519177889774e-19	abundance	52	44
[Eubacterium]_coprostanoligenes_group	Time	Post	TimePost	-2.52403519882483	1.79628826626698	0.159979866078639	0.617643786759304	0.166352177739218	0.543216626198354	prevalence	52	43
[Eubacterium]_coprostanoligenes_group	Treatment	Yeast:TimePost	TreatmentYeast:TimePost	-11.1878148945522	8.02177008555051	0.163111885050635	0.621864061755545	0.299618283056498	0.718263007327221	prevalence	52	43
Bifidobacterium	Period	2	Period2	-0.630225583987703	0.457676501189635	0.177401251369999	0.661340272064249	0.177401251369999	0.543216626198354	abundance	52	50
Bifidobacterium	Time	Post	TimePost	-0.690916276696824	0.508072665903109	0.183575928397079	0.661340272064249	0.183575928397079	0.543216626198354	abundance	52	50
Christensenellaceae_R-7_group	Time	Post:Period2	TimePost:Period2	-0.958915449623089	0.701257823922811	0.184680995171386	0.661340272064249	0.335254920365278	0.776596405575682	abundance	52	41
Clostridium_sensu_stricto_1	Treatment	Yeast	TreatmentYeast	0.840249425829631	0.618100635630561	0.183990148853626	0.661340272064249	0.183990148853626	0.543216626198354	abundance	52	47
Faecalibacterium	Treatment	Yeast:TimePost	TreatmentYeast:TimePost	-1.36117185955684	1.01179641948161	0.187827466682726	0.661340272064249	0.340375776125001	0.776596405575682	abundance	52	51
Unclassified__Peptostreptococcaceae	Time	Post:Period2	TimePost:Period2	1.47187237053906	1.08389535306026	0.185282927494964	0.661340272064249	0.336236091768824	0.776596405575682	abundance	52	49
Alistipes	LogReadDepth	LogReadDepth	LogReadDepth	0.670265726853952	0.509863136110896	0.188644602195376	0.661340272064249	0.3417024184533	0.776596405575682	prevalence	52	48
Anaerostipes	Time	Post	TimePost	-0.925954944473626	0.697944588857072	0.195210665611867	0.676582420586583	0.352314127255105	0.780442686957511	abundance	52	47
Blautia	Sequence	Yeast_Placebo	SequenceYeast_Placebo	-0.651095776518865	0.481973280669929	0.201827617127797	0.691656440718855	0.201827617127797	0.566661363246377	abundance	52	52
Anaerostipes	Time	Post:Period2	TimePost:Period2	1.01666496452918	0.787296924624099	0.207059091352719	0.693989262226147	0.371244715393625	0.802071915973881	abundance	52	47
Christensenellaceae_R-7_group	Treatment	Yeast	TreatmentYeast	7.01667362337764	5.54233394981145	0.205507989272784	0.693989262226147	0.368782444890626	0.802071915973881	prevalence	52	41
Unclassified__Lachnospiraceae	Time	Post	TimePost	-0.82513504616926	0.65039421498393	0.211230090918427	0.700273670979569	0.211230090918427	0.566661363246377	abundance	52	52
Ruminococcus	Period	2	Period2	-0.854957627143157	0.673751911642787	0.214946214741455	0.704931134367138	6.8571774422602e-31	1.20000605239554e-28	abundance	52	44
Alistipes	Time	Post:Period2	TimePost:Period2	-1.6160397998424	1.28398806238673	0.217859069896559	0.706883152323942	0.388255565456924	0.818611131987491	abundance	52	48
Akkermansia	Treatment	Yeast	TreatmentYeast	1.68728991079408	1.37931080102381	0.229394662807775	0.727678396721696	0.406167414290857	0.836327970478074	abundance	52	43
Faecalibacterium	Time	Post:Period2	TimePost:Period2	-1.25177344500991	1.02246536066182	0.229587007066576	0.727678396721696	0.406463820319365	0.836327970478074	abundance	52	51
Christensenellaceae_R-7_group	Time	Post:Period2	TimePost:Period2	3.00904461011164	2.51446313422893	0.231425588465588	0.727678396721696	0.335254920365278	0.776596405575682	prevalence	52	41
Dialister	Treatment	Yeast:TimePost	TreatmentYeast:TimePost	-1.42444265717223	1.17196005660241	0.23718283667123	0.738171073313521	0.418109975331048	0.836327970478074	abundance	52	41
Subdoligranulum	Time	Post:Period2	TimePost:Period2	-1.24231061669619	1.05130472819194	0.247813767001769	0.76346665591454	1.38663045726807e-18	4.85320660043824e-17	abundance	52	47
Unclassified__Ruminococcaceae	LogReadDepth	LogReadDepth	LogReadDepth	-0.368201370121511	0.315403478788717	0.251104944043645	0.765870079333118	0.249703633655929	0.624259084139822	abundance	52	48
Alistipes	Treatment	Yeast:TimePost	TreatmentYeast:TimePost	-1.42517353931123	1.26806247658295	0.269992549345167	0.806765057114837	0.467089121988432	0.880104823130878	abundance	52	48
Anaerostipes	Treatment	Yeast:TimePost	TreatmentYeast:TimePost	0.872599552401003	0.783397939394902	0.274793289045446	0.806765057114837	0.474075226386477	0.880104823130878	abundance	52	47
Faecalibacterium	Time	Post	TimePost	0.989871477865221	0.891665760134336	0.275093658819485	0.806765057114837	0.47451079651628	0.880104823130878	abundance	52	51
Christensenellaceae_R-7_group	Treatment	Yeast:TimePost	TreatmentYeast:TimePost	-2.60433472344631	2.35931635467201	0.269657592417281	0.806765057114837	0.466599967686278	0.880104823130878	prevalence	52	41
Escherichia-Shigella	Period	2	Period2	1.29535527141115	1.1788226658373	0.279485942966665	0.811840120046028	0.480859493617365	0.880104823130878	abundance	52	50
Akkermansia	Treatment	Yeast:TimePost	TreatmentYeast:TimePost	-2.56415688624396	2.38802179073122	0.282931274159364	0.814094703949114	0.485812442421287	0.880104823130878	prevalence	52	43
Parabacteroides	Sequence	Yeast_Placebo	SequenceYeast_Placebo	-1.06255059251387	1.0098572831055	0.292717447702947	0.834381509807467	0.019044092641188	0.145675595546286	prevalence	52	41
Parabacteroides	Period	2	Period2	1.12414326354876	1.06220658588887	0.297599982324186	0.83722852144275	0.506634215169016	0.880104823130878	abundance	52	41
Unclassified__Ruminococcaceae	Treatment	Yeast	TreatmentYeast	0.840320405576458	0.795319829595102	0.299206258482819	0.83722852144275	0.50888813185035	0.880104823130878	abundance	52	48
Alistipes	Sequence	Yeast_Placebo	SequenceYeast_Placebo	-1.03320252030518	0.956829219596992	0.302128509412266	0.837719957915829	0.512975382624854	0.880104823130878	abundance	52	48
Ruminococcus	Time	Post:Period2	TimePost:Period2	-0.90680881030797	0.870197926043492	0.306845892746388	0.843135110699535	0.000269353697192365	0.00392807475072199	abundance	52	44
Akkermansia	Sequence	Yeast_Placebo	SequenceYeast_Placebo	-1.02021591570401	1.01993943672485	0.324045027944159	0.870224678566449	0.543084875752987	0.896602389214837	abundance	52	43
Streptococcus	Period	2	Period2	-0.76558712086	0.765209997807008	0.323086316484096	0.870224678566449	0.541787865068931	0.896602389214837	abundance	52	48
Dialister	Treatment	Yeast	TreatmentYeast	-1.52157691422329	1.54679363279578	0.325264306087132	0.870224678566449	0.105685358848266	0.430114832522015	prevalence	52	41
Collinsella	Sequence	Yeast_Placebo	SequenceYeast_Placebo	-0.783997960116237	0.772257834117962	0.334627999666326	0.887491651288952	4.33384997581245e-11	8.42693050852422e-10	abundance	52	45
Blautia	Time	Post	TimePost	-0.70693837495054	0.743413443328931	0.348482276577439	0.895291396311277	0.348482276577439	0.780442686957511	abundance	52	52

Clostridia_UCG-014	Time	Post	TimePost	-0.696646774600568	0.721288211604254	0.35080177080089	0.895291396311277	0.57854165920474	0.928851287714032	abundance	52	31
Bifidobacterium	LogReadDepth	LogReadDepth	LogReadDepth	0.644422878379913	0.684041273900644	0.346150728940284	0.895291396311277	0.572481130734678	0.927631461838598	prevalence	52	50
Dialister	Time	Post:Period2	TimePost:Period2	-1.97070730182159	2.09011089829106	0.345746314967198	0.895291396311277	0.571952115620999	0.927631461838598	prevalence	52	41
Parabacteroides	Treatment	Yeast:TimePost	TreatmentYeast:TimePost	1.56520738068085	1.68258083809049	0.352245795270011	0.895291396311277	0.0501045085115018	0.25789085263273	prevalence	52	41
Streptococcus	Sequence	Yeast_Placebo	SequenceYeast_Placebo	0.487260112209684	0.530892018061374	0.364217504046475	0.910437657312013	0.595780617839106	0.939293766863455	abundance	52	48
Streptococcus	Treatment	Yeast:TimePost	TreatmentYeast:TimePost	-0.936264154423854	1.01932104265611	0.36385377172128	0.910437657312013	0.595317976246758	0.939293766863455	abundance	52	48
Dialister	Treatment	Yeast:TimePost	TreatmentYeast:TimePost	1.80741519022387	2.00421802562734	0.367160104424189	0.910437657312013	0.418109975331048	0.836327970478074	prevalence	52	41
Subdoligranulum	Period	2	Period2	0.736587504772344	0.813882090798429	0.37310818436501	0.917725776059097	0.607006651489866	0.944161179361028	abundance	52	47
Blautia	Treatment	Yeast	TreatmentYeast	-0.536245036532585	0.607298846868459	0.383377392310074	0.928016703607718	0.383377392310074	0.818183459198328	abundance	52	52
Subdoligranulum	Treatment	Yeast	TreatmentYeast	-1.11820587059162	1.27561479636843	0.3807031128173	0.928016703607718	0.616471365525818	0.944161179361028	prevalence	52	47
Erysipelotrichaceae_UCG-003	Period	2	Period2	-0.507257414824818	0.583661577539808	0.392587518454737	0.942828292351928	0.00075801323532216	0.0102040243216445	abundance	52	44
Akkermansia	Time	Post:Period2	TimePost:Period2	1.61338810127491	1.93804009788496	0.410780189949379	0.963753522573544	0.65282001544391	0.944161179361028	abundance	52	43
Anaerostipes	LogReadDepth	LogReadDepth	LogReadDepth	0.186313407706412	0.221400940829176	0.40579622250805	0.963753522573544	0.646921870814297	0.944161179361028	abundance	52	47
Unclassified__Oscillospiraceae	Treatment	Yeast:TimePost	TreatmentYeast:TimePost	-0.586746461333072	0.700374614887739	0.408755152560229	0.963753522573544	0.650429530375922	0.944161179361028	abundance	52	48
Unclassified__Enterobacteriaceae	Time	Post	TimePost	0.722674782893839	0.876414275811479	0.415666344259143	0.967772786252204	0.415666344259143	0.836327970478074	abundance	52	52
Escherichia-Shigella	Treatment	Yeast:TimePost	TreatmentYeast:TimePost	-1.22949566683505	1.50634287638055	0.420553493726117	0.971733451412619	0.420553493726117	0.836327970478074	abundance	52	50
Erysipelotrichaceae_UCG-003	Sequence	Yeast_Placebo	SequenceYeast_Placebo	0.988083181046126	1.19065249704949	0.426332828608187	0.977680546808248	0.0105508493394043	0.102577701910875	abundance	52	44
[Eubacterium]_coprostanoligenes_group	LogReadDepth	LogReadDepth	LogReadDepth	-0.164937272015447	0.264095155316827	0.53643636628814	0.984922076553615	0.690445442187999	0.966113797810981	abundance	52	43
[Eubacterium]_coprostanoligenes_group	Sequence	Yeast_Placebo	SequenceYeast_Placebo	0.552445945002072	0.829923117610554	0.519019524189421	0.984922076553615	0.178475483889941	0.543216626198354	abundance	52	43
[Eubacterium]_hallii_group	LogReadDepth	LogReadDepth	LogReadDepth	0.177433078537395	0.25264524068711	0.486477477762236	0.984922076553615	0.736294619154565	0.966113797810981	abundance	52	51
[Eubacterium]_hallii_group	Period	2	Period2	-0.463137746787358	0.74660333796703	0.539285202571979	0.984922076553615	0.787741875430858	0.966113797810981	abundance	52	51
[Eubacterium]_hallii_group	Sequence	Yeast_Placebo	SequenceYeast_Placebo	0.568471356408413	0.81023187048009	0.498777542213678	0.984922076553615	0.748776047810638	0.966113797810981	abundance	52	51
Akkermansia	LogReadDepth	LogReadDepth	LogReadDepth	-0.283627284432311	0.447517873538552	0.530345662894343	0.984922076553615	0.779424803637846	0.966113797810981	abundance	52	43
Akkermansia	Treatment	Yeast:TimePost	TreatmentYeast:TimePost	-1.40049916952917	1.9357271534177	0.474180549341996	0.984922076553615	0.485812442421287	0.880104823130878	abundance	52	43
Alistipes	LogReadDepth	LogReadDepth	LogReadDepth	-0.276097222041245	0.350311999744639	0.435264778074156	0.984922076553615	0.3417024184533	0.776596405575682	abundance	52	48
Bacteroides	Sequence	Yeast_Placebo	SequenceYeast_Placebo	-0.677794251876018	0.993110696648557	0.509242839153259	0.984922076553615	0.509242839153259	0.880104823130878	abundance	52	51
Bacteroides	Time	Post	TimePost	0.70892777539248	1.05850919500865	0.507868781913501	0.984922076553615	0.507868781913501	0.880104823130878	abundance	52	51
Bifidobacterium	Time	Post:Period2	TimePost:Period2	0.430547361942042	0.58360534209342	0.466126722183705	0.984922076553615	0.466126722183705	0.880104823130878	abundance	52	50
Bifidobacterium	Treatment	Yeast	TreatmentYeast	0.278202319481328	0.424118272107577	0.516422032102974	0.984922076553615	0.766152348964583	0.966113797810981	abundance	52	50
Blautia	Period	2	Period2	-0.443003471045559	0.65438429033355	0.502627543645612	0.984922076553615	0.502627543645612	0.880104823130878	abundance	52	52
Christensenellaceae_R-7_group	Time	Post	TimePost	0.496911818156338	0.648308559654503	0.451026409486919	0.984922076553615	0.698627996919176	0.966113797810981	abundance	52	41
Clostridia_UCG-014	Period	2	Period2	0.477991497896407	0.707802258289454	0.509401192833934	0.984922076553615	0.759312810407233	0.966113797810981	abundance	52	31
Clostridia_UCG-014	Sequence	Yeast_Placebo	SequenceYeast_Placebo	-0.940294523127405	1.28044820001555	0.487224619709302	0.984922076553615	0.737061409367731	0.966113797810981	abundance	52	31
Clostridium_sensu_stricto_1	Time	Post:Period2	TimePost:Period2	0.527119914613709	0.848423087267624	0.539133066293233	0.984922076553615	0.539133066293233	0.896602389214837	abundance	52	47
Collinsella	Period	2	Period2	-0.621675226681405	0.914608963237076	0.50182382202302	0.984922076553615	0.186245700410864	0.543216626198354	abundance	52	45
Ruminococcus	LogReadDepth	LogReadDepth	LogReadDepth	-0.181464784323575	0.242491206821056	0.459581167851525	0.984922076553615	7.5066137778192e-15	1.8766534444548e-13	abundance	52	44
Streptococcus	Time	Post	TimePost	0.604075346032687	0.947651535845056	0.527467390340983	0.984922076553615	0.77671293280884	0.966113797810981	abundance	52	48
Streptococcus	Treatment	Yeast	TreatmentYeast	0.536730447542989	0.705547678661771	0.45128038686801	0.984922076553615	0.69890678616428	0.966113797810981	abundance	52	48
Subdoligranulum	Time	Post	TimePost	0.696526428766952	0.935759203021296	0.463248766589671	0.984922076553615	0.71189811343249	0.966113797810981	abundance	52	47

Unclassified__Lachnospiraceae	Sequence	Yeast_Placebo	SequenceYeast_Placebo	0.246173060837452	0.383684266324426	0.524459328961706	0.984922076553615	0.524459328961706	0.891071675420375	abundance	52	52
Unclassified__Lachnospiraceae	Treatment	Yeast:TimePost	TreatmentYeast:TimePost	0.530307983298802	0.734698613445757	0.474233918183439	0.984922076553615	0.474233918183439	0.880104823130878	abundance	52	52
Unclassified__Oscillospiraceae	Sequence	Yeast_Placebo	SequenceYeast_Placebo	-0.480639782866316	0.610485763710503	0.446188984537409	0.984922076553615	0.693293359152294	0.966113797810981	abundance	52	48
Unclassified__Peptostreptococcaceae	Treatment	Yeast:TimePost	TreatmentYeast:TimePost	-0.721735418826367	1.0683444173233	0.504988431973672	0.984922076553615	0.754963547520116	0.966113797810981	abundance	52	49
Unclassified__Ruminococcaceae	Sequence	Yeast_Placebo	SequenceYeast_Placebo	0.844721075315974	1.32672035238794	0.537471701231286	0.984922076553615	0.786067572838119	0.966113797810981	abundance	52	48
[Eubacterium]_coprostanoligenes_group	LogReadDepth	LogReadDepth	LogReadDepth	0.397352599351387	0.518677241816537	0.443623726411702	0.984922076553615	0.690445442187999	0.966113797810981	prevalence	52	43
Akkermansia	Period	2	Period2	-6.18557261131659	9.15705972386032	0.499359624003621	0.984922076553615	0.204228001236032	0.566661363246377	prevalence	52	43
Akkermansia	Sequence	Yeast_Placebo	SequenceYeast_Placebo	-5.68025454650868	8.97874597500378	0.526973074132856	0.984922076553615	0.543084875752987	0.896602389214837	prevalence	52	43
Anaerostipes	Sequence	Yeast_Placebo	SequenceYeast_Placebo	-0.800545508349621	1.12641615679256	0.477269309135828	0.984922076553615	0.726752624828666	0.966113797810981	prevalence	52	47
Collinsella	LogReadDepth	LogReadDepth	LogReadDepth	0.0918393734944802	0.148803414895049	0.537112097346833	0.984922076553615	0.785734789577352	0.966113797810981	prevalence	52	45
Parabacteroides	LogReadDepth	LogReadDepth	LogReadDepth	0.28287990361066	0.380219364608988	0.456881731688343	0.984922076553615	0.239362505377454	0.607954684016045	prevalence	52	41
Parabacteroides	Time	Post	TimePost	-1.02270073164453	1.4963481073783	0.494313408509853	0.984922076553615	0.0295498479085824	0.194819633725096	prevalence	52	41
Unclassified__Ruminococcaceae	Treatment	Yeast:TimePost	TreatmentYeast:TimePost	-0.664325434248841	1.09136551255752	0.547436993766247	0.993858827968483	0.795186725388668	0.966113797810981	abundance	52	48
[Eubacterium]_coprostanoligenes_group	Time	Post:Period2	TimePost:Period2	0.190139569753785	1.05560888526416	0.858467775898505	0.999999980705644	0.239707846840612	0.607954684016045	abundance	52	43
[Eubacterium]_coprostanoligenes_group	Treatment	Yeast	TreatmentYeast	-0.220179156678798	0.715767491413646	0.760760157446734	0.999999980705644	0.20802475935592	0.566661363246377	abundance	52	43
[Eubacterium]_coprostanoligenes_group	Treatment	Yeast:TimePost	TreatmentYeast:TimePost	0.323447183250666	1.03327482278749	0.75676422471388	0.999999980705644	0.299618283056498	0.718263007327221	abundance	52	43
[Eubacterium]_hallii_group	Time	Post	TimePost	0.123820781279817	0.84865383891716	0.884940826852424	0.999999980705644	0.986761386674596	0.992432429126749	abundance	52	51
[Eubacterium]_hallii_group	Time	Post:Period2	TimePost:Period2	-0.153540457510486	0.947037543214678	0.872255028453612	0.999999980705644	0.983681222244613	0.992432429126749	abundance	52	51
[Eubacterium]_hallii_group	Treatment	Yeast	TreatmentYeast	-0.246102765121065	0.687163073044757	0.722623373298491	0.999999980705644	0.923062206959692	0.992432429126749	abundance	52	51
[Eubacterium]_hallii_group	Treatment	Yeast:TimePost	TreatmentYeast:TimePost	0.418756437819984	0.935958419054505	0.657710438682946	0.999999980705644	0.882837856213378	0.984054935269689	abundance	52	51
Akkermansia	Time	Post	TimePost	-0.256938388235447	1.60622467274861	0.873828937792291	0.999999980705644	0.856878811879499	0.979300481328594	abundance	52	43
Alistipes	Period	2	Period2	0.573665873625184	0.999184393907487	0.569896282969534	0.999999980705644	0.815010792596576	0.966113797810981	abundance	52	48
Alistipes	Time	Post	TimePost	0.566173798960823	1.13981756792423	0.622968623691102	0.999999980705644	0.857847341278618	0.979300481328594	abundance	52	48
Anaerostipes	Sequence	Yeast_Placebo	SequenceYeast_Placebo	0.423857778855682	0.783583794776979	0.599288071445308	0.999999980705644	0.726752624828666	0.966113797810981	abundance	52	47
Anaerostipes	Treatment	Yeast	TreatmentYeast	-0.260223098900577	0.589931473484076	0.662260129481536	0.999999980705644	0.861784423569162	0.979300481328594	abundance	52	47
Bacteroides	LogReadDepth	LogReadDepth	LogReadDepth	-0.159259478926649	0.340588728419244	0.642470669758714	0.999999980705644	0.642470669758714	0.944161179361028	abundance	52	51
Bacteroides	Treatment	Yeast:TimePost	TreatmentYeast:TimePost	-0.551482857805969	1.20049387459529	0.649089298055435	0.999999980705644	0.649089298055435	0.944161179361028	abundance	52	51
Bifidobacterium	LogReadDepth	LogReadDepth	LogReadDepth	0.0334742845398587	0.156422779961077	0.831585109893388	0.999999980705644	0.572481130734678	0.927631461838598	abundance	52	50
Blautia	LogReadDepth	LogReadDepth	LogReadDepth	0.069688650526205	0.207944551248758	0.73916337633509	0.999999980705644	0.73916337633509	0.966113797810981	abundance	52	52
Blautia	Time	Post:Period2	TimePost:Period2	-0.0381394932161519	0.849630121180744	0.964458763957116	0.999999980705644	0.964458763957116	0.992432429126749	abundance	52	52
Christensenellaceae_R-7_group	Sequence	Yeast_Placebo	SequenceYeast_Placebo	0.347422439919563	1.08991861576299	0.756016183020541	0.999999980705644	0.238041330866282	0.607954684016045	abundance	52	41
Christensenellaceae_R-7_group	Treatment	Yeast	TreatmentYeast	-0.0179605754715468	0.495931393739883	0.971422485172676	0.999999980705644	0.368782444890626	0.802071915973881	abundance	52	41
Christensenellaceae_R-7_group	Treatment	Yeast:TimePost	TreatmentYeast:TimePost	-0.282320852301515	0.690927275622056	0.686569733231394	0.999999980705644	0.466599967686278	0.880104823130878	abundance	52	41
Clostridia_UCG-014	Time	Post:Period2	TimePost:Period2	-0.2258704175116	0.860452073231445	0.796756592514334	0.999999980705644	0.958692117313615	0.992432429126749	abundance	52	31
Clostridia_UCG-014	Treatment	Yeast	TreatmentYeast	-0.208924511890841	0.635151858450156	0.746842641997317	0.999999980705644	0.81705624043443	0.966113797810981	abundance	52	31
Clostridia_UCG-014	Treatment	Yeast:TimePost	TreatmentYeast:TimePost	0.472051328973935	0.861651902804298	0.59236828601076	0.999999980705644	0.833836385750195	0.975336538215412	abundance	52	31
Clostridium_sensu_stricto_1	Period	2	Period2	-0.174296815158786	0.668957014133992	0.796102568843332	0.999999980705644	0.796102568843332	0.966113797810981	abundance	52	47
Clostridium_sensu_stricto_1	Time	Post	TimePost	0.19715569290917	0.748171871989266	0.793964930981761	0.999999980705644	0.793964930981761	0.966113797810981	abundance	52	47
Collinsella	LogReadDepth	LogReadDepth	LogReadDepth	-0.0518694108590862	0.290387626245622	0.859209575782941	0.999999980705644	0.785734789577352	0.966113797810981	abundance	52	45

Collinsella	Time	Post	TimePost	0.0373324018159346	1.09657596280612	0.973095593439092	0.999999980705644	0.213712285567205	0.566661363246377	abundance	52	45
Collinsella	Time	Post:Period2	TimePost:Period2	-0.340399230402247	1.20135688998468	0.779057896927583	0.999999980705644	2.11589994133481e-09	3.70282489733591e-08	abundance	52	45
Dialister	LogReadDepth	LogReadDepth	LogReadDepth	0.0268505754349684	0.338833476744546	0.937318168843652	0.999999980705644	0.27381395773909	0.6655200361714	abundance	52	41
Dialister	Time	Post	TimePost	-0.204270659468247	1.02173245739943	0.843382305096022	0.999999980705644	0.975470897642964	0.992432429126749	abundance	52	41
Dialister	Time	Post:Period2	TimePost:Period2	-0.509261417137735	1.16961144283242	0.66758206171546	0.999999980705644	0.571952115620999	0.927631461838598	abundance	52	41
Erysipelotrichaceae_UCG-003	Time	Post	TimePost	0.0312027364014322	0.657778346607884	0.962534081366222	0.999999980705644	0.97112845197747	0.992432429126749	abundance	52	44
Erysipelotrichaceae_UCG-003	Time	Post:Period2	TimePost:Period2	-0.341170300233858	0.714564023410974	0.637084648447518	0.999999980705644	0.00191668208894188	0.0239585261117735	abundance	52	44
Erysipelotrichaceae_UCG-003	Treatment	Yeast	TreatmentYeast	-0.131110397776688	0.511831679983609	0.799846634618547	0.999999980705644	0.121407160016078	0.442630270891951	abundance	52	44
Erysipelotrichaceae_UCG-003	Treatment	Yeast:TimePost	TreatmentYeast:TimePost	0.0943405830569411	0.710895547059945	0.895462320668843	0.999999980705644	0.00455641755384919	0.0469042983484475	abundance	52	44
Escherichia-Shigella	LogReadDepth	LogReadDepth	LogReadDepth	0.204726106497402	0.408986110063653	0.61928908020987	0.999999980705644	0.61928908020987	0.944161179361028	abundance	52	50
Escherichia-Shigella	Sequence	Yeast_Placebo	SequenceYeast_Placebo	0.144306550035393	1.02580309525696	0.890678539405205	0.999999980705644	0.890678539405205	0.986511040480449	abundance	52	50
Escherichia-Shigella	Time	Post	TimePost	0.756056419197343	1.31954034125537	0.570782383156127	0.999999980705644	0.815772237390866	0.966113797810981	abundance	52	50
Escherichia-Shigella	Time	Post:Period2	TimePost:Period2	-0.691822379348138	1.51630741291906	0.651334804862846	0.999999980705644	0.651334804862846	0.944161179361028	abundance	52	50
Escherichia-Shigella	Treatment	Yeast	TreatmentYeast	-0.326347225206065	1.10758924770374	0.770147735008556	0.999999980705644	0.770147735008556	0.966113797810981	abundance	52	50
Faecalibacterium	LogReadDepth	LogReadDepth	LogReadDepth	-0.0461200391901011	0.288408486365901	0.873739472999409	0.999999980705644	0.836002747041782	0.975336538215412	abundance	52	51
Faecalibacterium	Period	2	Period2	-0.14681850178342	0.805178530323249	0.856379122827816	0.999999980705644	0.979373043640293	0.992432429126749	abundance	52	51
Ruminococcus	Sequence	Yeast_Placebo	SequenceYeast_Placebo	-0.0723511978023038	0.893283668665486	0.936989925724177	0.999999980705644	5.22314503787988e-18	1.52341730271497e-16	abundance	52	44
Ruminococcus	Time	Post	TimePost	0.0988007301854902	0.789037910094156	0.901300564375045	0.999999980705644	0.0300578863461577	0.194819633725096	abundance	52	44
Streptococcus	Time	Post:Period2	TimePost:Period2	0.00617688596512325	1.0401880679295	0.995291515952848	0.999999980705644	0.999977830177978	0.999977830177978	abundance	52	48
Subdoligranulum	Sequence	Yeast_Placebo	SequenceYeast_Placebo	0.624233902671932	1.26508369185677	0.63272837710538	0.999999980705644	1.80030222678434e-20	1.05017629895753e-18	abundance	52	47
Subdoligranulum	Treatment	Yeast	TreatmentYeast	0.191190943451205	0.75429648939372	0.80178168325106	0.999999980705644	0.616471365525818	0.944161179361028	abundance	52	47
Subdoligranulum	Treatment	Yeast:TimePost	TreatmentYeast:TimePost	0.565401585444057	1.03815715449253	0.590581272384809	0.999999980705644	2.30876606899627e-13	5.05042577592934e-12	abundance	52	47
Unclassified__Enterobacteriaceae	Sequence	Yeast_Placebo	SequenceYeast_Placebo	-0.19647401150131	0.794892009710106	0.809431282763904	0.999999980705644	0.809431282763904	0.966113797810981	abundance	52	52
Unclassified__Enterobacteriaceae	Time	Post:Period2	TimePost:Period2	-0.161402024964017	1.00420260442922	0.873298550934652	0.999999980705644	0.873298550934652	0.979661835984385	abundance	52	52
Unclassified__Enterobacteriaceae	Treatment	Yeast:TimePost	TreatmentYeast:TimePost	0.467590846463997	0.990580879501019	0.64007180170527	0.999999980705644	0.64007180170527	0.944161179361028	abundance	52	52
Unclassified__Lachnospiraceae	Time	Post:Period2	TimePost:Period2	-0.206833516491142	0.742663693553072	0.781931537941065	0.999999980705644	0.781931537941065	0.966113797810981	abundance	52	52
Unclassified__Lachnospiraceae	Treatment	Yeast	TreatmentYeast	-0.0377394260432957	0.530482074694964	0.943607318351629	0.999999980705644	0.943607318351629	0.992432429126749	abundance	52	52
Unclassified__Oscillospiraceae	Period	2	Period2	-0.174684357875029	0.552371770006781	0.7538763962821	0.999999980705644	0.939423171692914	0.992432429126749	abundance	52	48
Unclassified__Oscillospiraceae	Time	Post	TimePost	0.308127274675733	0.655445222402324	0.641633646889683	0.999999980705644	0.871573556958411	0.979661835984385	abundance	52	48
Unclassified__Oscillospiraceae	Time	Post:Period2	TimePost:Period2	-0.370735956946691	0.7158023923552	0.60827582554463	0.999999980705644	0.846552171147259	0.979300481328594	abundance	52	48
Unclassified__Peptostreptococcaceae	LogReadDepth	LogReadDepth	LogReadDepth	-0.121050059968345	0.290916611176208	0.679588276368752	0.999999980705644	0.270221995296412	0.6655200361714	abundance	52	49
Unclassified__Peptostreptococcaceae	Period	2	Period2	-0.342852816319019	0.837127394795899	0.685011932357643	0.999999980705644	0.900782517242934	0.991427298852286	abundance	52	49
Unclassified__Peptostreptococcaceae	Time	Post	TimePost	-0.171573226569142	0.989203006910926	0.863523753995213	0.999999980705644	0.981374234276441	0.992432429126749	abundance	52	49
Unclassified__Peptostreptococcaceae	Treatment	Yeast	TreatmentYeast	0.265280494126857	0.786228521421079	0.738209703681198	0.999999980705644	0.931465840753314	0.992432429126749	abundance	52	49
Unclassified__Ruminococcaceae	Period	2	Period2	-0.33642579989547	0.877891716917634	0.704264241111183	0.999999980705644	0.912540360914456	0.992432429126749	abundance	52	48
Unclassified__Ruminococcaceae	Time	Post	TimePost	0.208797461103397	0.936413278775953	0.825121417037924	0.999999980705644	0.969417481221176	0.992432429126749	abundance	52	48
Unclassified__Ruminococcaceae	Time	Post:Period2	TimePost:Period2	-0.414883375437326	1.10386759631651	0.709750119632131	0.999999980705644	0.915755006946438	0.992432429126749	abundance	52	48
[Eubacterium]_hallii_group	LogReadDepth	LogReadDepth	LogReadDepth	0.401165153084758	1.29912690484357	0.75747673470152	0.999999980705644	0.736294619154565	0.966113797810981	prevalence	52	51
[Eubacterium]_hallii_group	Period	2	Period2	5.24117618137775	203.022738310305	0.979404331077427	0.999999980705644	0.787741875430858	0.966113797810981	prevalence	52	51

[Eubacterium]_hallii_group	Sequence	Yeast_Placebo	SequenceYeast_Placebo	5.47088754635684	203.000765939068	0.978499547331181	0.999999980705644	0.748776047810638	0.966113797810981	prevalence	52	51
[Eubacterium]_hallii_group	Time	Post	TimePost	10.6573409123372	181.085869025562	0.953069658771906	0.999999980705644	0.986761386674596	0.992432429126749	prevalence	52	51
[Eubacterium]_hallii_group	Time	Post:Period2	TimePost:Period2	-3.47192901375947	5755.24946510262	0.999518665801534	0.999999980705644	0.983681222244613	0.992432429126749	prevalence	52	51
[Eubacterium]_hallii_group	Treatment	Yeast	TreatmentYeast	5.60843931084901	203.017166256825	0.977960888212923	0.999999980705644	0.923062206959692	0.992432429126749	prevalence	52	51
[Eubacterium]_hallii_group	Treatment	Yeast:TimePost	TreatmentYeast:TimePost	-0.999726659722682	8124.31453386041	0.999901817382688	0.999999980705644	0.882837856213378	0.984054935269689	prevalence	52	51
Akkermansia	LogReadDepth	LogReadDepth	LogReadDepth	0.159515755338097	0.499244621134192	0.749336610626712	0.999999980705644	0.779424803637846	0.966113797810981	prevalence	52	43
Akkermansia	Time	Post	TimePost	1.35409260515109	2.74406824400838	0.621686389194756	0.999999980705644	0.856878811879499	0.979300481328594	prevalence	52	43
Akkermansia	Time	Post:Period2	TimePost:Period2	-0.0572534312667551	2.25119939257256	0.979710058135349	0.999999980705644	0.65282001544391	0.944161179361028	prevalence	52	43
Akkermansia	Treatment	Yeast	TreatmentYeast	-4.91598534506386	9.06947935924572	0.587793627759873	0.999999980705644	0.406167414290857	0.836327970478074	prevalence	52	43
Alistipes	Period	2	Period2	36.7211945974377	14225549.354289	0.999997940376607	0.999999980705644	0.815010792596576	0.966113797810981	prevalence	52	48
Alistipes	Sequence	Yeast_Placebo	SequenceYeast_Placebo	-70.8135714043837	12259416.577368	0.99999539121173	0.999999980705644	0.512975382624854	0.880104823130878	prevalence	52	48
Alistipes	Time	Post	TimePost	-33.7984991761859	15395829.2837797	0.999998248402202	0.999999980705644	0.857847341278618	0.979300481328594	prevalence	52	48
Alistipes	Time	Post:Period2	TimePost:Period2	34.5066235250487	15395829.2837797	0.999998211703855	0.999999980705644	0.388255565456924	0.818611131987491	prevalence	52	48
Alistipes	Treatment	Yeast	TreatmentYeast	37.2360034757793	14225549.354289	0.999997911501937	0.999999980705644	0.117224549420979	0.442630270891951	prevalence	52	48
Alistipes	Treatment	Yeast:TimePost	TreatmentYeast:TimePost	151.120191961957	22353744.4604217	0.999994605983431	0.999999980705644	0.467089121988432	0.880104823130878	prevalence	52	48
Anaerostipes	LogReadDepth	LogReadDepth	LogReadDepth	0.159793013843536	0.435460893730491	0.713655219984341	0.999999980705644	0.646921870814297	0.944161179361028	prevalence	52	47
Anaerostipes	Period	2	Period2	0.605068592371958	1.58470684328529	0.702596836922542	0.999999980705644	0.0378671326269182	0.224377972265053	prevalence	52	47
Anaerostipes	Time	Post	TimePost	1.02824729083247	1.88921476243617	0.586254095735175	0.999999980705644	0.352314127255105	0.780442686957511	prevalence	52	47
Anaerostipes	Time	Post:Period2	TimePost:Period2	-0.711201712288135	2.13980326682235	0.739611546862377	0.999999980705644	0.371244715393625	0.802071915973881	prevalence	52	47
Anaerostipes	Treatment	Yeast	TreatmentYeast	0.719046062752569	1.48494312031747	0.628226444685965	0.999999980705644	0.861784423569162	0.979300481328594	prevalence	52	47
Anaerostipes	Treatment	Yeast:TimePost	TreatmentYeast:TimePost	-0.872338822130431	2.11478904316881	0.679977020454671	0.999999980705644	0.474075226386477	0.880104823130878	prevalence	52	47
Bifidobacterium	Treatment	Yeast	TreatmentYeast	11.4559165501947	21324054.8399902	0.999999571352681	0.999999980705644	0.766152348964583	0.966113797810981	prevalence	52	50
Christensenellaceae_R-7_group	Time	Post	TimePost	-0.232468705648006	1.54671851129374	0.880529507348803	0.999999980705644	0.698627996919176	0.966113797810981	prevalence	52	41
Clostridia_UCG-014	Period	2	Period2	0.154287872121796	1.89439232560032	0.935088445485741	0.999999980705644	0.759312810407233	0.966113797810981	prevalence	52	31
Clostridia_UCG-014	Sequence	Yeast_Placebo	SequenceYeast_Placebo	-0.675261164181375	2.56818087068905	0.792601721412544	0.999999980705644	0.737061409367731	0.966113797810981	prevalence	52	31
Clostridia_UCG-014	Time	Post	TimePost	-1.08662672781462	2.31430093092183	0.638693004288046	0.999999980705644	0.57854165920474	0.928851287714032	prevalence	52	31
Clostridia_UCG-014	Time	Post:Period2	TimePost:Period2	-0.0580981170350095	2.36288389258389	0.980383750393201	0.999999980705644	0.958692117313615	0.992432429126749	prevalence	52	31
Clostridia_UCG-014	Treatment	Yeast	TreatmentYeast	1.05480500982993	1.86791749069689	0.572280746791111	0.999999980705644	0.81705624043443	0.966113797810981	prevalence	52	31
Clostridia_UCG-014	Treatment	Yeast:TimePost	TreatmentYeast:TimePost	-0.0207930041608648	2.36680279735681	0.992990458012443	0.999999980705644	0.833836385750195	0.975336538215412	prevalence	52	31
Collinsella	Treatment	Yeast	TreatmentYeast	-0.412286950458191	0.985084593901276	0.675560031595181	0.999999980705644	0.113231204958748	0.440343574839574	prevalence	52	45
Dialister	Period	2	Period2	-0.480770271305543	1.50402389202894	0.749229112825032	0.999999980705644	0.149815013346349	0.52435254671222	prevalence	52	41
Dialister	Sequence	Yeast_Placebo	SequenceYeast_Placebo	0.229482136668167	1.67674298554548	0.891139999162365	0.999999980705644	0.182282555582906	0.543216626198354	prevalence	52	41
Dialister	Time	Post	TimePost	0.177543501582697	1.72792715478187	0.918161854914612	0.999999980705644	0.975470897642964	0.992432429126749	prevalence	52	41
Erysipelotrichaceae_UCG-003	Time	Post	TimePost	0.373095298920037	1.73860835461227	0.83008370289307	0.999999980705644	0.97112845197747	0.992432429126749	prevalence	52	44
Escherichia-Shigella	Period	2	Period2	-12.2852320796313	23188241.9487514	0.999999577277267	0.999999980705644	0.480859493617365	0.880104823130878	prevalence	52	50
Escherichia-Shigella	Time	Post	TimePost	0.821314444184554	33964031.5869426	0.999999980705644	0.999999980705644	0.815772237390866	0.966113797810981	prevalence	52	50
Faecalibacterium	LogReadDepth	LogReadDepth	LogReadDepth	4.56483781823029	8.5877059664775	0.59503425705596	0.999999980705644	0.836002747041782	0.975336538215412	prevalence	52	51
Faecalibacterium	Period	2	Period2	-8.4843462514471	18218157.3082109	0.999999628418573	0.999999980705644	0.979373043640293	0.992432429126749	prevalence	52	51
Faecalibacterium	Sequence	Yeast_Placebo	SequenceYeast_Placebo	-9.97530887848975	1149498.09613194	0.999993075982492	0.999999980705644	0.0768700406070688	0.357097156589484	prevalence	52	51

Faecalibacterium	Time	Post	TimePost	-9.2852451206655	18264712.3894468	0.999999594378846	0.999999980705644	0.47451079651628	0.880104823130878	prevalence	52	51
Faecalibacterium	Time	Post:Period2	TimePost:Period2	-9.18685811086016	18264719.3353146	0.999999598676984	0.999999980705644	0.406463820319365	0.836327970478074	prevalence	52	51
Faecalibacterium	Treatment	Yeast	TreatmentYeast	-0.901448365638267	18218040.9151193	0.999999960519809	0.999999980705644	0.0801405512892101	0.357097156589484	prevalence	52	51
Faecalibacterium	Treatment	Yeast:TimePost	TreatmentYeast:TimePost	2.53861411218827	18264452.8135805	0.999999889100373	0.999999980705644	0.340375776125001	0.776596405575682	prevalence	52	51
Parabacteroides	Period	2	Period2	0.00601747427815121	1.30081020569246	0.996309044411959	0.999999980705644	0.506634215169016	0.880104823130878	prevalence	52	41
Parabacteroides	Time	Post:Period2	TimePost:Period2	0.205302869527785	1.68962864857578	0.903288926214716	0.999999980705644	0.080706062361449	0.357097156589484	prevalence	52	41
Parabacteroides	Treatment	Yeast	TreatmentYeast	-0.59357805303341	1.19882726866152	0.620506764388381	0.999999980705644	0.0152989571101389	0.133865874713715	prevalence	52	41
Streptococcus	Period	2	Period2	27.124072569944	19775889.561929	0.999998905643225	0.999999980705644	0.541787865068931	0.896602389214837	prevalence	52	48
Streptococcus	Sequence	Yeast_Placebo	SequenceYeast_Placebo	-46.8512136492099	9862465.26785578	0.999996209683988	0.999999980705644	0.595780617839106	0.939293766863455	prevalence	52	48
Streptococcus	Time	Post	TimePost	-43.2819514487426	21772948.1810101	0.99999841390332	0.999999980705644	0.77671293280884	0.966113797810981	prevalence	52	48
Streptococcus	Time	Post:Period2	TimePost:Period2	22.1952782071175	21772990.5771021	0.999999186640451	0.999999980705644	0.999977830177978	0.999977830177978	prevalence	52	48
Streptococcus	Treatment	Yeast	TreatmentYeast	37.7233028893808	19685141.1234927	0.999998470986783	0.999999980705644	0.69890678616428	0.966113797810981	prevalence	52	48
Streptococcus	Treatment	Yeast:TimePost	TreatmentYeast:TimePost	10.5180401221266	22209461.9449557	0.999999622134843	0.999999980705644	0.595317976246758	0.939293766863455	prevalence	52	48
Subdoligranulum	Period	2	Period2	0.475215934851043	1.05002585403991	0.650854472138317	0.999999980705644	0.607006651489866	0.944161179361028	prevalence	52	47
Subdoligranulum	Time	Post	TimePost	-0.0703201985568993	1.00666177478045	0.944309196670617	0.999999980705644	0.71189811343249	0.966113797810981	prevalence	52	47
Unclassified__Oscillospiraceae	Period	2	Period2	-11.312848641022	333.747020967737	0.97295970151635	0.999999980705644	0.939423171692914	0.992432429126749	prevalence	52	48
Unclassified__Oscillospiraceae	Sequence	Yeast_Placebo	SequenceYeast_Placebo	-1.58358559917834	333.715294988827	0.996213797277838	0.999999980705644	0.693293359152294	0.966113797810981	prevalence	52	48
Unclassified__Oscillospiraceae	Time	Post	TimePost	-12.9017487288829	184.906239422001	0.944373107909344	0.999999980705644	0.871573556958411	0.979661835984385	prevalence	52	48
Unclassified__Oscillospiraceae	Time	Post:Period2	TimePost:Period2	16.3201309679073	184.931012948634	0.929678110956561	0.999999980705644	0.846552171147259	0.979300481328594	prevalence	52	48
Unclassified__Oscillospiraceae	Treatment	Yeast	TreatmentYeast	4.21641425871949	333.721524660018	0.989919372629755	0.999999980705644	0.08832445425649	0.376994621826482	prevalence	52	48
Unclassified__Oscillospiraceae	Treatment	Yeast:TimePost	TreatmentYeast:TimePost	9.07269340343763	673.833112186347	0.989257364851387	0.999999980705644	0.650429530375922	0.944161179361028	prevalence	52	48
Unclassified__Peptostreptococcaceae	Period	2	Period2	-1.7212797482279	124.548851154773	0.988973492680939	0.999999980705644	0.900782517242934	0.991427298852286	prevalence	52	49
Unclassified__Peptostreptococcaceae	Sequence	Yeast_Placebo	SequenceYeast_Placebo	1.00995006311624	124.545314581401	0.993529947442399	0.999999980705644	0.17123749226444	0.543216626198354	prevalence	52	49
Unclassified__Peptostreptococcaceae	Time	Post	TimePost	-0.082415966449205	1.55489377521648	0.957728527581592	0.999999980705644	0.981374234276441	0.992432429126749	prevalence	52	49
Unclassified__Peptostreptococcaceae	Time	Post:Period2	TimePost:Period2	11.5448962899735	129.286157013691	0.928845691782029	0.999999980705644	0.336236091768824	0.776596405575682	prevalence	52	49
Unclassified__Peptostreptococcaceae	Treatment	Yeast	TreatmentYeast	10.0537977932359	124.547228783829	0.935662424937195	0.999999980705644	0.931465840753314	0.992432429126749	prevalence	52	49
Unclassified__Peptostreptococcaceae	Treatment	Yeast:TimePost	TreatmentYeast:TimePost	-0.238775226257301	262.676602109889	0.999274716366948	0.999999980705644	0.754963547520116	0.966113797810981	prevalence	52	49
Unclassified__Ruminococcaceae	Period	2	Period2	2.40474265604227	910105.386647491	0.99999789177488	0.999999980705644	0.912540360914456	0.992432429126749	prevalence	52	48
Unclassified__Ruminococcaceae	Sequence	Yeast_Placebo	SequenceYeast_Placebo	-24.927210062723	910097.270630303	0.999978146252393	0.999999980705644	0.786067572838119	0.966113797810981	prevalence	52	48
Unclassified__Ruminococcaceae	Time	Post	TimePost	50.6603607164681	1819744.57390201	0.999977787476201	0.999999980705644	0.969417481221176	0.992432429126749	prevalence	52	48
Unclassified__Ruminococcaceae	Time	Post:Period2	TimePost:Period2	-49.6984952542962	1819744.57390124	0.999978209215391	0.999999980705644	0.915755006946438	0.992432429126749	prevalence	52	48
Unclassified__Ruminococcaceae	Treatment	Yeast	TreatmentYeast	3.0790003148341	910105.38664718	0.99999730065677	0.999999980705644	0.50888813185035	0.880104823130878	prevalence	52	48
Unclassified__Ruminococcaceae	Treatment	Yeast:TimePost	TreatmentYeast:TimePost	-30.0272035337948	1819550.89399593	0.999986832881574	0.999999980705644	0.795186725388668	0.966113797810981	prevalence	52	48
Bacteroides	LogReadDepth	LogReadDepth	LogReadDepth	1014.25366754602	36389.7374200922	0.977764264501567	NA	0.642470669758714	0.944161179361028	prevalence	52	51
Bacteroides	Period	2	Period2	-1850.91375918652	24784979.216392	0.99994041489817	NA	0.157495383545068	0.540425335693862	prevalence	52	51
Bacteroides	Sequence	Yeast_Placebo	SequenceYeast_Placebo	-2360.26131609635	16342760.9770625	0.9998847675702	NA	0.509242839153259	0.880104823130878	prevalence	52	51
Bacteroides	Time	Post	TimePost	-2095.76561093304	30338308.2259748	0.999944882258752	NA	0.507868781913501	0.880104823130878	prevalence	52	51
Bacteroides	Time	Post:Period2	TimePost:Period2	-1981.00749051893	32685471.7276196	0.999951641655849	NA	0.0384647952454377	0.224377972265053	prevalence	52	51
Bacteroides	Treatment	Yeast	TreatmentYeast	-322.940949324001	24784937.8981786	0.999989603782807	NA	0.0697499965737643	0.339062483344688	prevalence	52	51

Bacteroides	Treatment	Yeast:TimePost	TreatmentYeast:TimePost	423.94765442895	32685432.5528001	0.999989651007754	NA	0.649089298055435	0.944161179361028	prevalence	52	51
Bifidobacterium	Period	2	Period2	-23.8820465177403	21093104.7864408	0.999999096618711	NA	0.177401251369999	0.543216626198354	prevalence	52	50
Bifidobacterium	Sequence	Yeast_Placebo	SequenceYeast_Placebo	-25.9680404110193	16295620.5499931	0.999998728523504	NA	0.0554504699450719	0.27725234972536	prevalence	52	50
Bifidobacterium	Time	Post	TimePost	38.7928335402422	30011996.3593577	0.999998968672308	NA	0.183575928397079	0.543216626198354	prevalence	52	50
Bifidobacterium	Time	Post:Period2	TimePost:Period2	-38.1172794489386	30011996.3593577	0.999998986632265	NA	0.466126722183705	0.880104823130878	prevalence	52	50
Bifidobacterium	Treatment	Yeast:TimePost	TreatmentYeast:TimePost	21.9998279325355	29477692.8068205	0.999999404521814	NA	0.0027565488569115	0.0321597366639675	prevalence	52	50
Blautia	LogReadDepth	LogReadDepth	LogReadDepth	NA	NA	NA	NA	0.73916337633509	0.966113797810981	prevalence	52	52
Blautia	Period	2	Period2	NA	NA	NA	NA	0.502627543645612	0.880104823130878	prevalence	52	52
Blautia	Sequence	Yeast_Placebo	SequenceYeast_Placebo	NA	NA	NA	NA	0.201827617127797	0.566661363246377	prevalence	52	52
Blautia	Time	Post	TimePost	NA	NA	NA	NA	0.348482276577439	0.780442686957511	prevalence	52	52
Blautia	Time	Post:Period2	TimePost:Period2	NA	NA	NA	NA	0.964458763957116	0.992432429126749	prevalence	52	52
Blautia	Treatment	Yeast	TreatmentYeast	NA	NA	NA	NA	0.383377392310074	0.818183459198328	prevalence	52	52
Blautia	Treatment	Yeast:TimePost	TreatmentYeast:TimePost	NA	NA	NA	NA	0.108249131588748	0.430536318818883	prevalence	52	52
Clostridium_sensu_stricto_1	LogReadDepth	LogReadDepth	LogReadDepth	NA	NA	NA	NA	0.0452285671541576	0.2473437266243	prevalence	52	47
Clostridium_sensu_stricto_1	Period	2	Period2	NA	NA	NA	NA	0.796102568843332	0.966113797810981	prevalence	52	47
Clostridium_sensu_stricto_1	Sequence	Yeast_Placebo	SequenceYeast_Placebo	NA	NA	NA	NA	0.014517447886601	0.133713335797641	prevalence	52	47
Clostridium_sensu_stricto_1	Time	Post	TimePost	NA	NA	NA	NA	0.793964930981761	0.966113797810981	prevalence	52	47
Clostridium_sensu_stricto_1	Time	Post:Period2	TimePost:Period2	NA	NA	NA	NA	0.539133066293233	0.896602389214837	prevalence	52	47
Clostridium_sensu_stricto_1	Treatment	Yeast	TreatmentYeast	NA	NA	NA	NA	0.183990148853626	0.543216626198354	prevalence	52	47
Clostridium_sensu_stricto_1	Treatment	Yeast:TimePost	TreatmentYeast:TimePost	NA	NA	NA	NA	0.0416111283439729	0.23490153097404	prevalence	52	47
Escherichia-Shigella	LogReadDepth	LogReadDepth	LogReadDepth	21.6627580832739	21.6157389508369	0.31625897041376	NA	0.61928908020987	0.944161179361028	prevalence	52	50
Escherichia-Shigella	Sequence	Yeast_Placebo	SequenceYeast_Placebo	-67.1913748056348	16014494.727618	0.999996652347671	NA	0.890678539405205	0.986511040480449	prevalence	52	50
Escherichia-Shigella	Time	Post:Period2	TimePost:Period2	-57.6479692979544	35871196.671944	0.999998717733755	NA	0.651334804862846	0.944161179361028	prevalence	52	50
Escherichia-Shigella	Treatment	Yeast	TreatmentYeast	-82.6881522302317	22634558.786389	0.999997085182855	NA	0.770147735008556	0.966113797810981	prevalence	52	50
Escherichia-Shigella	Treatment	Yeast:TimePost	TreatmentYeast:TimePost	57.9166259805441	32948497.565716	0.999998597484405	NA	0.420553493726117	0.836327970478074	prevalence	52	50
Unclassified__Enterobacteriaceae	LogReadDepth	LogReadDepth	LogReadDepth	NA	NA	NA	NA	7.97972133993684e-06	0.000126950112226268	prevalence	52	52
Unclassified__Enterobacteriaceae	Period	2	Period2	NA	NA	NA	NA	0.0323175246195099	0.201984528871937	prevalence	52	52
Unclassified__Enterobacteriaceae	Sequence	Yeast_Placebo	SequenceYeast_Placebo	NA	NA	NA	NA	0.809431282763904	0.966113797810981	prevalence	52	52
Unclassified__Enterobacteriaceae	Time	Post	TimePost	NA	NA	NA	NA	0.415666344259143	0.836327970478074	prevalence	52	52
Unclassified__Enterobacteriaceae	Time	Post:Period2	TimePost:Period2	NA	NA	NA	NA	0.873298550934652	0.979661835984385	prevalence	52	52
Unclassified__Enterobacteriaceae	Treatment	Yeast	TreatmentYeast	NA	NA	NA	NA	0.121148323879069	0.442630270891951	prevalence	52	52
Unclassified__Enterobacteriaceae	Treatment	Yeast:TimePost	TreatmentYeast:TimePost	NA	NA	NA	NA	0.64007180170527	0.944161179361028	prevalence	52	52
Unclassified__Lachnospiraceae	LogReadDepth	LogReadDepth	LogReadDepth	NA	NA	NA	NA	0.098970033093949	0.412375137891454	prevalence	52	52
Unclassified__Lachnospiraceae	Period	2	Period2	NA	NA	NA	NA	0.0261142056106083	0.182799439274258	prevalence	52	52
Unclassified__Lachnospiraceae	Sequence	Yeast_Placebo	SequenceYeast_Placebo	NA	NA	NA	NA	0.524459328961706	0.891071675420375	prevalence	52	52
Unclassified__Lachnospiraceae	Time	Post	TimePost	NA	NA	NA	NA	0.211230090918427	0.566661363246377	prevalence	52	52
Unclassified__Lachnospiraceae	Time	Post:Period2	TimePost:Period2	NA	NA	NA	NA	0.781931537941065	0.966113797810981	prevalence	52	52
Unclassified__Lachnospiraceae	Treatment	Yeast	TreatmentYeast	NA	NA	NA	NA	0.943607318351629	0.992432429126749	prevalence	52	52
Unclassified__Lachnospiraceae	Treatment	Yeast:TimePost	TreatmentYeast:TimePost	NA	NA	NA	NA	0.474233918183439	0.880104823130878	prevalence	52	52
