# Supplementary material for: Effects of a Saccharomyces uvarum Cell-Based Preparation on Gut Microbial Ecology and Metabolic Activity in Adults Without Diagnosed Gastrointestinal Disease: A Randomized Placebo-Controlled Crossover Pilot Study
Source: Nutrients. 2026 Oct 15;18(20):3380. doi: 10.3390/nu18203380 (PMC13708381; doi:10.3390/nu18203380)
Supplement: Supplementary file 1 [file nutrients-18-03380-s001.zip › Supp_FigureS10_BIOEN_opls_score2d_0_dpi72.pdf]

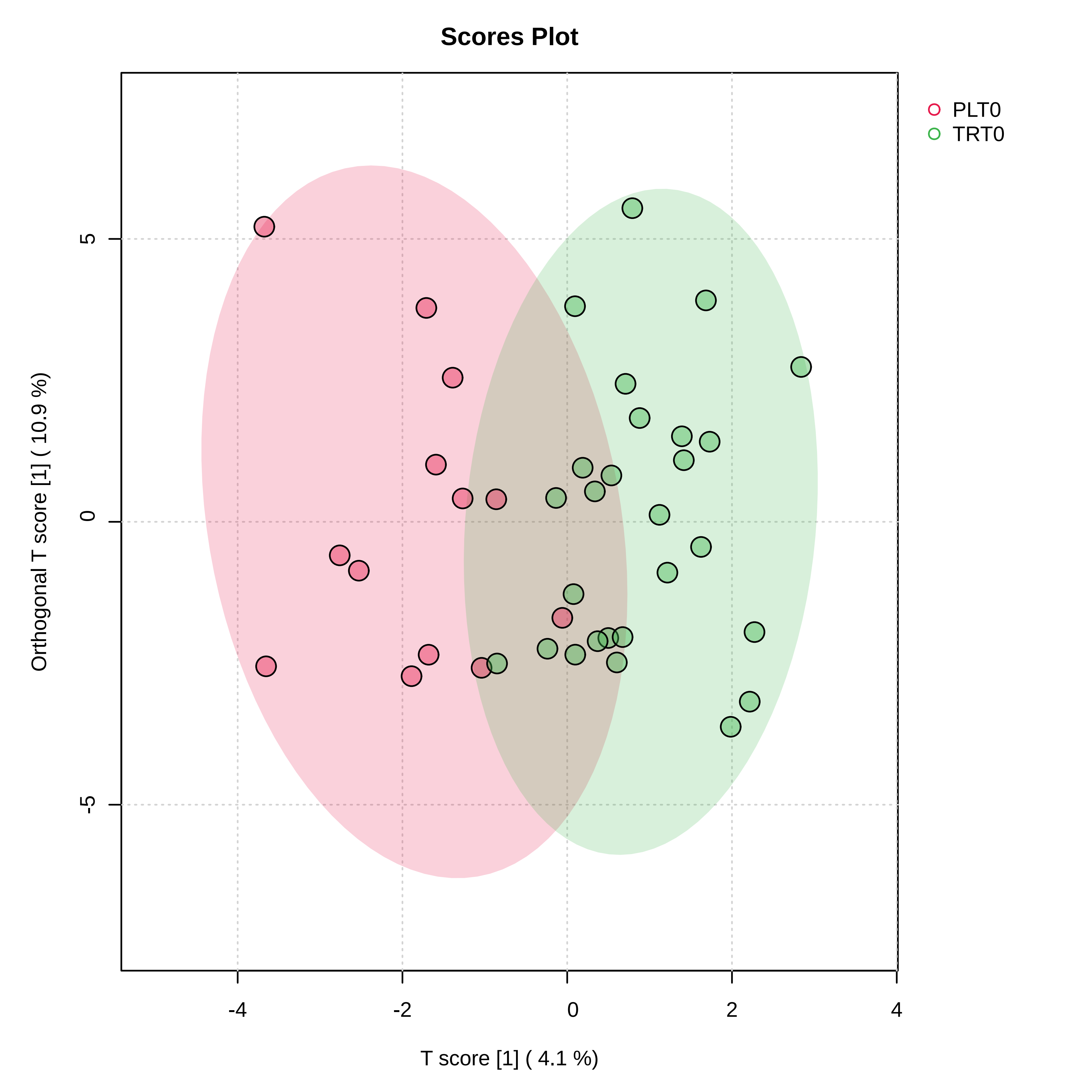
Scores Plot
PLT0
TRT0
Orthogonal T score [1] (10.9 %)
T score [1] (4.1 %)
5
0
-5
-4
-2
0
2
4
