# Supplementary material for: Effects of a Saccharomyces uvarum Cell-Based Preparation on Gut Microbial Ecology and Metabolic Activity in Adults Without Diagnosed Gastrointestinal Disease: A Randomized Placebo-Controlled Crossover Pilot Study
Source: Nutrients. 2026 Oct 15;18(20):3380. doi: 10.3390/nu18203380 (PMC13708381; doi:10.3390/nu18203380)
Supplement: Supplementary file 1 [file nutrients-18-03380-s001.zip › Supp_FigureS12_BIOEN_MaAsLin3_summary_plot.pdf]

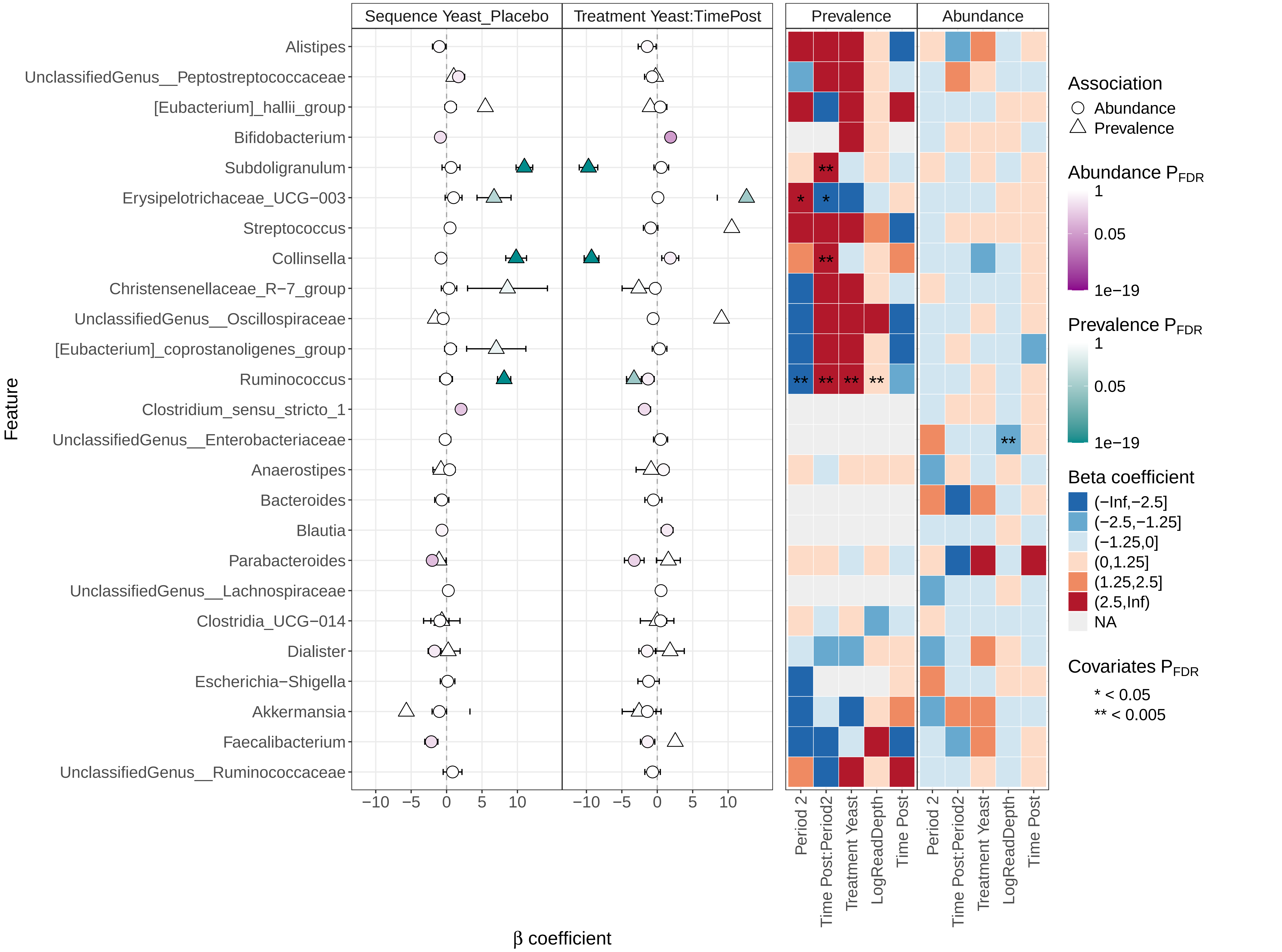

Sequence Yeast_Placebo
Treatment Yeast:TimePost
Prevalence
Abundance
Alistipes
UnclassifiedGenus__Peptostreptococcaceae
[Eubacterium]_hallii_group
Bifidobacterium
Subdoligranulum
Erysipelotrichaceae_UCG−003
Streptococcus
Collinsella
Christensenellaceae_R−7_group
UnclassifiedGenus__Oscillospiraceae
[Eubacterium]_coprostanoligenes_group
Ruminococcus
Clostridium_sensu_stricto_1
UnclassifiedGenus__Enterobacteriaceae
Anaerostipes
Bacteroides
Blautia
Parabacteroides
UnclassifiedGenus__Lachnospiraceae
Clostridia_UCG−014
Dialister
Escherichia−Shigella
Akkermansia
Faecalibacterium
UnclassifiedGenus__Ruminococcaceae
Feature
−10 −5 0 5 10
−10 −5 0 5 10
β coefficient
Period 2
Time Post:Period2
Treatment Yeast
LogReadDepth
Time Post
Period 2
Time Post:Period2
Treatment Yeast
LogReadDepth
Time Post
Association
Abundance
Prevalence
Abundance P FDR
1
0.05
1e−19
Prevalence P FDR
1
0.05
1e−19
Beta coefficient
(−Inf,−2.5]
(−2.5,−1.25]
(−1.25,0]
(0,1.25]
(1.25,2.5]
(2.5,Inf)
NA
Covariates P FDR
* < 0.05
** < 0.005
