# Supplementary material for: Effects of a Saccharomyces uvarum Cell-Based Preparation on Gut Microbial Ecology and Metabolic Activity in Adults Without Diagnosed Gastrointestinal Disease: A Randomized Placebo-Controlled Crossover Pilot Study
Source: Nutrients. 2026 Oct 15;18(20):3380. doi: 10.3390/nu18203380 (PMC13708381; doi:10.3390/nu18203380)
Supplement: Supplementary file 1 [file nutrients-18-03380-s001.zip › Supp_FigureS13_BIOEN_genus_abundance_descriptive.pdf]

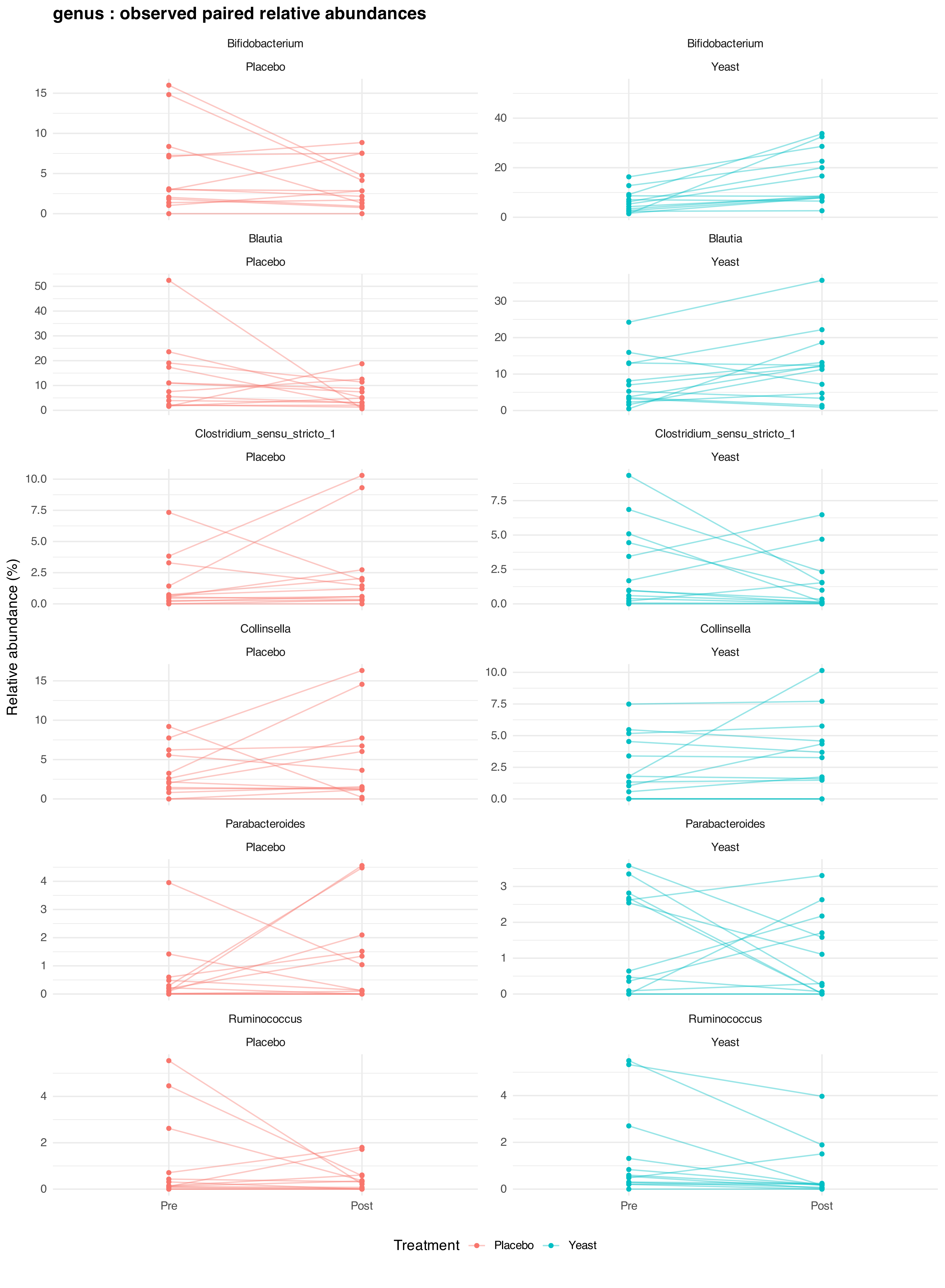
genus : observed paired relative abundances
Bifidobacterium
Placebo
Bifidobacterium
Yeast
Blautia
Placebo
Blautia
Yeast
Clostridium_sensu_stricto_1
Placebo
Clostridium_sensu_stricto_1
Yeast
Collinsella
Placebo
Collinsella
Yeast
Parabacteroides
Placebo
Parabacteroides
Yeast
Ruminococcus
Placebo
Ruminococcus
Yeast
Relative abundance (%)
Pre
Post
Treatment
Placebo
Yeast
