# Supplementary material for: Effects of a Saccharomyces uvarum Cell-Based Preparation on Gut Microbial Ecology and Metabolic Activity in Adults Without Diagnosed Gastrointestinal Disease: A Randomized Placebo-Controlled Crossover Pilot Study
Source: Nutrients. 2026 Oct 15;18(20):3380. doi: 10.3390/nu18203380 (PMC13708381; doi:10.3390/nu18203380)
Supplement: Supplementary file 1 [file nutrients-18-03380-s001.zip › Supp_FigureS14_BIOEN_genus_prevalence_forest_raw_p.pdf]

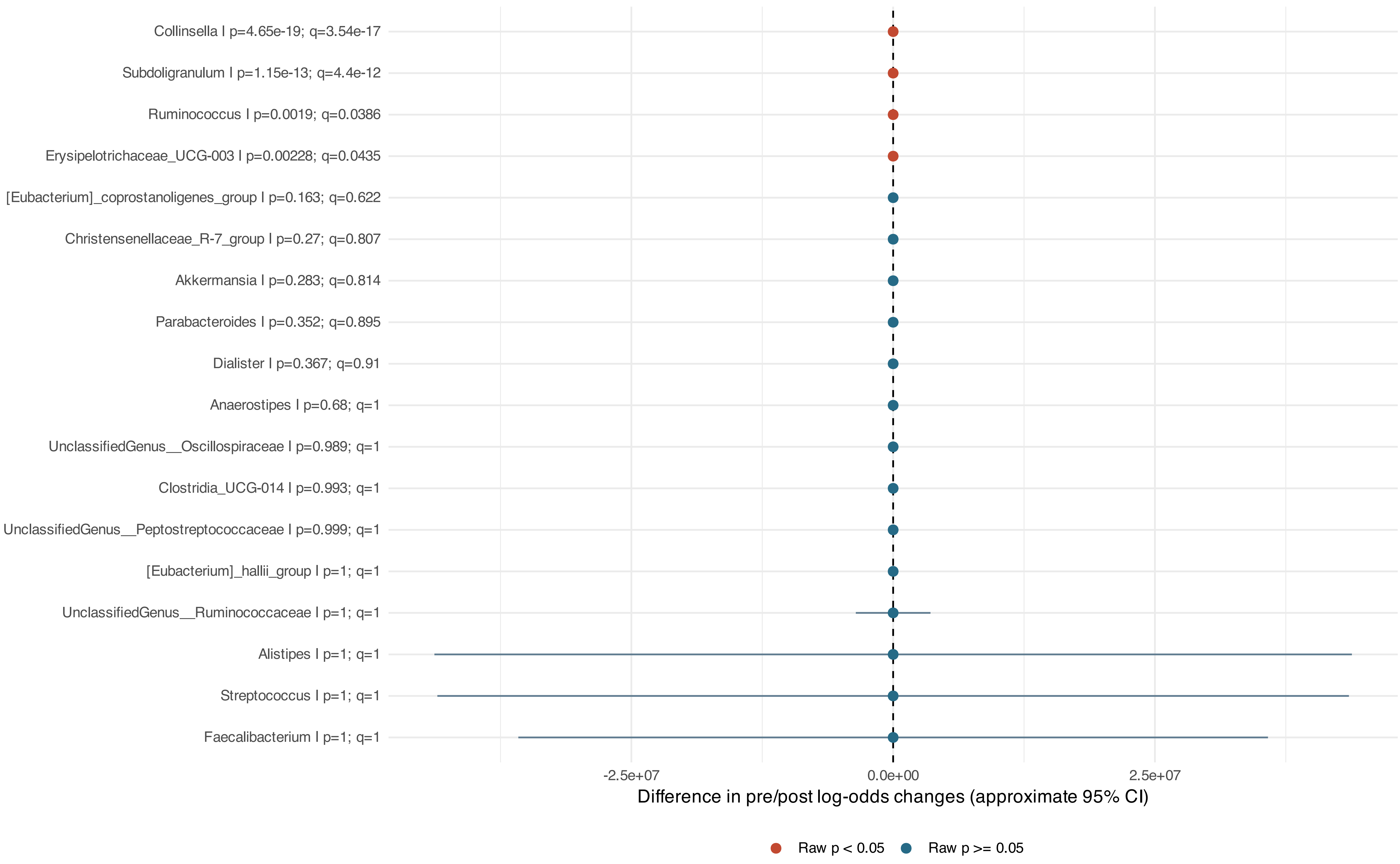
Collinsella | p=4.65e-19; q=3.54e-17
Subdoligranulum | p=1.15e-13; q=4.4e-12
Ruminococcus | p=0.0019; q=0.0386
Erysipelotrichaceae_UCG-003 | p=0.00228; q=0.0435
[Eubacterium]_coprostanoligenes_group | p=0.163; q=0.622
Christensenellaceae_R-7_group | p=0.27; q=0.807
Akkermansia | p=0.283; q=0.814
Parabacteroides | p=0.352; q=0.895
Dialister | p=0.367; q=0.91
Anaerostipes | p=0.68; q=1
UnclassifiedGenus__Oscillospiraceae | p=0.989; q=1
Clostridia_UCG-014 | p=0.993; q=1
UnclassifiedGenus__Peptostreptococcaceae | p=0.999; q=1
[Eubacterium]_hallii_group | p=1; q=1
UnclassifiedGenus__Ruminococcaceae | p=1; q=1
Alistipes | p=1; q=1
Streptococcus | p=1; q=1
Faecalibacterium | p=1; q=1
-2.5e+07
0.0e+00
2.5e+07
Difference in pre/post log-odds changes (approximate 95% CI)
Raw p < 0.05
Raw p >= 0.05
