# Supplementary material for: Effects of a Saccharomyces uvarum Cell-Based Preparation on Gut Microbial Ecology and Metabolic Activity in Adults Without Diagnosed Gastrointestinal Disease: A Randomized Placebo-Controlled Crossover Pilot Study
Source: Nutrients. 2026 Oct 15;18(20):3380. doi: 10.3390/nu18203380 (PMC13708381; doi:10.3390/nu18203380)
Supplement: Supplementary file 1 [file nutrients-18-03380-s001.zip › Supp_FigureS1_BIOEN_CONSORT_2025_flow_diagram.pdf]

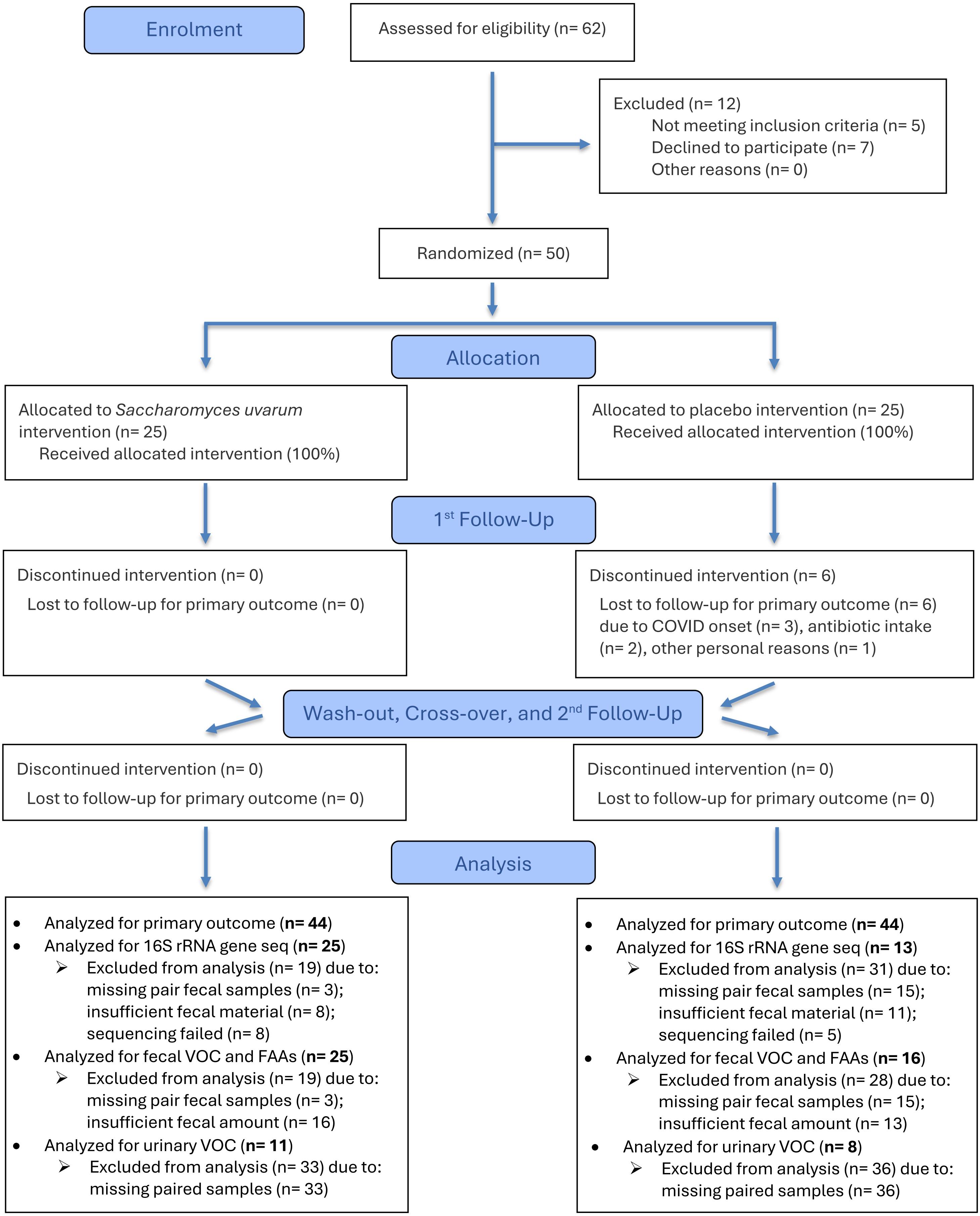

Enrolment
Assessed for eligibility (n= 62)
Excluded (n= 12)
Not meeting inclusion criteria (n= 5)
Declined to participate (n= 7)
Other reasons (n= 0)
Randomized (n= 50)
Allocation
Allocated to *Saccharomyces uvarum* intervention (n= 25)
Received allocated intervention (100%)
Allocated to placebo intervention (n= 25)
Received allocated intervention (100%)
1st Follow-Up
Discontinued intervention (n= 0)
Lost to follow-up for primary outcome (n= 0)
Discontinued intervention (n= 6)
Lost to follow-up for primary outcome (n= 6) due to COVID onset (n= 3), antibiotic intake (n= 2), other personal reasons (n= 1)
Wash-out, Cross-over, and 2nd Follow-Up
Discontinued intervention (n= 0)
Lost to follow-up for primary outcome (n= 0)
Discontinued intervention (n= 0)
Lost to follow-up for primary outcome (n= 0)
Analysis
Analyzed for primary outcome (**n= 44**)
Analyzed for 16S rRNA gene seq (**n= 25**)
Excluded from analysis (n= 19) due to: missing pair fecal samples (n= 3); insufficient fecal material (n= 8); sequencing failed (n= 8)
Analyzed for fecal VOC and FAAs (**n= 25**)
Excluded from analysis (n= 19) due to: missing pair fecal samples (n= 3); insufficient fecal amount (n= 16)
Analyzed for urinary VOC (**n= 11**)
Excluded from analysis (n= 33) due to: missing paired samples (n= 33)
Analyzed for primary outcome (**n= 44**)
Analyzed for 16S rRNA gene seq (**n= 13**)
Excluded from analysis (n= 31) due to: missing pair fecal samples (n= 15); insufficient fecal material (n= 11); sequencing failed (n= 5)
Analyzed for fecal VOC and FAAs (**n= 16**)
Excluded from analysis (n= 28) due to: missing pair fecal samples (n= 15); insufficient fecal amount (n= 13)
Analyzed for urinary VOC (**n= 8**)
Excluded from analysis (n= 36) due to: missing paired samples (n= 36)
