# Supplementary material for: Effects of a Saccharomyces uvarum Cell-Based Preparation on Gut Microbial Ecology and Metabolic Activity in Adults Without Diagnosed Gastrointestinal Disease: A Randomized Placebo-Controlled Crossover Pilot Study
Source: Nutrients. 2026 Oct 15;18(20):3380. doi: 10.3390/nu18203380 (PMC13708381; doi:10.3390/nu18203380)

| | pair | statistics | pval | FDR |
|---|---|---|---|---|
| Chao | TRT0 vs PLT0 | 1.9797 | 0.057 | 0.086 |
| | PLT0 vs PLT1 | 0.53827 | 0.595 | 0.595 |
| | TRT0 vs TRT1 | -1.0167 | 0.314 | 0.377 |
| | TRT1 vs PLT1 | **3.482** | **0.002** | **0.011** |
| Shannon | TRT0 vs PLT0 | 2.0449 | 0.055 | 0.164 |
| | PLT0 vs PLT1 | 0.4935 | 0.626 | 0.626 |
| | TRT0 vs TRT1 | 1.0114 | 0.317 | 0.380 |
| | TRT1 vs PLT1 | 1.668 | 0.115 | 0.230 |
| ACE | TRT0 vs PLT0 | 1.9679 | 0.059 | 0.088 |
| | PLT0 vs PLT1 | 0.53961 | 0.594 | 0.594 |
| | TRT0 vs TRT1 | -0.97248 | 0.336 | 0.403 |
| | TRT1 vs PLT1 | **3.4365** | **0.002** | **0.012** |
| Simpson | TRT0 vs PLT0 | 2.0237 | 0.061 | 0.197 |
| | PLT0 vs PLT1 | 0.71754 | 0.482 | 0.482 |
| | TRT0 vs TRT1 | 1.617 | 0.113 | 0.225 |
| | TRT1 vs PLT1 | 1.464 | 0.166 | 0.249 |
| Fisher | TRT0 vs PLT0 | 1.4905 | 0.149 | 0.224 |
| | PLT0 vs PLT1 | 0.50668 | 0.617 | 0.617 |
| | TRT0 vs TRT1 | -0.73854 | 0.464 | 0.557 |
| | TRT1 vs PLT1 | **2.9127** | **0.008** | **0.049** |

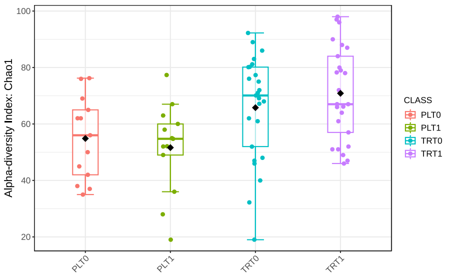


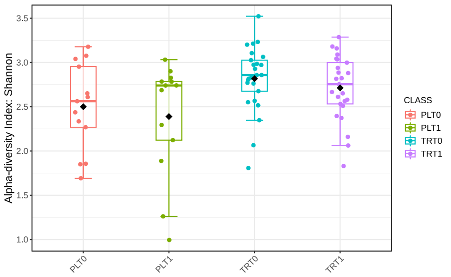


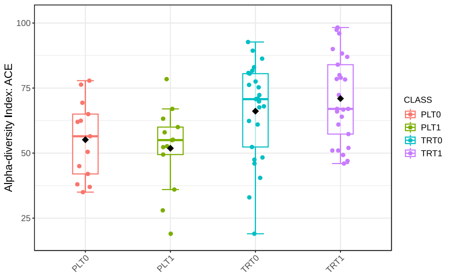


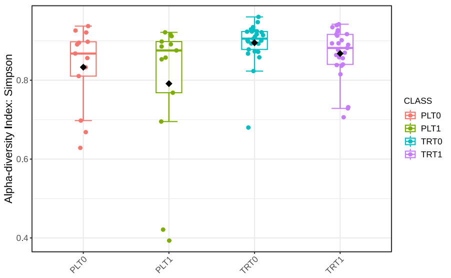


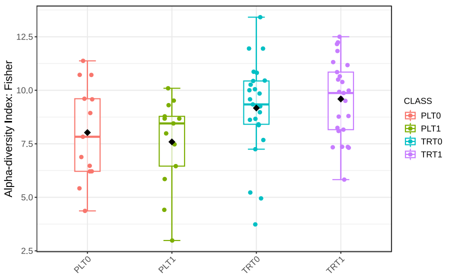

Supplement: Supplementary file 1 [file nutrients-18-03380-s001.zip › Supp_FigureS3_BIOEN_alpha_diversity.pdf]
