# Supplementary material for: Effects of a Saccharomyces uvarum Cell-Based Preparation on Gut Microbial Ecology and Metabolic Activity in Adults Without Diagnosed Gastrointestinal Disease: A Randomized Placebo-Controlled Crossover Pilot Study
Source: Nutrients. 2026 Oct 15;18(20):3380. doi: 10.3390/nu18203380 (PMC13708381; doi:10.3390/nu18203380)
Supplement: Supplementary file 1 [file nutrients-18-03380-s001.zip › Supp_FigureS4_BIOEN_volcano.pdf]

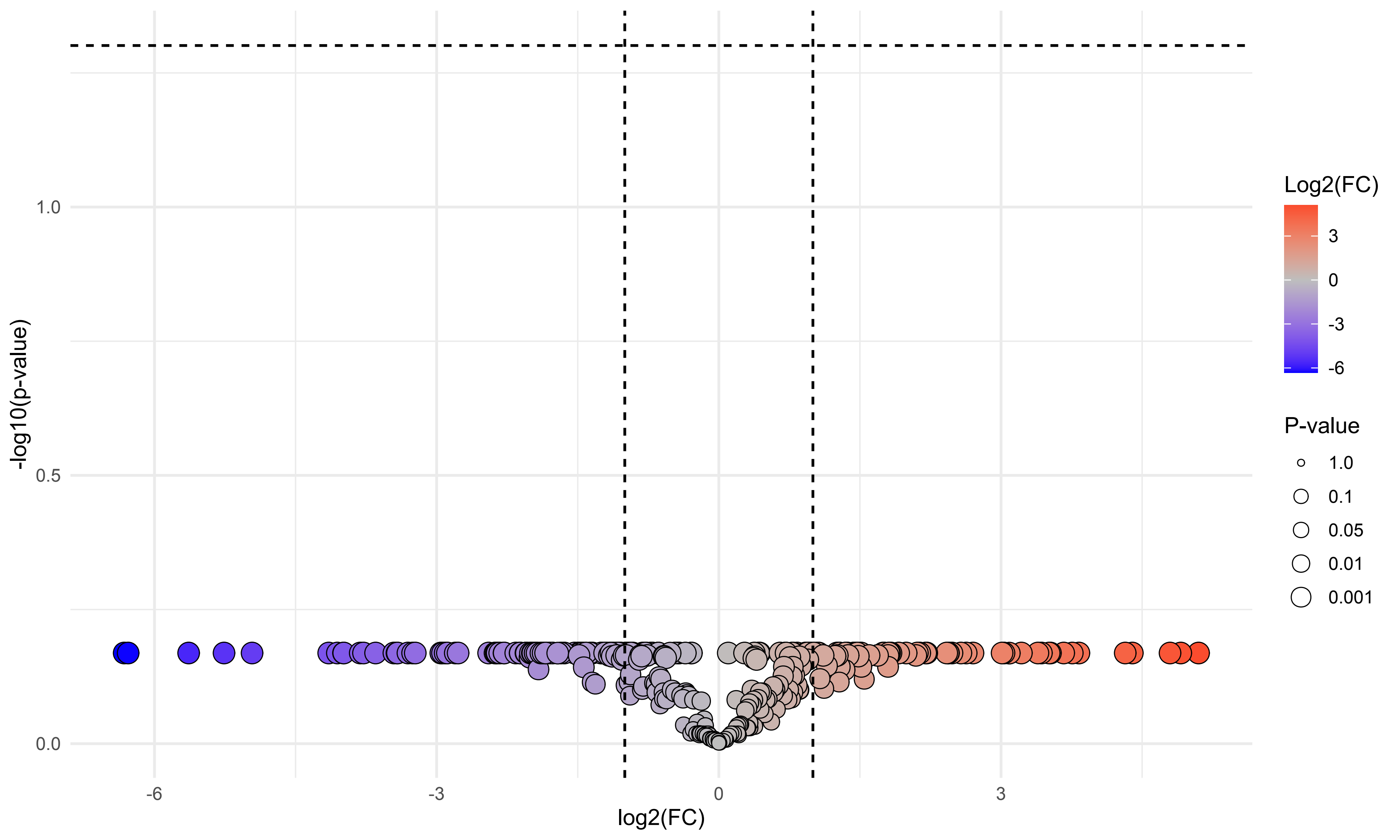

-log10(p-value)
1.0
0.5
0.0
-6
-3
0
3
log2(FC)
Log2(FC)
3
0
-3
-6
P-value
1.0
0.1
0.05
0.01
0.001
