# Supplementary material for: Effects of a Saccharomyces uvarum Cell-Based Preparation on Gut Microbial Ecology and Metabolic Activity in Adults Without Diagnosed Gastrointestinal Disease: A Randomized Placebo-Controlled Crossover Pilot Study
Source: Nutrients. 2026 Oct 15;18(20):3380. doi: 10.3390/nu18203380 (PMC13708381; doi:10.3390/nu18203380)
Supplement: Supplementary file 1 [file nutrients-18-03380-s001.zip › Supp_FigureS6_BIOEN_family.pdf]

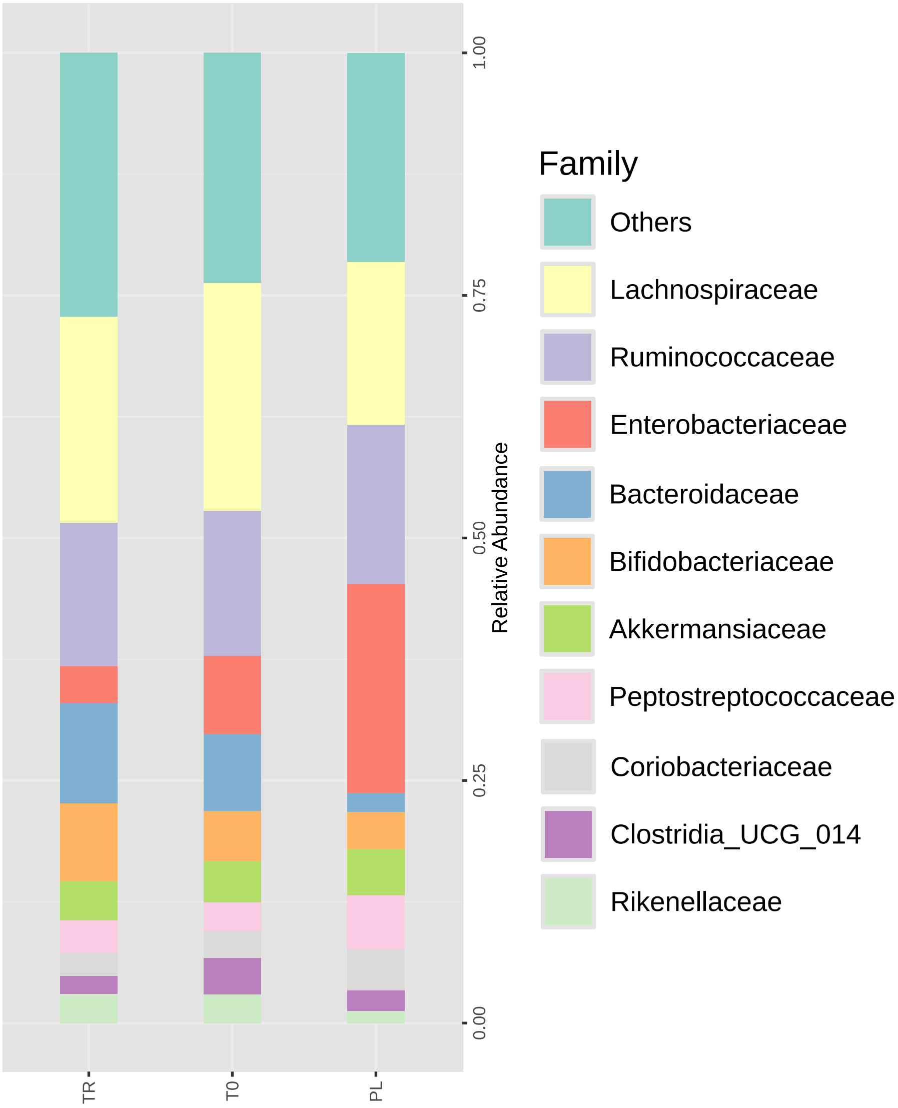
Family
Others
Lachnospiraceae
Ruminococcaceae
Enterobacteriaceae
Bacteroidaceae
Bifidobacteriaceae
Akkermansiaceae
Peptostreptococcaceae
Coriobacteriaceae
Clostridia_UCG_014
Rikenellaceae
Relative Abundance
1.00
0.75
0.50
0.25
0.00
TR
T0
PL
