# Supplementary material for: Effects of a Saccharomyces uvarum Cell-Based Preparation on Gut Microbial Ecology and Metabolic Activity in Adults Without Diagnosed Gastrointestinal Disease: A Randomized Placebo-Controlled Crossover Pilot Study
Source: Nutrients. 2026 Oct 15;18(20):3380. doi: 10.3390/nu18203380 (PMC13708381; doi:10.3390/nu18203380)
Supplement: Supplementary file 1 [file nutrients-18-03380-s001.zip › Supp_FigureS7_BIOEN_genus.pdf]

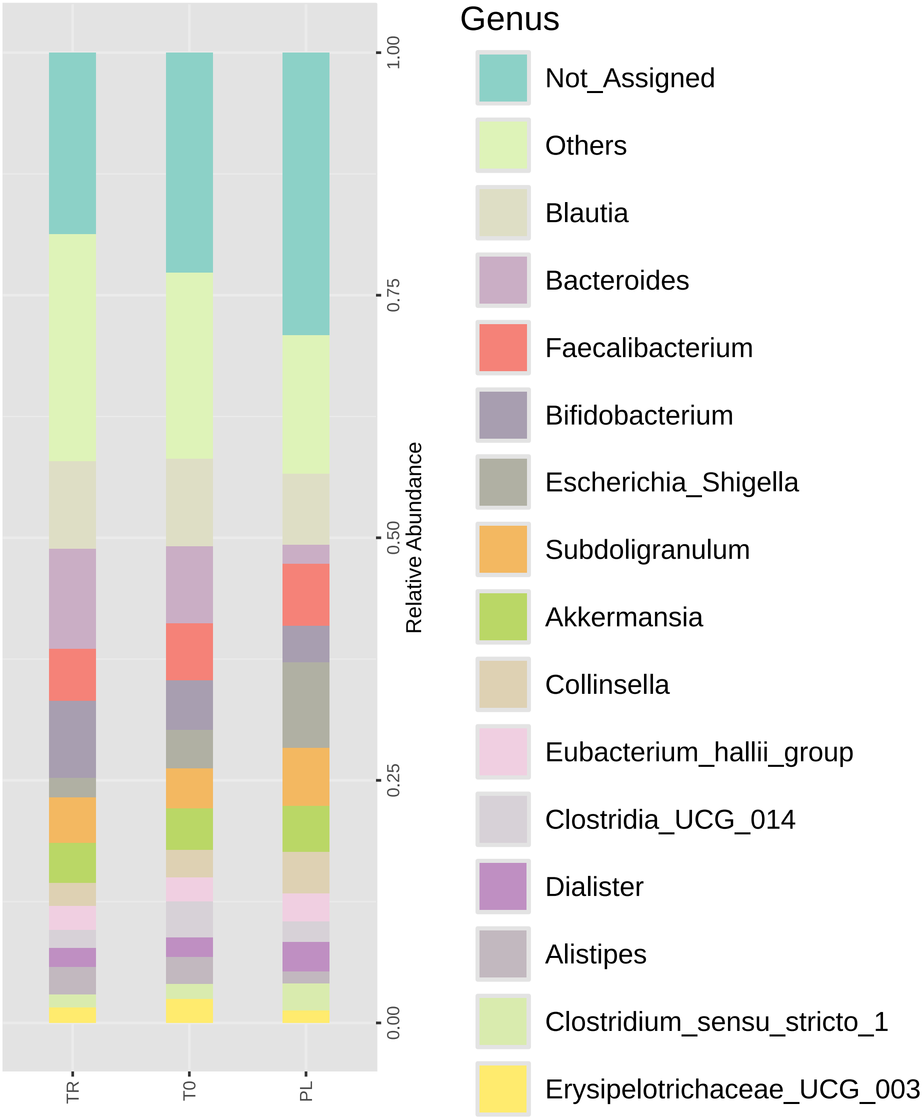
Genus
Not_Assigned
Others
Blautia
Bacteroides
Faecalibacterium
Bifidobacterium
Escherichia_Shigella
Subdoligranulum
Akkermansia
Collinsella
Eubacterium_hallii_group
Clostridia_UCG_014
Dialister
Alistipes
Clostridium_sensu_stricto_1
Erysipelotrichaceae_UCG_003
Relative Abundance
0.00
0.25
0.50
0.75
1.00
TR
T0
PL
