# Supplementary material for: Effects of a Saccharomyces uvarum Cell-Based Preparation on Gut Microbial Ecology and Metabolic Activity in Adults Without Diagnosed Gastrointestinal Disease: A Randomized Placebo-Controlled Crossover Pilot Study
Source: Nutrients. 2026 Oct 15;18(20):3380. doi: 10.3390/nu18203380 (PMC13708381; doi:10.3390/nu18203380)
Supplement: Supplementary file 1 [file nutrients-18-03380-s001.zip › Supp_FigureS8_LEfSe_TRT1vsPLT1.pdf]

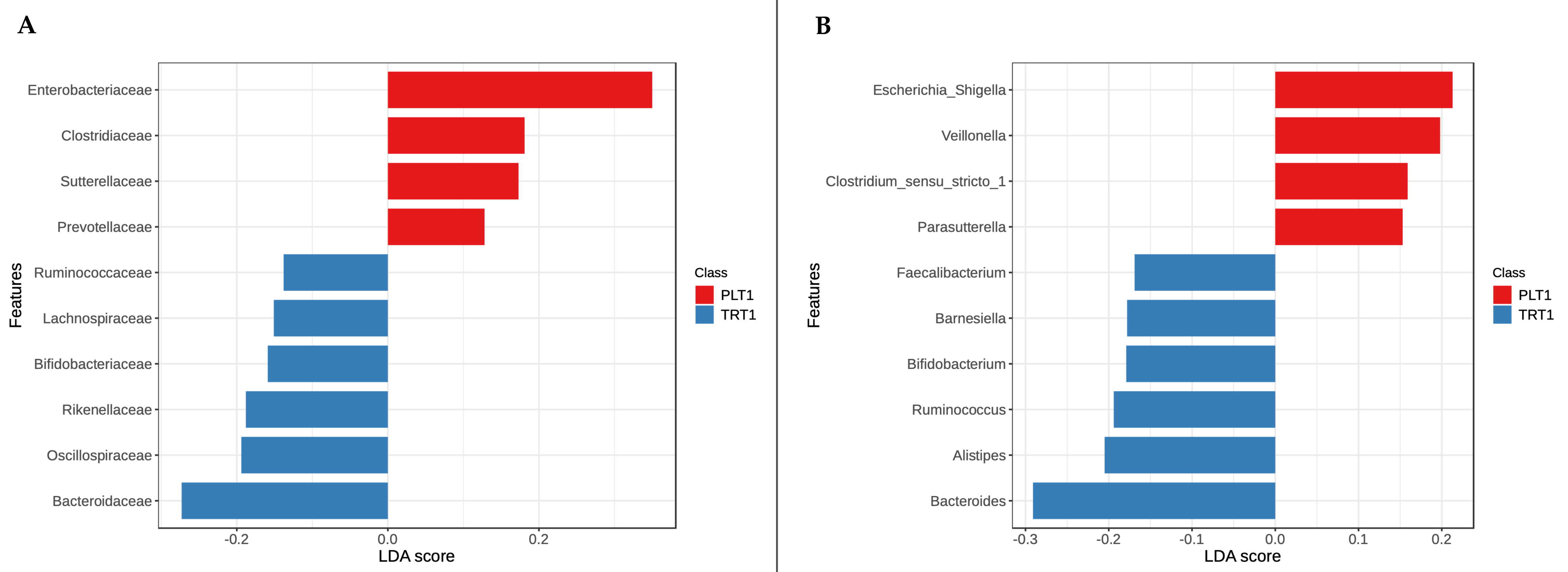
A
Enterobacteriaceae
Clostridiaceae
Sutterellaceae
Prevotellaceae
Ruminococcaceae
Lachnospiraceae
Bifidobacteriaceae
Rikenellaceae
Oscillospiraceae
Bacteroidaceae
Features
-0.2
0.0
0.2
LDA score
Class
PLT1
TRT1
B
Escherichia_Shigella
Veillonella
Clostridium_sensu_stricto_1
Parasutterella
Faecalibacterium
Barnesiella
Bifidobacterium
Ruminococcus
Alistipes
Bacteroides
Features
-0.3
-0.2
-0.1
0.0
0.1
0.2
LDA score
Class
PLT1
TRT1
