# Supplementary material for: Effects of a Saccharomyces uvarum Cell-Based Preparation on Gut Microbial Ecology and Metabolic Activity in Adults Without Diagnosed Gastrointestinal Disease: A Randomized Placebo-Controlled Crossover Pilot Study
Source: Nutrients. 2026 Oct 15;18(20):3380. doi: 10.3390/nu18203380 (PMC13708381; doi:10.3390/nu18203380)
Supplement: Supplementary file 1 [file nutrients-18-03380-s001.zip › Supp_FigureS9_permutation_PLSDA.pdf]

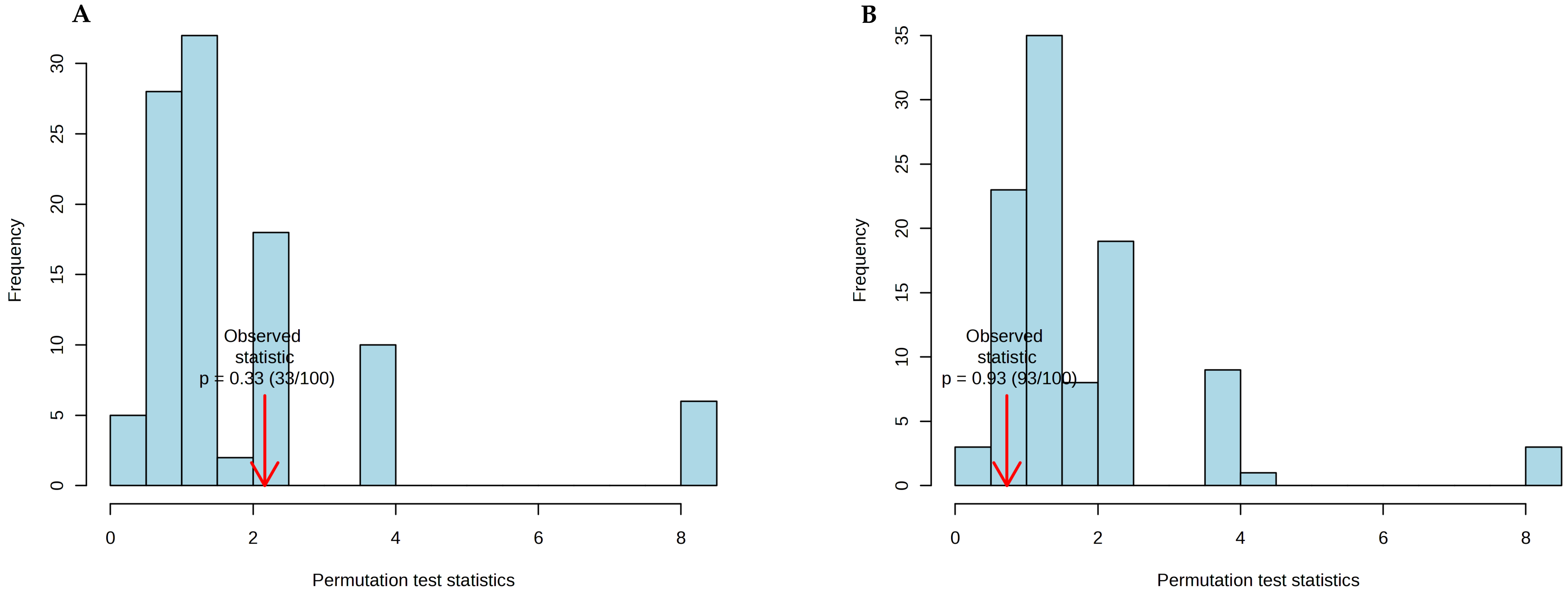
A
Frequency
Observed
statistic
p = 0.33 (33/100)
Permutation test statistics
B
Frequency
Observed
statistic
p = 0.93 (93/100)
Permutation test statistics
